# Key subdomains of mesencephalic astrocyte-derived neurotrophic factor attenuate myocardial ischemia/reperfusion injury by JAK1/STAT1/NF-κB signaling pathway

**DOI:** 10.1186/s10020-024-00916-6

**Published:** 2024-09-06

**Authors:** Haibin Dong, Wenjuan Jia, Chunxiao Wang, Da Teng, Bowen Xu, Xiaoning Ding, Jun Yang, Lin Zhong, Lei Gong

**Affiliations:** 1 Department of Cardiology, Yantai Yuhuangding Hospital, Qingdao University, No. 20 Yudong Road, Yantai City, Shandong Province 264000 China; 2 Shanghai Children’s Medical Center, Shanghai Jiao Tong University School of Medicine, Shanghai, 200127 China

**Keywords:** I/R, MANF, Apoptosis, ER stress, JAK1/STAT1/NF-κB pathway

## Abstract

**Background:**

Myocardial ischemia/reperfusion (I/R) injury is a common pathological process in clinical practice. Developing effective therapeutic strategies to reduce or prevent this injury is crucial. The article aimed to investigate the role and mechanism of mesencephalic astrocyte-derived neurotrophic factor (MANF) and its key subdomains in modulating myocardial I/R-induced cardiomyocyte apoptosis.

**Methods:**

MANF stable knockout cell line and MANF mutant overexpression plasmids were constructed. The effects of MANF and mutants on apoptosis and endoplasmic reticulum (ER) stress related proteins were evaluated in hypoxia/reoxygenation-induced HL-1 cardiomyocytes by western blot, immunofluorescence, Tunel and flow cytometry. Echocardiography, ELISA, TTC and Masson were used to observe the effects of recombinant MANF protein (rMANF) on cardiac function in myocardial I/R mice.

**Results:**

This study observed increased expression of MANF in both myocardial infarction patients and I/R mice. MANF overexpression in cardiomyocytes decreased ER stress-induced apoptosis, while MANF knockout exacerbated it. rMANF improved cardiac function in I/R mice by reducing injury and inflammation. This study specifically demonstrates that mutations in the α-helix of MANF were more effective in reducing ER stress and cardiomyocyte apoptosis. Mechanistically, MANF and the α-helix mutant attenuated I/R injury by inhibiting the JAK1/STAT1/NF-κB signaling pathway in addition to reducing ER stress-induced apoptosis.

**Conclusion:**

These findings highlight MANF and its subdomains as critical regulators of myocardial I/R injury, offering promising therapeutic targets with significant clinical implications for I/R-related diseases.

**Supplementary Information:**

The online version contains supplementary material available at 10.1186/s10020-024-00916-6.

## Introduction

Mesencephalic astrocyte-derived neurotrophic factor (MANF) was initially discovered in rat midbrain cells derived from type 1 astrocytes (Shen et al. 2012). MANF is found in various organs and tissues such as the heart, brain, liver and spleen (Danilova and Lindahl 2018). It acts as an endoplasmic reticulum (ER) stress response protein, being produced and released by cells and tissues in reaction to hypoxic, ischemic, or traumatic damage to provide protection against ER stress-induced injury (Greer et al. 2022). MANF has protective effects against heart disease, diabetes and immunomodulation in addition to its function in the nervous system (Kovaleva et al. 2023; Liu et al. 2022). Studies have shown that increased MANF levels during myocardial infarction act similarly to cardiomyokine in cardiomyocytes, potentially influencing cardiovascular function (Maciel et al. 2021). Moreover, the administration of rMANF has been shown to alleviate ER stress and reduce apoptosis, indicating its role as a secreted cardiomyokine induced by ER stress in promoting myocardial survival following ischemic injury (Zeng et al. 2023). Despite these findings, the precise pathological mechanism of myocardial ischemia/reperfusion (I/R) injury mediated by MANF remains unclear.

Crystal structure analysis of MANF reveals that it consists of an amino (N)-terminal saposin-like structural domain with five α-helices (α1-α5) and a carboxylic (C)-terminal SAP-like structural domain with α-helices (α6-α8). These two structural domains are relatively independent but connected by a flexible peptide segment (Sivakumar and Krishnan 2023). Similar to the KDEL motif found in ER-resident proteins, the RTDL motif in MANF’s C-terminal region is crucial for ER retention (Mätlik et al. 2015). The α-helix, a common secondary structure in proteins, plays a significant role in biological processes and protein interactions (Zhang et al. 2023a, b). While current studies have focused on the CKGC and RTDL sequences in MANF, the specific role of the α-helix motifs in its survival-promoting functions remains underexplored, offering a promising avenue for future research (Mätlik et al. 2015; Božok et al. 2018).

The morbidity and mortality of myocardial infarction (MI) are increasing globally, posing a significant threat to human health (Schäfer et al. 2022; Jiang et al. 2023). Myocardial I/R injury triggers a variety of adverse intracellular effects including mitochondrial damage and ER stress, leading to cell death, myocardial injury, cardiac dysfunction, coronary artery spasm and cardiac arrest (Algoet et al. 2023). Apoptosis, inflammation and oxidative stress are all pivotal in the pathogenesis of myocardial ischemia (Qi et al. 2022). Proper protein folding in the ER of cardiomyocytes is crucial for maintaining cardiac function. Under various pathological conditions like ischemia, hypertrophy, or heart failure, misfolded proteins accumulate in the ER lumen of cardiomyocytes, triggering the unfolded protein response (UPR) (Cominacini et al. 2015; Zhu and Zhou 2021). Excessive and prolonged ER stress leads to cell death through mechanisms like autophagy, apoptosis, and inflammation. Upon I/R occurrence, an immediate inflammatory response is initiated, persisting during reperfusion, followed by the release of inflammatory cytokines (Li et al. 2023). MANF is preserved or even enhanced intracellularly during ER stress to shield cardiomyocytes from I/R injury (Fu et al. 2020; Wu et al. 2021). Hence, reducing apoptosis, inflammatory response, and oxidative stress in myocardial tissue during I/R is crucial in mitigating myocardial I/R injury (Shi et al. 2023). Despite decades of development, there remains a lack of effective therapeutic drugs and molecular targets for mitigating myocardial I/R injury (Trujillo-Rangel et al. 2022).

In the present study, it was observed that MANF decreased cardiomyocyte apoptosis by mitigating ER stress. Simultaneously, the critical helix responsible for regulating MANF expression and secretion was identified to elucidate its protective function in myocardial injury induced by I/R. Furthermore, we delved into the molecular mechanism underlying the protective impact of MANF against myocardial I/R injury.

## Materials and methods

### Human samples and ethical statement

In this study, blood samples were collected from 20 acute myocardial infarction patients within 24 h of onset at Yantai Yuhuangding Hospital. Additionally, 20 healthy blood samples were used as a control group. Informed consent was obtained from all participants, and the study was approved by the Ethics Committee of Yantai Yuhuangding Hospital.

### Animal treatment

Male C57BL/6 mice, 8 weeks old, were obtained from Pengyue Laboratory Animal Breeding Co. Ltd (Jinan, China) and housed in an SPF-level laboratory animal room. A myocardial I/R model was constructed as described in the literature (Li et al. 2020). Briefly, mice were anesthetized with a small animal anesthesia machine (Shenzhen Ruiwode Lift Technology Co., Ltd. China, R5301E), and the left anterior descending coronary artery was ligated after thoracotomy. After 30 min, the ligature was loosened for 24 h of reperfusion. Mice in the sham operation group underwent the same procedure without ligation. The study included three groups of animals (*n* = 10 per group): I/R-treated mice (I/R), I/R-treated mice receiving intracardiac injection of recombinant human MANF protein (1.5 mg/kg, Novoprotein, Suzhou, China) (I/R + rMANF), and control group mice (Sham). All animal experiments and protocols were approved by the Animal Management and Use Committee of Yantai Yuhuangding Hospital.

### Cell culture and construction of H/R

The mouse cardiomyocyte cell line (HL-1) was obtained from the Institute of Cell Biology, Chinese Academy of Sciences (Shanghai, China). HL-1 were cultured in MEM medium supplemented with 10% FBS, 100 IU/ml penicillin and 100ug/ml streptomycin in an incubator at 37 °C with 5% CO_2_. To mimic myocardial I/R injury in vitro, HL-1 were subjected to H/R treatment. Initially, HL-1 were allowed to grow in a three-gas cell culture chamber with 5% CO_2_, 1% O_2_, and 94% N_2_ at 37 °C for 20 h. Subsequently, HL-1 were returned to normal culture conditions (95% air and 5% CO_2_) for an additional 3 h. Control cells were cultured under normal conditions.

### Construction and transfection of plasmids

Plasmids for Wt (wild-type MANF) and M1-M8 mutant (proline inserted between α1-α8 to disrupt α-helix) were constructed by subcloning the full-length cDNA into the pcDNA3.1(+) expression vector, with the addition of an HA tag at the 3′ end of MANF using PCR. The integrity of all plasmids was confirmed through DNA sequencing. Transfection of the plasmids was performed following the instructions of the Lipofectamine 3000 transfection reagent (Invitrogen, America). A mixture of plasmid and p3000 at a 1:2 ratio was prepared, followed by the addition of Lipofectamine 3000 (plasmid : lip3000 = 1:2). After mixing for 10–15 min, the resulting solution was added to the cells and cultured for 24 h, then transferred to an anoxic incubator for an additional 20 h.

### Construction of shMANF

Small hairpin RNA targeting human MANF (shMANF) and negative control shRNA (shNC) were cloned into miRZip™ shRNA expression stimulation vectors (Systembio, Shanghai, China). The target sequences of MANF were 5′-TATCTTCCGGATATAGTCAG′ (sh1) and 5′- GGACCTCAAAGACAGAGATTT-3′ (sh2). A stable HL-1 cell line with knockout of MANF was established by lentiviral infection. Lentiviral particles were generated from HEK293T cells transfected with the packaging plasmid psPAX2, envelope plasmid pMD2.G and the target plasmid. Plasmids containing shNC and shMANF were introduced into HL-1 by transfection using Lipofectamine 3000 transfection reagent following the manufacturer’s instructions. After incubation of the cells for 48–72 h in the presence of lentivirus, different concentrations of puromycin were added to aid in the identification of cells with positive features.

### Cell viability assay

Cell viability was determined using the CCK-8 assay. Specifically, cells were seeded at a density of 1 × 10^4^ cells per well in 96-well plates and incubated for 24 h. CCK-8 reagent (Vazyme, China) was then added at a concentration of 1/10 of the medium volume, followed by a 2-hour incubation at 37 °C. Absorbance at 450 nm was measured using a multimode plate reader (VICTOR Nivo™, Finland) to obtain the results.

### Western blot

Proteins were extracted from cardiomyocytes or cardiac tissues using RIPA lysate (Sparkjade, EA0002, China) and quantified with a BCA protein assay kit (Yeasen, 20201ES86, China). Subsequently, the protein samples underwent separation by sodium dodecyl sulfate-polyacrylamide gel electrophoresis and transfer to nitrocellulose membranes. These membranes were blocked with 5% skimmed milk powder for 1 h at room temperature, followed by overnight incubation with primary antibodies at 4 °C. After that, the membranes were exposed to secondary anti-rabbit or anti-mouse polyclonal antibodies (1:10000, D110087, D110058, Sangon, China) at room temperature for 1 h. Imaging and analysis were performed using a chemiluminescence imaging system (Clinx, ChemiScope 6000, China) and relative protein expression levels in different groups were determined using Image J software. GAPDH (1:10000, D110016, Sangon, China) and β-Tubulin (1:5000, 10094, Proteintech, China) served as internal references. The antibodies utilized in the study included MANF antibody (1:1000, ab67271, Abcam, America), Bcl-2 antibody (1:500, T40056, Abmart, China), Bax antibody (1:500, T40051, Abmart, China), Cleaved-Caspase3 antibody (1:1000, 9664, CST, America), HA-tag antibody (1:1000, 3724, CST, America), ATF6 antibody (1:1000, 65880, CST, America), p-IRE1α antibody (1:500, TD8322, Abmart, China), p-PERK antibody (1:500, TD8322, Abmart, China), BiP antibody (1:1000, 3177, CST, America), p-eIF2α antibody (1:500, 3398, CST, America), ATF4 antibody (1:500, T55873, Abmart, China), CHOP antibody (1:1000, 2895, CST, America), LC3B antibody (1:500, 381544, Zenbio, China), SQSTM1 antibody (1:500, YT7058, Immunoway, China), JAK1 antibody (1:500, YT2424, Immunoway, China), p-JAK1 antibody (1:500, YP0154, Immunoway, China), STAT1 antibody (1:1000, 14994, CST, America), p-STAT1 antibody (1:1000, 9167, CST, America), NF-κB antibody (1:1000, 8242, CST, America), and p-NF-κB antibody (1:1000, 3033, CST, America).

### Real-time quantitative PCR (qRT-PCR)

Total RNA was extracted from cells or tissues samples using RNA extraction reagent (Vazume, China) according to the manufacturer’s instructions. RNA samples were reverse transcribed using the HiScript III All-in-one RT SuperMix Perfect for qPCR (Vazume, America). qRT-PCR was performed on an Applied Biosystems 7500 (Thermo Fisher Scientific, America) using ChamQ Universal SYBR qPCR Master Mix (Vazume, China). The relative RNA level was analyzed by using 2^−ΔΔct^ method, with GAPDH serving as the internal reference. The MANF primer pairs were custom-synthesized by Sangon: forward 5′-TCAATGAGGTGTCGAAGCCC-3′, reverse 5′-GTCCACTGTGCTCAGGTCAA 3′.

### Immunofluorescence

Cell cultures or confocal petri dishes were fixed with 4% paraformaldehyde for 30 min at 4 °C. Subsequently, 0.5% Triton X-100 was added and allowed to incubate for 10 min at room temperature. Following blocking with 10% goat serum for 1 h at room temperature, the cells were incubated overnight at 4 °C with or without the primary antibody (used as a negative control). After rinsing with PBS, the cells were then exposed to the secondary antibody for 1 h at room temperature in the dark, followed by blocking with an anti-fluorescence quenching solution (containing DAPI) (Beyotime, China). Images were captured using a fully automated inverted fluorescence microscope (Axio Observer7, Carl Zeiss, Germany).

### Immunohistochemistry

The hearts were sliced into 4 μm thick tissue sections, which were then dewaxed and underwent antigen retrieval. Subsequently, the tissue sections were treated with anti-MANF antibody (1:100, ab67271, Abcam, America) overnight at 4 °C, followed by incubation with HRP-coupled goat anti-rabbit IgG secondary antibody (1:10000, Sangon, China) for 1 h. After a 30-second hematoxylin re-staining, the sections were washed and stained with diaminobenzidine. The results were observed under a light microscope (Leica Microsystems, America) and captured in photographs.

### Tunel staining

Cell apoptosis was assessed using the Tunel BrightGreen Apoptosis Detection Kit (A112, Vazyme, China). Cells were fixed in 4% paraformaldehyde for 25 min at 4 °C, permeabilized with 0.2% Triton X-100, and then incubated with 50 µL of Tunel reaction mixture for 1 h at 37 °C under light-avoidance conditions. Subsequently, cells were blocked with anti-fluorescence quenching blocking solution (including DAPI) (Beyotime, China). Images were captured using a fully automated inverted fluorescence microscope (Axio Observer7, Carl Zeiss, Germany).

### Flow cytometry analysis

Samples were processed using the Annexin V-FITC/PI apoptosis detection kit following the provided instructions (A211, Vazyme, China). Negative controls were established and experimental groups were subjected to Annexin V-FITC and PI single staining for regulatory compensation. Flow cytometry analysis was performed on all samples using MoFlo XDP flow cytometer (Beckman) and FlowJo software (version 10.0).

### Echocardiography assays

To assess cardiac function in mice, M-mode echocardiograms were performed using an animal echocardiography system (VINNO 6 LAB, VINNO, China). The procedure involved removing chest hair with a depilatory instrument, applying a medical ultrasound coupler to the mice, and placing the probe next to the sternum to capture at least six consecutive cardiac cycles. Data, including measurements from three consecutive heartbeats, are presented in Supplementary Table S1.

### Evans Blue-2,3,5-triphenyltetrazolium chloride (TTC) staining

Before the end of reperfusion, use a syringe to inject 1–2 ml of 2% Evans Blue solution through the tail vein or the left ventricle of the heart. Heart samples were frozen and sliced into 1 mm thick sections. These sections were then incubated in TTC solution (Solarbio, G3005, China) at 37 °C for 15 min, followed by immediate termination of the reaction with 4% paraformaldehyde. The infarct area was identified through photography and analyzed using Image pro-plus (version 6.0) software. The percentage of infarct area was calculated by dividing the infarct area by the total area of the section.

### Masson staining

Paraffin sections were deparaffinized in distilled water and stained with Weigert’s iron hematoxylin staining solution. Acidic ethanol differentiation solution was used to differentiate the sections, which were then washed and stained with Ponceau S acid fuchsin stain solution. Following a 30-second wash in weak acid working solution, the sections were treated with phosphomolybdic acid solution for 1–2 min. Aniline blue staining solution was applied for 1–2 min after another weak acid wash. The sections were dehydrated with anhydrous ethanol, transparentized with xylene, and sealed with neutral gum. After overnight drying at room temperature, the slides were examined and photographed under a light microscope (Leica Microsystems, America).

### Enzyme-linked immunosorbent assay (ELISA)

Male mice were anesthetized and dissected 24 h after reperfusion. Blood was immediately collected from the heart, and then centrifuged at 1000 g for 20 min to obtain serum. The Lactate dehydrogenase (LDH) assay kit (A020-2-2) was acquired from Nanjing Jiancheng Bioengineering Research Institute. Additionally, mouse cardiac troponin I (cTnI) ELISA kit (JL11280), mouse creatine kinase MB isoform (CKMB) ELISA kit (JL12422), mouse interleukin 6 (IL-6) ELISA kit (JL20268), mouse interleukin 1Beta (IL-1β) ELISA kit (JL18442), and mouse tumor necrosis factor alpha (TNF α) ELISA kit (JL10484) were purchased from Shanghai Jianglai Biotechnology Co., Ltd. The indicators in mouse serum were detected following the instructions of the respective reagent kits, and the final optical density was measured at 450 nm.

### Statistical analysis

Statistical analysis was conducted using GraphPad Prism 10 (GraphPad Software, America). Each experiment was replicated a minimum of three times, and results are presented as mean ± standard deviation. Student’s t-test was utilized for comparing two groups with normally distributed data, while One-way analysis of variance (ANOVA) followed by Tukey’s post-hoc test was employed for comparing multiple groups. A significance level of *P* < 0.05 was used to determine statistical significance.

## Results

### Myocardial I/R injury increases the expression and secretion of MANF

**Fig. 1:**
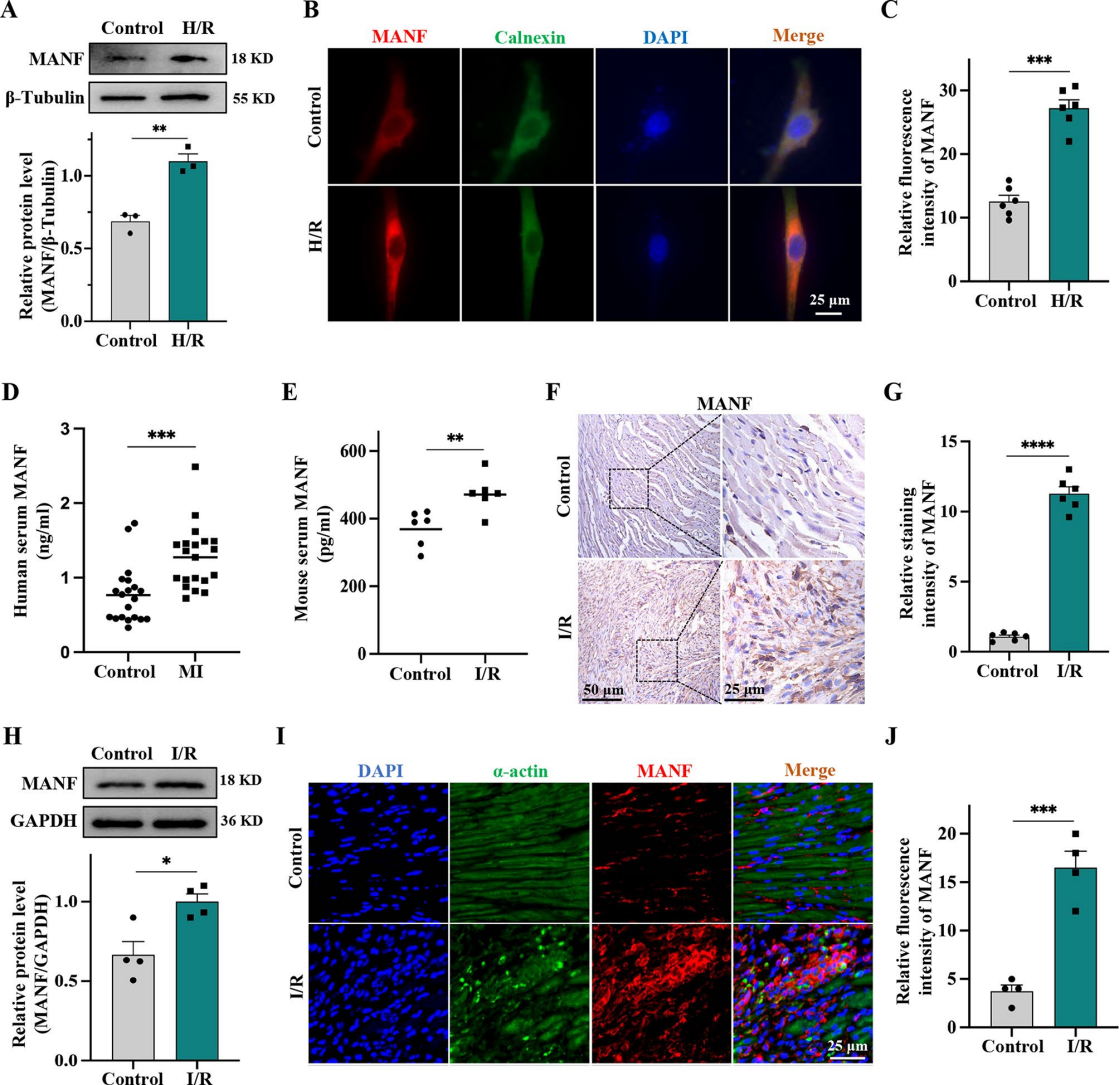
I/R increases the expression and secretion of MANF in the heart. **A** Western blot analysis was used to determine MANF levels in HL-1 treated with H/R, with quantitative bands normalized to β-Tubulin. **B-C** Representative immunofluorescence staining and statistical quantification of MANF (red), Calnexin (green) and DAPI (blue) in HL-1 subjected to H/R treatment, with Calnexin labeling the ER. Scale bar, 25 μm. **D** Serum levels of MANF in MI patient (*n* = 20) and healthy control (*n* = 20). **E** Serum levels of MANF in I/R mice (*n* = 6) and normal mice (*n* = 6). **F-G** Representative immunohistochemical images and statistical quantification of MANF expression in I/R mice and normal mice using anti-MANF antibody. Scale bar, 25 μm. **H** Western blot analysis and quantification of MANF levels in I/R mice. **I-J** Representative immunofluorescence staining and statistical quantification of MANF (red), α-actin (green) and DAPI (blue) in I/R mice, with α-actin marking actin fibers. Scale bar, 25 μm. Data are presented as mean ± SD and represent at least 3 independent experiments. **P<0.01, ***P<0.001, ****P<0.0001

An in vitro cardiomyocyte H/R model was utilized to mimic myocardial I/R injury. The survival rate of HL-1 exhibited a time-dependent decrease following H/R stimulation, leading to the selection of 20 h of hypoxia (65% survival rate) followed by 3 h of reoxygenation as the H/R time frame for this study (Supplementary Fig. S1). Subsequent to H/R stimulation, there was an increase in the expression and secretion of MANF compared to the control group (Fig. 1A). Immunofluorescence analysis revealed that MANF was primarily localized in the ER and its expression level was elevated (2-fold) post H/R stimulation. Moreover, it was observed that H/R alone did not induce significant changes in the morphology of cardiomyocytes (Fig. 1B, C). To further investigate the role of MANF in myocardial I/R injury, a mouse model of I/R injury was established. Assessment of MANF release during I/R injury was conducted by evaluating serum levels of MANF expression in MI patients and I/R-injured mice. The findings indicated an upregulation of MANF in the serum of both MI patients and I/R-injured mice (Fig. 1D, E). Immunohistochemical analysis further validated the significant elevation of MANF following myocardial I/R injury in mice (Fig. 1F, G). Similarly, western blot analysis revealed increased MANF expression in the hearts of I/R mice (Fig. 1H). Tissue immunofluorescence co-localization analysis of MANF in myocardial tissue revealed that MANF is predominantly located within cardiomyocytes. In I/R mice, MANF levels are significantly increased, primarily distributed around the nucleus. Actin fibers typically exhibit a uniformly distributed fibrous structure in normal mice. However, actin fibers may seem fragmented, disorganized, or rearranged in I/R mice as a result of myocardial cell injury (Fig. 1I, J). Collectively, these results suggest that myocardial I/R injury leads to an increase in the expression and secretion of MANF.

### MANF reduces cardiomyocyte apoptosis in myocardial I/R injury

**Fig. 2:**
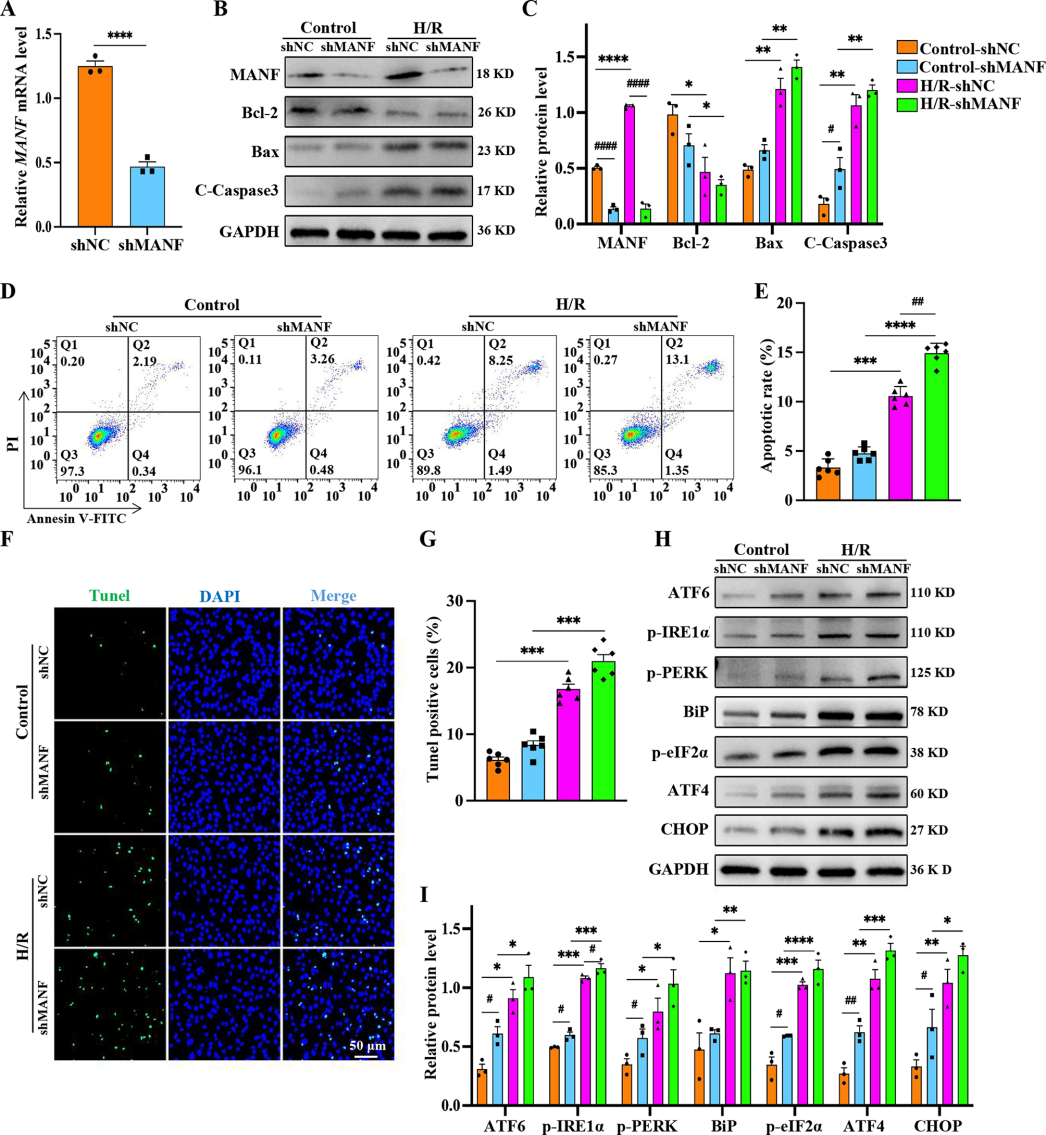
shMANF increases H/R-induced apoptosis and ER stress. HL-1 was divided into shNC (control) and shMANF (MANF knockout) groups and subjected to H/R stimulation. **A** Relative mRNA levels of MANF was determined using qPCR. **B-C** Western blot analysis and statistical quantification of MANF levels, as well as levels of apoptosis-related proteins (cleaved caspase3, Bcl-2, Bax). The quantitative bands were normalized to GAPDH. **D-E** Representative flow cytometry plots and statistical quantification of apoptotic cells. **F-G** Representative Tunel fluorescence images and statistical quantification of Tunel-positive apoptotic cells. Scale bar, 50 μm. **H-I** Western blot analysis and statistical quantification of the UPR-related protein levels (ATF6, p-PERK, p-IRE1α, BiP, p-eIF2α, CHOP, ATF4). The quantitative bands were normalized to GAPDH. Data are presented as mean ± SD and represent at least 3 independent experiments. Control vs. H/R, *P<0.05, **P<0.01, ***P<0.001, ****P<0.0001; shNA vs. shMANF, ^**#**^*P*<0.05, ^**##**^*P*<0.01, ^**###**^*P*<0.001, ^**####**^*P*<0.0001

To investigate the role of MANF during I/R injury, a stably transfected cell line with lentiviral vector targeting the MANF gene (shMANF) was constructed. The qPCR results showed that the expression of shMANF was reduced by nearly 70% (Fig. 2A), which was further confirmed by western blot analysis (Fig. 2B). Assessment of apoptotic markers revealed decreased levels of anti-apoptotic protein Bcl-2 and increased levels of pro-apoptotic proteins Bax and cleaved caspase-3 in shMANF cells following H/R (Fig. 2B, C). Flow cytometry data supported the exacerbation of cardiomyocyte apoptosis by shMANF (Fig. 2D, E), along with an increase in Tunel-positive cells (Fig. 2F, G and Supplementary Fig. S2). Interestingly, even under physiological conditions, shMANF displayed higher levels of cell apoptosis and Tunel-positive cells compared to shNC, underscoring the critical role of MANF in cardiomyocyte survival.

**Fig. 3:**
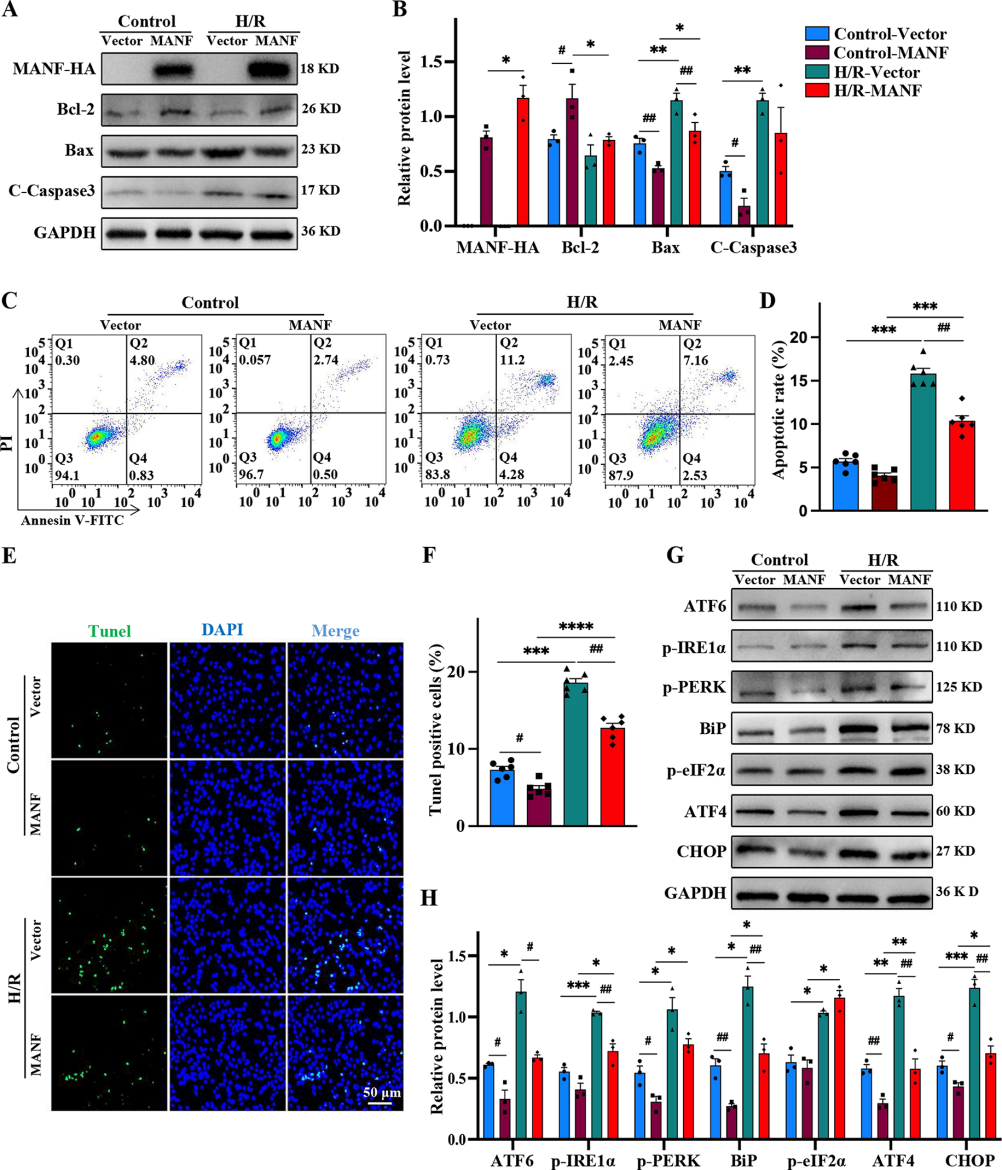
MANF alleviates ER stress‑induced apoptosis subjected to H/R. HL-1 was transfected with Vector (control group) and MANF (MANF overexpression group), followed by H/R stimulation. **A-B** Western blot analysis and statistical quantification were performed to assess the levels of apoptosis-related protein (cleaved caspase3, Bcl-2, Bax), with normalization to GAPDH. **C-D** Representative flow cytometry plots and statistical quantification of all apoptotic cells. **E-F** Representative Tunel fluorescence images and statistical quantification of Tunel-positive apoptotic cells. Scale bar, 50 μm. **G-H** Western blot analysis and statistical quantification of the UPR-related protein levels (ATF6, p-PERK, p-IRE1α, BiP, p-eIF2α, CHOP, ATF4), with normalization to GAPDH. Data are presented as mean ± SD and represent at least 3 independent experiments. Control and H/R, *P<0.05, **P<0.01, ***P<0.001, ****P<0.0001; Vector and MANF, ^**#**^*P*<0.05, ^**##**^*P*<0.01, ^**###**^*P*<0.001, ^**####**^*P*<0.0001

Subsequent experiments involving transient transfection of MANF overexpression plasmid (MANF-HA) into HL-1 demonstrated a protective effect against H/R-induced injury. MANF overexpression led to increased Bcl-2 expression, decreased levels of Bax and cleaved caspase-3, and reduced apoptosis compared to control vector-transfected cells (Fig. 3A, B). Flow cytometry analysis further supported the anti-apoptotic effect of MANF overexpression, highlighting its role in protecting cells from H/R injury (Fig. 3C, D). Similarly, MANF overexpression reduced H/R-induced apoptosis and the number of Tunel-positive cells (Fig. 3E, F). In conclusion, MANF overexpression appears to confer protection against H/R-induced cardiomyocyte injury by mitigating apoptosis.

### MANF protects cells from H/R injury by attenuating ER stress

Previous studies have demonstrated that MANF protects cells from ER stress-induced apoptosis by regulating UPR-related genes in ischemic models (Yang et al. 2021). To investigate whether MANF mitigates H/R-induced cardiomyocyte apoptosis by alleviating ER stress, the expression levels of proteins associated with the three pathways of ER stress were analyzed in MANF knockout and overexpression systems. The expression of ER stress marker BiP was upregulated post H/R stimulation in shMANF, indicating the presence of cellular ER stress. Conversely, the expression of BiP decreased after MANF overexpression, affirming its capability to alleviate ER stress. The levels of ER stress pathway proteins ATF6, p-PERK, and p-IRE1α escalated following H/R stimulation, predominantly involving the PERK/eIF2α/ATF4/CHOP pathway (Fig. 2H, I). Conversely, MANF overexpression led to a reduction in the expression of ER stress-related proteins induced by H/R. Moreover, diminished expression levels of ER stress-related proteins were observed in MANF overexpressing cells even in the absence of H/R, suggesting that MANF maintains cellular homeostasis under normal conditions by alleviating ER stress (Fig. 3G, H). Therefore, MANF serves to safeguard cardiomyocytes from H/R injury and diminish apoptosis by alleviating ER stress.

### rMANF improves cardiac function in mice by attenuating myocardial I/R injury

**Fig. 4:**
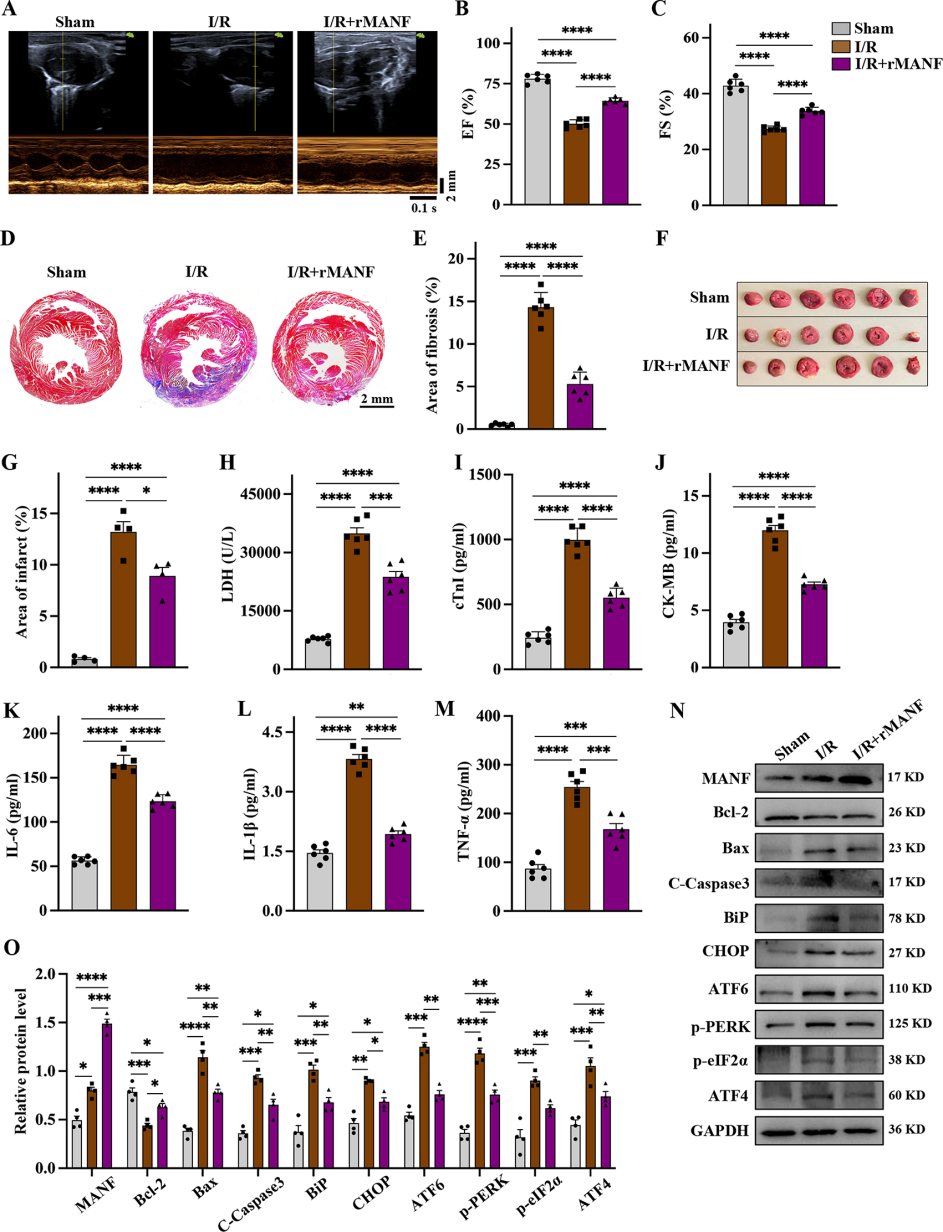
rMANF protein improves heart function and reduces inflammation. The mice were categorized into Sham group, I/R group and ischemic with simultaneous injection of rMANF (1.5 mg/kg) followed by reperfusion group. **A-C** Representative images of M-mode echocardiography and statistical quantitative analysis of EF and FS. **D-E** Representative images of Masson staining of the whole heart and statistical quantitative the area of fibrosis. Collagen fibers were stained blue. Scale bar, 2 mm. **F-G** Representative cross-sectional images of TTC staining and statistical quantitative the area of infarct. Each heart slice cut into 6 pieces. **H** Detection of serum LDH in different groups of mice using the microplate method. **I-M** ELISA kits for the determination of cTnI, CK-MB, IL-6 and IL-1β levels in mice serum. **N-O** Western blot analysis and statistical quantification of the apoptosis-related protein (cleaved caspase3, Bcl-2, Bax) and the UPR-related protein levels (BiP, CHOP, ATF6, p-PERK, p-eIF2α, ATF4), with normalization to GAPDH. Data are presented as mean ± SD, *n* = 6 mice per group, 3 independent experiments. Sham vs. I/R, Sham vs. I/R + rMANF, I/R vs. I/R + rMANF, **P<0.01, ***P<0.001, ****P<0.0001

rMANF has been shown to enhance cell viability and inhibit ER stress-induced apoptosis in non-neuronal cells (Yang et al. 2021; Zhao et al. 2024). To investigate the pathophysiological role of MANF in myocardial I/R injury, the effects of rMANF were examined in I/R-injured mice. Echocardiography revealed that injection of rMANF significantly improved cardiac function in the presence of myocardial I/R injury, as demonstrated by increased ejection fraction and fractional shortening (Fig. 4A-C and Supplementary Table S1). We also observed that injecting rMANF during ischemia was more effective than during reperfusion (Supplementary Fig. S3). Moreover, injection of rMANF led to a reduction in collagen fibers in the infarct area, as shown by Masson staining analysis and Sirius Red staining (Fig. 4D, E and Supplementary Fig. S4). The results of TTC staining (Fig. 4F, G) and Evans Blue-TTC double staining (Supplementary Fig. S5). both indicated that the myocardial infarction area in I/R mice was reduced by approximately 40% after rMANF treatment. Furthermore, levels of LDH, cTnI and CK-MB, which are biochemical markers of myocardial injury, were significantly reduced in mice treated with rMANF during myocardial I/R injury (Fig. 4H-J). Additionally, rMANF treatment decreased the levels of inflammatory factors IL-1β, IL-6, and TNF-α that are typically elevated in response to I/R injury (Fig. 4K-M). The expression of apoptosis and ER stress-related proteins were also detected by western blot in vivo. Compared with the sham group, it was found that the expression of the apoptosis marker protein Bcl-2 was reduced and the levels of Bax and cleaved caspase-3 were increased in the I/R group. Meanwhile, the levels of ER stress marker proteins BiP, CHOP and ER stress pathway proteins ATF6, p-PERK, p-eIF2α and ATF4 increased after I/R stimulation. In contrast, the addition of rMANF reduced I/R-induced ER apoptosis and the expression of stress-related proteins (Fig. 4N, O). Studies have found that autophagy dysfunction plays a key role in the process of myocardial I/R injury (Xing et al. 2022; Kim et al. 2023). Western blot analysis of the autophagy molecular marker proteins LC3B and SQSTM1 found that LC3B increased and SQSTM1 decreased after I/R, indicating that the autophagy flow was blocked after I/R, and the blocked autophagic flow was restored after adding rMANF (Supplementary Fig. S6). The above results indicate that MANF plays an important role in the I/R process through autophagy. These findings suggest that rMANF effectively mitigates cardiac dysfunction induced by myocardial I/R injury.

### Key subdomains regulating expression and secretion of MANF

**Fig. 5:**
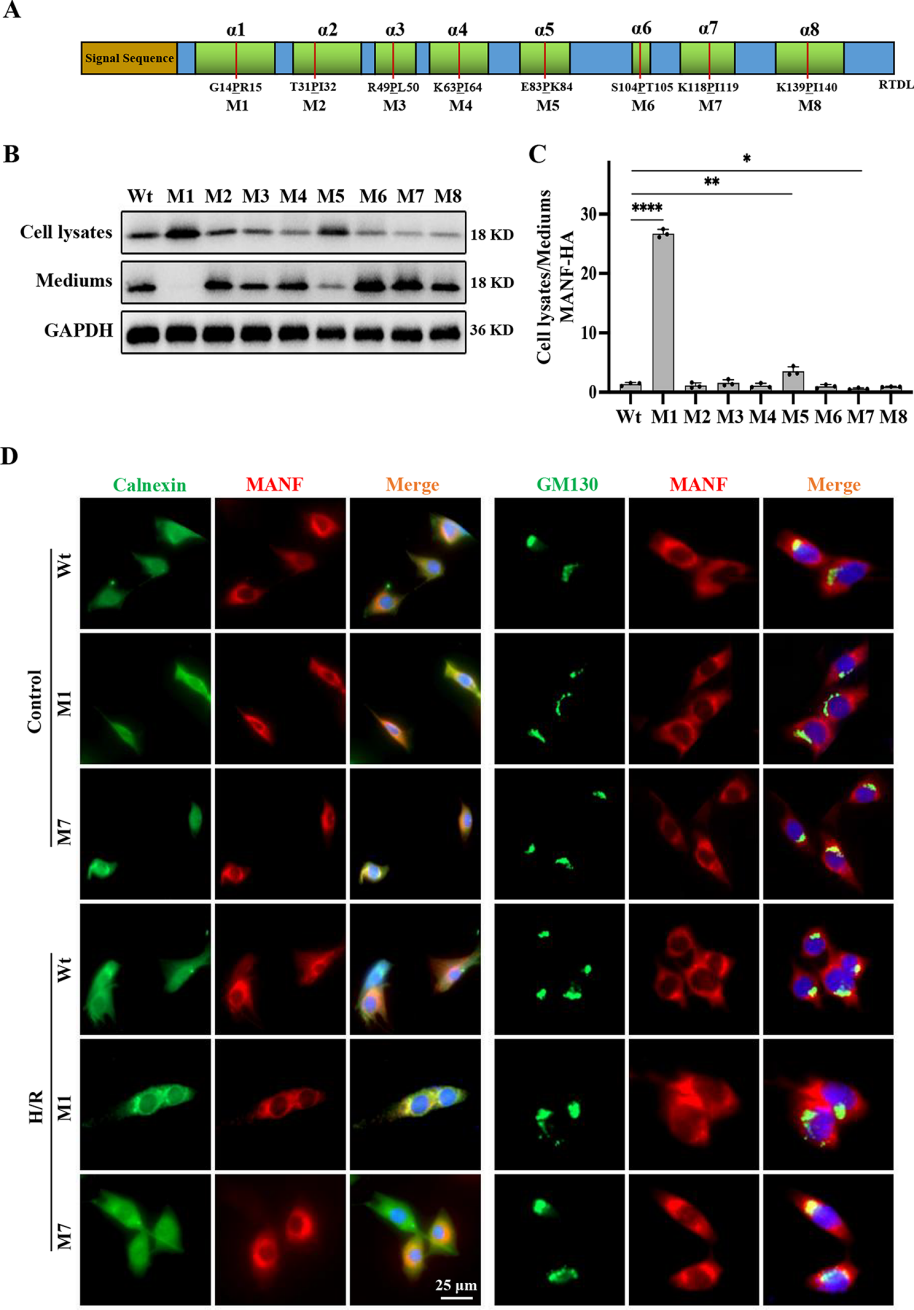
Screening of α-helix regulating MANF expression and secretion. **A** Schematic diagram of the amino acid sequence of MANF, showing the eight α-helices in green boxes. Mutants M1 to M8 were generated by proline insertion into the respective α-helices, with mutation sites marked by red lines. **B** HL-1 were transfected with Wt (wild-type MANF)and mutants followed by analysis of cell lysates and culture medium using immunoblotting with anti-HA antibody. **C** Quantitative data is presented as cell lysate/medium ratios, with results shown as mean ± SD. Statistical significance is denoted as *P<0.05, **P<0.01, ****P<0.0001. **D** Representative immunofluorescence staining of MANF (red) co-localization with Calnexin (ER, green), GM130 (Golgi, green) and DAPI (blue, not shown) in H/R-treated HL-1. Scale bar, 25 μm. Data are presented as mean ± SD and represent at least 3 independent experiments

Considering the unique protein folding conformation of MANF, a series of MANF mutants (M1-M8) were constructed by inserting proline into the corresponding α-helix to investigate the impact of the α-helix on the secretion and function of MANF (Fig. 5A). Western blot analysis following the overexpression of these mutants revealed that two crucial α-helices (α1 and α7) in the secondary structure of MANF influenced the intracellular transportation and secretion of MANF. Specifically, M1 (α1 mutant) exhibited predominantly intracellular expression and lacked extracellular secretion, whereas M7 (α7 mutant) displayed reduced intracellular expression but increased secretion levels compared to Wt (wild type) (Fig. 5B, C). To further explore the intracellular expression and co-localization of M1 and M7, their co-localization with ER-tagged protein (Calnexin) and Golgi-tagged protein (GM130) was examined. Immunofluorescence analysis indicated that M1 primarily resided in the ER and did not reach the Golgi apparatus, while M7 showed decreased presence in the ER and was predominantly transported to the Golgi apparatus, consistent with the western blot findings (Fig. 5D). These findings suggest that the two key subdomains (α1 and α7) influence the expression and secretion of MANF intracellularly, potentially impacting the role of MANF and its mutants in myocardial I/R injury.

### M1 is more protective against myocardial H/R injury

**Fig. 6:**
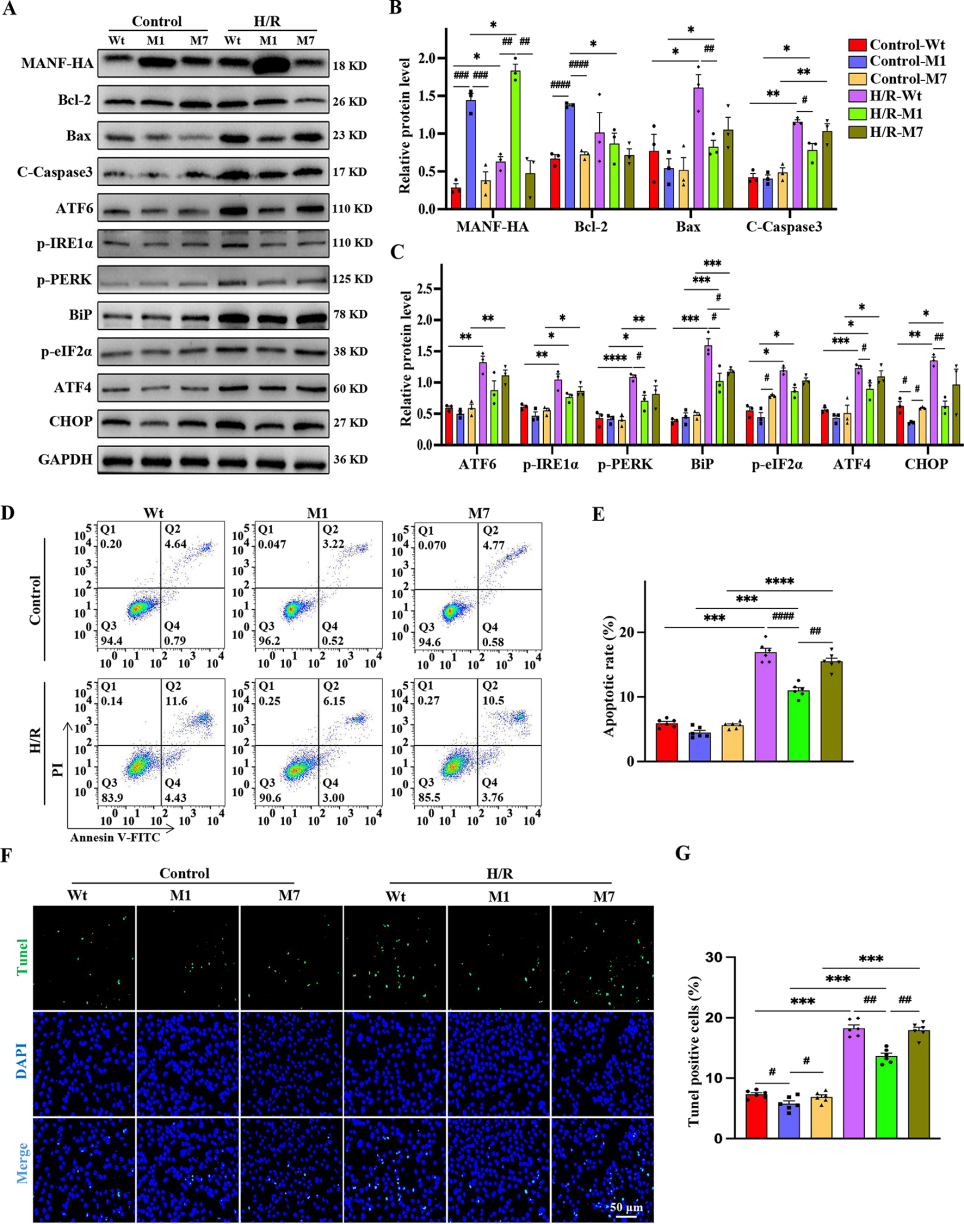
M1 reduces apoptosis and ER stress to a greater extent than Wt and M7. HL-1 was transfected with Wt, M1 and M7 respectively, followed by H/R stimulation. **A-C** Western blot analysis and statistical quantification of MANF, apoptosis-related protein levels (cleaved caspase3, Bcl-2, Bax) and UPR-related proteins (ATF6, p-PERK, p-IRE1α, BiP, p-eIF2α, CHOP, ATF4). The quantitative bands were normalized to GAPDH. **D-E** Representative flow cytometry plots and statistical quantification of all apoptotic cells. **F-G** Representative Tunel fluorescence images and statistical quantification of Tunel-positive apoptotic cells. Scale bar, 50 μm. Data are presented as mean ± SD and represent at least 3 independent experiments. Control vs. H/R, *P<0.05, **P<0.01, ***P<0.001, ****P<0.0001; Wt vs. M1, Wt vs. M7, M1 vs. M7, ^**#**^*P*<0.05, ^**##**^*P*<0.01, ^**###**^*P*<0.001, ^**####**^*P*<0.0001

To investigate the protective effects of MANF mutants against H/R-induced injury, M1 and M7 were overexpressed to assess the impact on apoptosis and UPR-related proteins. Western blot analysis demonstrated that M1 had significantly higher intracellular expression levels compared to M7 and Wt. Moreover, M1 upregulated Bcl-2 expression and downregulated Bax and cleaved caspase-3 levels (Fig. 6A, B). UPR-related protein analysis showed that M1 reduced ATF6, p-PERK, p-IRE1α, BiP, p-eIF2α, CHOP, and ATF4 expression levels compared to Wt and M7, indicating a further alleviation of ER stress by M1 during H/R. These findings suggest that M1 provides stronger protection against cardiomyocyte injury (Fig. 6A-C). Flow cytometric assays confirmed M1’s superior ability to reduce apoptosis in cardiomyocytes (Fig. 6D, E). Additionally, M1 decreased the number of Tunel-positive cells compared to Wt and M7 (Fig. 6F, G). Overall, our results indicate that mutation of the α1-helix not only impacts MANF’s structure and localization, but also enhances its cytoprotective function under stress conditions.

### M1 protect cardiomyocytes from H/R injury through the JAK1/STAT1/NF-kB signaling pathway

**Fig. 7:**
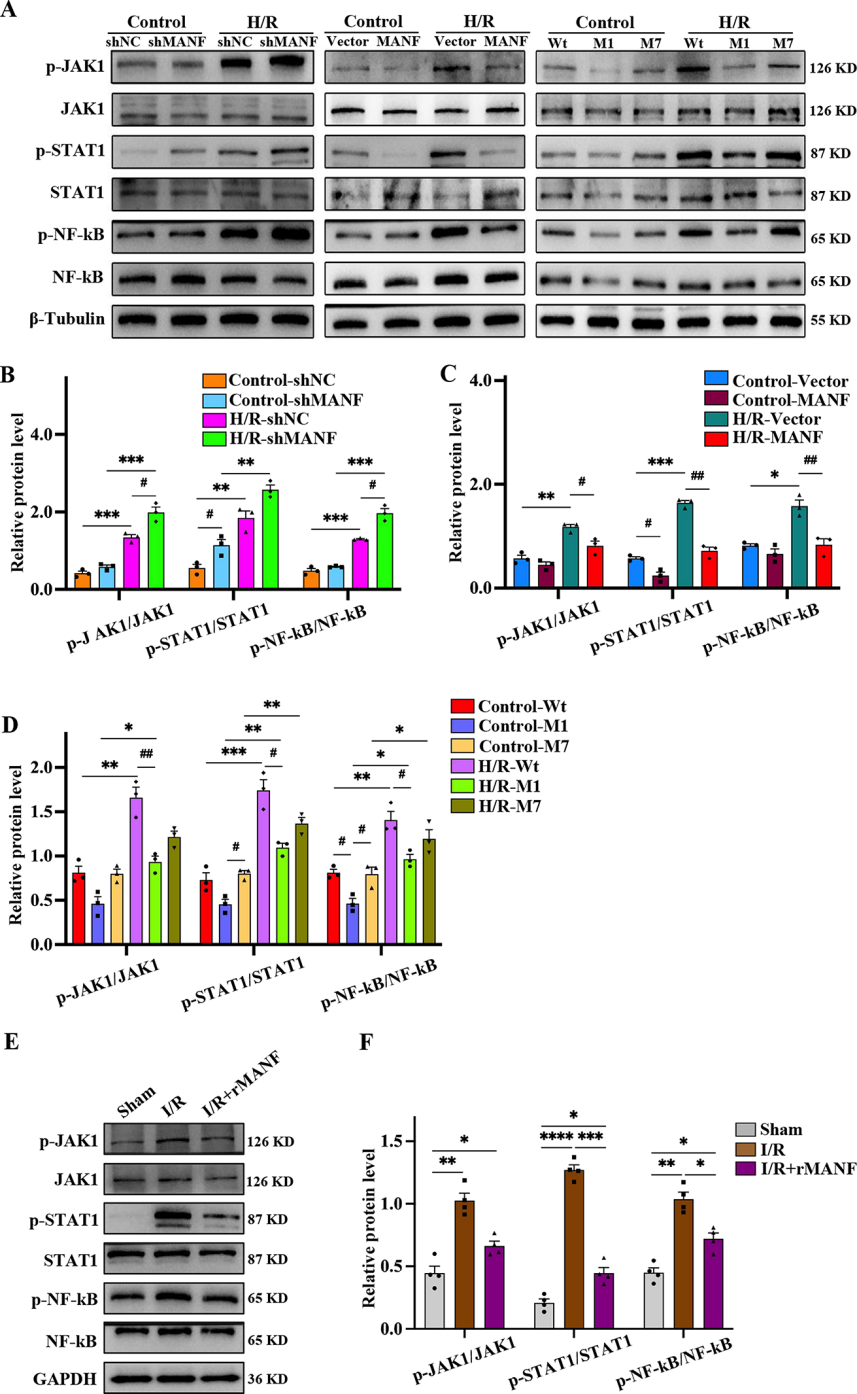
M1 demonstrated greater protective effects compared to Wt and M7 via the JAK1/STAT1/NF-κB signaling pathway. **A-D** Western blot analysis and statistical quantification of p-JAK1/JAK1, p-STAT1/STAT1, p-NF-κB/NF-κB protein levels in HL-1 exposed to H/R after manipulation of MANF expression (knockout, overexpression of Wt, M1, and M7) were performed. The quantitative bands were normalized to β-Tubulin (*n* = 3). **E-F** Western blot analysis and statistical quantification of p-JAK1/JAK1, p-STAT1/STAT1, p-NF-κB/NF-κB protein levels in I/R mice added with rMANF. The quantitative bands were normalized to GAPDH (*n* = 4). Data are presented as mean ± SD and represent at least 3 independent experiments. Control vs. H/R, *P<0.05, **P<0.01, ***P<0.001; shNA vs. shMANF, Vector vs. MANF, Wt vs. M1, Wt vs. M7, M1 vs. M7, ^**#**^*P*<0.05, ^**##**^*P*<0.01, ^**###**^*P*<0.001

The JAK/STAT signaling pathway, consisting mainly of Janus tyrosine kinases (JAK) and signal transducers and activators of transcription (STAT), plays a crucial role in I/R injury (Xu et al. 2020). Activation of JAK1/STAT1 has been linked to inflammatory responses and apoptosis in cardiac tissue injury (Du et al. 2020). In this study, we investigated the JAK1/STAT1 signaling pathway to elucidate how MANF reduces myocardial injury. Western blot analysis was conducted to assess the levels of p-JAK1/JAK1 and p-STAT1/STAT1 proteins. The results revealed that both shMANF and H/R increased the expression of p-JAK1 and p-STAT1, while MANF overexpression led to a decrease in their expression (Fig. 7A-C). M1 showed a greater reduction in p-JAK1/JAK1 and p-STAT1/STAT1 levels compared to Wt and M7, aligning with its effectiveness in attenuating ER stress and reducing apoptosis (Fig. 7A, D). Additionally, we examined NF-κB to investigate its involvement in the inflammatory response and apoptosis post H/R injury. The findings indicated that p-NF-κB levels significantly rose after shMANF and H/R injury, but MANF overexpression reduced these levels. Similarly, M1 exhibited a greater reduction in p-NF-κB/NF-κB expression compared to Wt and M7 (Fig. 7A-D). The expression levels of p-JAK1/JAK1, p-STAT1/STAT1 and p-NF-κB/NF-κB were also detected in vivo, and it was found that addition of rMANF can effectively inhibit the activation of the JAK1/STAT1/NF-κB signaling pathway (Fig. 7E, F). This Consistent with results found in vitro. Overall, our study suggests that MANF can modulate cardiomyocyte apoptosis through the JAK1/STAT1/NF-kB pathway, highlighting its potential as a mechanism for anti-apoptosis following myocardial I/R injury.

**Fig. 8:**
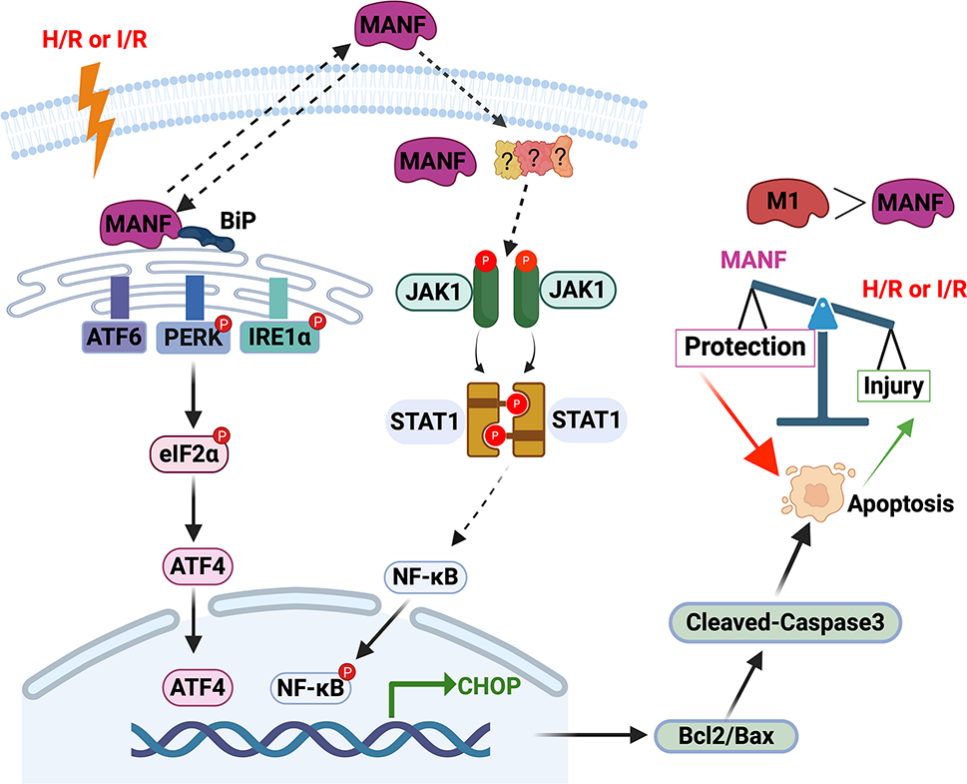
Schematic diagram of the regulatory role of MANF on I/R-induced apoptosis. Cell injury induced by H/R or I/R triggers ER stress and activates the UPR, subsequently leading to the activation of the PERK/eIF2α/ATF4/CHOP signaling pathway, ultimately promoting apoptosis in cardiomyocytes. MANF functions by inhibiting ER stress and the JAK1/STAT1/NF-κB signaling pathway, thereby exerting a protective effect on the cells either directly or indirectly. Additionally, it is noted that the protective effect of M1 on myocardial injury appears to be stronger than that of MANF

## Discussion

Myocardial I/R injury is a severe and potentially fatal condition that is a leading cause of mortality worldwide. Our research indicated elevated levels of MANF protein in both MI patients and mice experiencing I/R injury, highlighting the significant role of MANF in this pathological process. Overexpression of MANF in cardiomyocytes was found to decrease ER stress-induced cell death, whereas MANF depletion exacerbated this effect. Treatment with rMANF was shown to enhance cardiac function in mice with I/R injury by reducing infarct area and inflammation. Additionally, our study suggested that alterations in the α1-helix region of MANF protein might impact its structure, expression, secretion and overall function. Notably, M1 variant of MANF exhibited superior effects in alleviating ER stress and decreasing apoptosis compared to Wt and M7 variants. Mechanistically, MANF exerts its protective effects against myocardial I/R injury primarily by alleviating ER stress-induced apoptosis and modulating the JAK1/STAT1/NF-κB signaling pathway.

Several studies have demonstrated that MANF expression is increased in various pathological conditions associated with ER stress, including multiple myeloma, glomerular disease, rheumatoid arthritis, hepatic damage and systemic lupus erythematosus (Tousson-Abouelazm et al. 2020; Dernoncourt et al. 2021). Similarly, the release of MANF was heightened after ER stress induced by ischemia. In our investigation, we observed elevated levels of MANF in HL-1 following stimulation with H/R. Additionally, levels of MANF in the serum were found to be elevated in MI patients and I/R mice. Administration of rMANF before ischemia improved myocardial function and reduced infarct area to some extent, highlighting the protective role of MANF as an ER stress-induced secreted cardiomyokine. Current research generally supports the idea that MANF helps alleviate ER stress-induced apoptosis.

The α-helix is a prevalent secondary structure in proteins, crucial for their stability and overall structure (Guarracino et al. 2019). Mutations can disrupt this helical structure, hindering proper protein folding and maintenance of natural conformation. Conversely, mutations can sometimes lead to new protein functions by altering the protein’s structure to interact with different molecules or participate in novel biological processes. In our research, we introduced proline into the α-helix of MANF to modify its spatial structure and examined how this affected the protein’s intracellular expression and secretion dynamics (Liu et al. 2023a, b). Our findings indicated that the M1 mutant variant showed increased expression in ER due to the α-helix mutation, while Wt and M7 variants were secreted extracellularly via the Golgi apparatus. This highlights how mutations in the α1-helix can impact the subcellular localization and secretion pathways of MANF proteins. Furthermore, we observed that the M1 mutant provided greater protection against myocardial injury during H/R conditions. It was found that MANF can exert its cytoprotective effects both intracellularly and extracellularly. The results suggest that retaining M1 in the intracellular ER following the α1-helix mutation enhances MANF’s cytoprotective function under stress conditions, indicating that α1-helix mutations not only influence MANF’s structure and localization but also bolster its protective capabilities.

Under ischemic and hypoxic conditions, intrinsic cellular mechanisms respond to ER stress by activating UPR signaling pathway (Kovaleva et al. 2023). This involves three ER transmembrane protein receptors: IRE1α, PERK and ATF6. PERK, with its kinase activity, phosphorylates eIF2α, leading to a selective inhibition of overall protein synthesis and retention of the transcription factor ATF4. ATF4 then translocate to the nucleus to regulate the transcription of UPR-related genes. Additionally, PERK activation induces CHOP-mediated apoptosis (Read and Schröder 2021). Our findings indicate that overexpression of MANF resulted in decreased levels of UPR-related proteins such as ATF6, p-PERK, p-IRE1α, BiP, p-eIF2α, CHOP and ATF4 in cells. The ability of M1 to reduce the expression of these proteins was more pronounced compared to Wt and M7, suggesting that MANF and M1 mitigate apoptosis by inhibiting UPR signaling activation and alleviating H/R-induced ER stress. Furthermore, our data revealed that all three UPR sensors are activated to some extent by H/R, indicating a potential interconnection between the UPR branches in the context of cardiac H/R injury. MANF and M1 were found to exert a protective effect against H/R-induced cell injury by reducing apoptosis through ERS mitigation. Notably, the mutation of the α-helix structure in M1 enhanced its ability to counteract oxidative stress, warranting further investigation into the precise mechanism by which MANF modulates cellular stress responses.

The primary mechanism through which MANF protects the heart from I/R injury is by attenuating ER stress, but there are additional mechanisms at play (Meyer and Doroudgar 2020; Arrieta et al. 2020). Research has shown that the JAK1/STAT1 signaling pathway is implicated in apoptosis development (Zhu et al. 2017; Caglayan et al. 2022). Cytokine binding to receptors triggers receptor dimerization, leading to JAK activation and subsequent phosphorylation of STAT family members. This cascade is crucial in stress signaling pathways governing gene expression and cell death. Studies have indicated that the JAK1/STAT1 signaling pathway modulates inflammation and left ventricular remodeling post-myocardial infarction, thereby mitigating myocardial I/R injury in cardiac tissues and cardiomyocytes (Zhang et al. 2023a, b). Researchers have observed that this pathway can reduce cardiac hypertrophy by enhancing autophagy and inhibiting mitochondria-dependent apoptosis (Liu et al. 2023a, b). These findings suggest a pivotal role of the JAK1/STAT1 pathway in regulating cellular inflammation and apoptosis during I/R injury. In our study, in vitro experiments verified that H/R significantly increased the levels of p-JAK1 and p-STAT1, while overexpression of MANF and M1 reduced their expression in myocardial I/R. Similar to its ER stress attenuation ability, M1 showed a more pronounced decrease in p-JAK1 and p-STAT1 levels compared to Wt and M7. In vivo experiments similarly concluded that rMANF effectively reduces p-JAK1 and p-STAT1 levels. The consistency between in vivo and in vitro experimental results enhances the credibility of our conclusions. We acknowledge that despite providing important mechanistic insights, the limitations of in vitro models cannot be ignored. Future research should integrate more in vivo and in vitro experiments, particularly through more complex animal models and clinical samples, to comprehensively understand the role of MANF in I/R injury.

Crystal structure analysis revealed that the C-terminal structural domain of MANF is similar to Ku 70, an anti-apoptotic protein that interacts with pro-apoptotic Bax and is homologous to its SAP structural domain. SAP-like structural domains have been shown to promote MANF interaction with p65 and negatively regulate NF-κB signaling under inflammation and ER stress (Chen et al. 2015). While only one study has revealed the interaction of secreted MANF and its receptor NTPN, which regulates inflammation through the NF-κB pathway (Yagi et al. 2020). The current results suggest that MANF can inhibit the JAK1/STAT1/NF-κB signaling pathway, but further research is needed to explore potential additional connections between MANF and JAK1/STAT1/NF-κB signaling. These findings emphasize the complex mechanism by which MANF confers cardioprotection, involving not only the inhibition of UPR signaling but also the modulation of the JAK1/STAT1/NF-κB signaling pathway. These discoveries offer valuable insights into the role of MANF in heart disease and provide important information for the development of novel strategies for heart disease treatment in the future.

Reperfusion or the rapid restoration of blood flow is essential for myocardial survival but can also lead to myocardial injury and ER stress. Research has shown that injecting rMANF can reduce I/R injury and identifying a key motif in MANF that regulates its secretion and transport suggests it could be a potential therapeutic target. Screening for medications that modulate MANF may offer treatment options for ER stress-related diseases. Further studies, such as intracardiac injections or using genetically modified animal models with MANF overexpression, could make MANF a valuable therapeutic target. However, it is important to note that the pharmacokinetics and pharmacodynamics of rMANF are not well-defined, and issues like instability and inactivation are common with recombinant proteins. Improvements are necessary to enhance the pharmacokinetic and pharmacodynamic properties of MANF for better efficacy in clinical applications, paving the way for more effective treatment strategies for ER stress-related diseases.

## Conclusion

This study highlights the significant role of MANF and its subdomains in mitigating cardiomyocyte apoptosis induced by I/R through the reduction of ER stress and the inhibition of JAK1/STAT1/NF-κB activation (Fig. 8). These findings contribute to a better understanding of MANF’s function in cardiology. MANF and its α-helix mutants emerge as crucial regulators in myocardial I/R injury and promising targets for the treatment of I/R-related diseases.

## Electronic supplementary material

Below is the link to the electronic supplementary material.

Supplementary Material 1

Supplementary Material 2

## Data Availability

The data supporting the findings of this study are found in the article and the supplementary material.
