# Supplementary material for: Key subdomains of mesencephalic astrocyte-derived neurotrophic factor attenuate myocardial ischemia/reperfusion injury by JAK1/STAT1/NF-κB signaling pathway
Source: Mol Med. 2024 Sep 6;30:139. doi: 10.1186/s10020-024-00916-6 (PMC11380330; doi:10.1186/s10020-024-00916-6)

Supplementary Original western blots

Figure 1 A

MANF

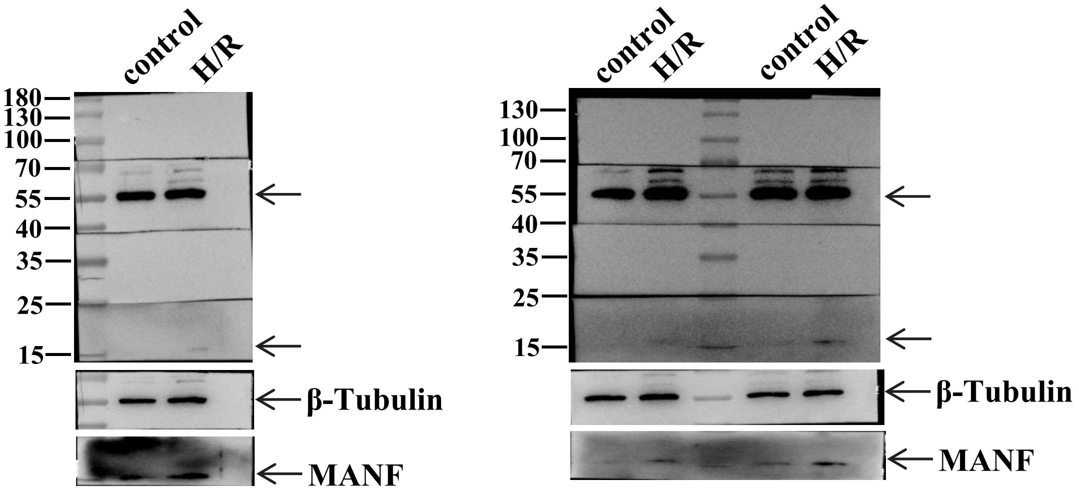

Figure 1 H

MANF

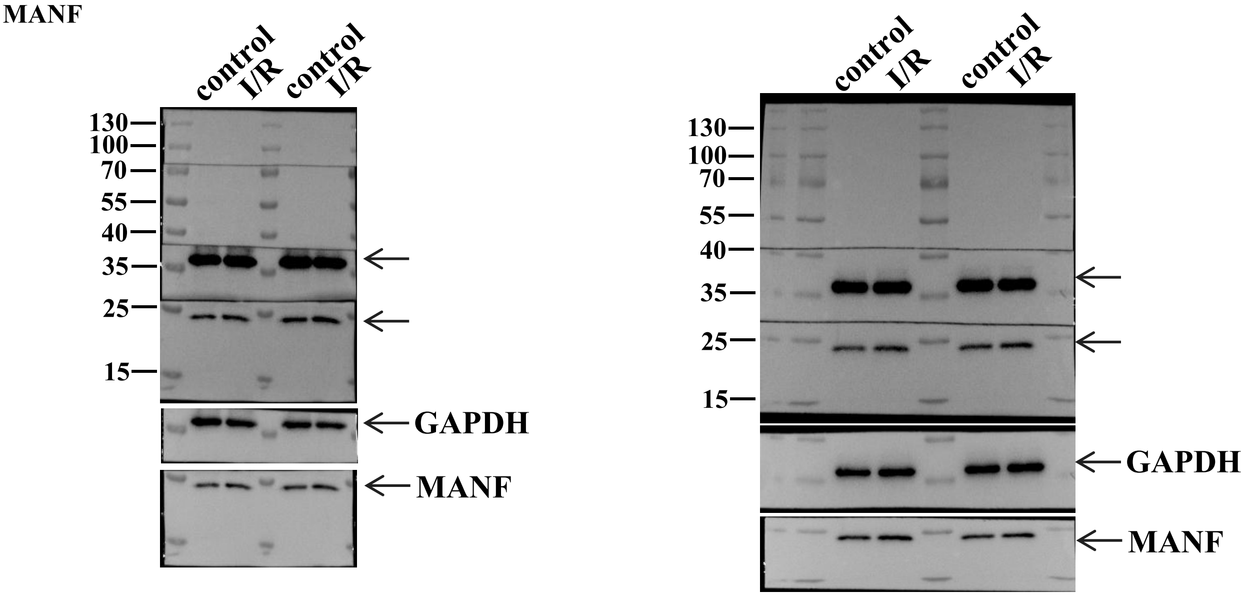

Figure 2 B

MANF

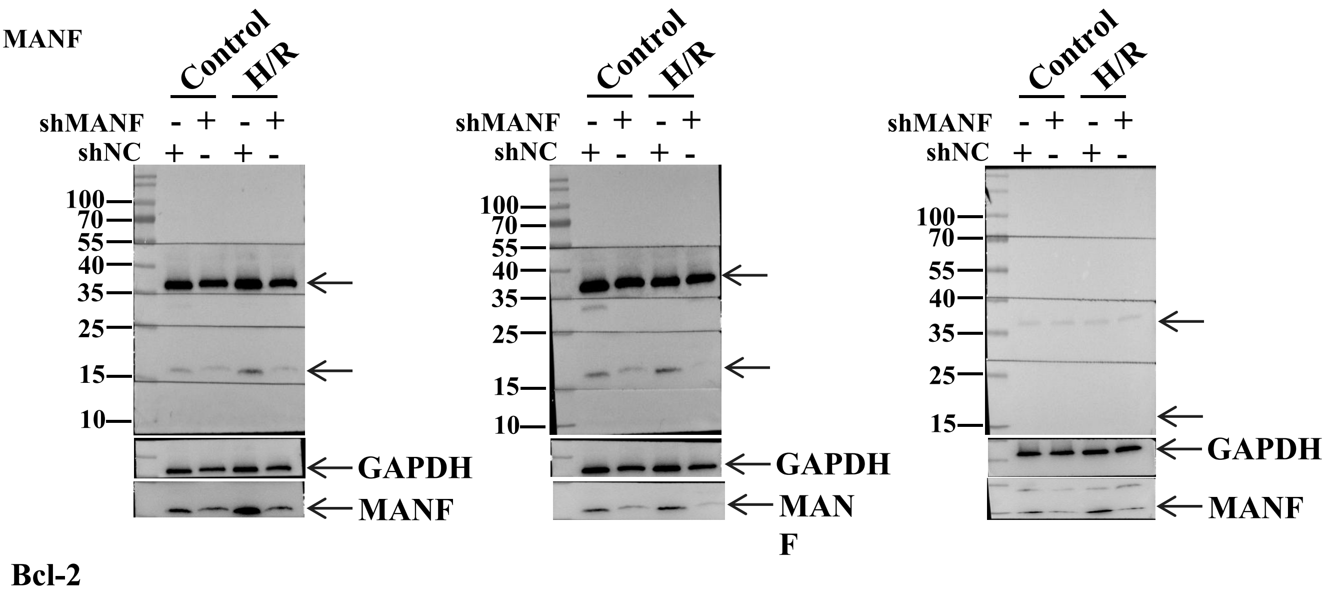

Bcl-2

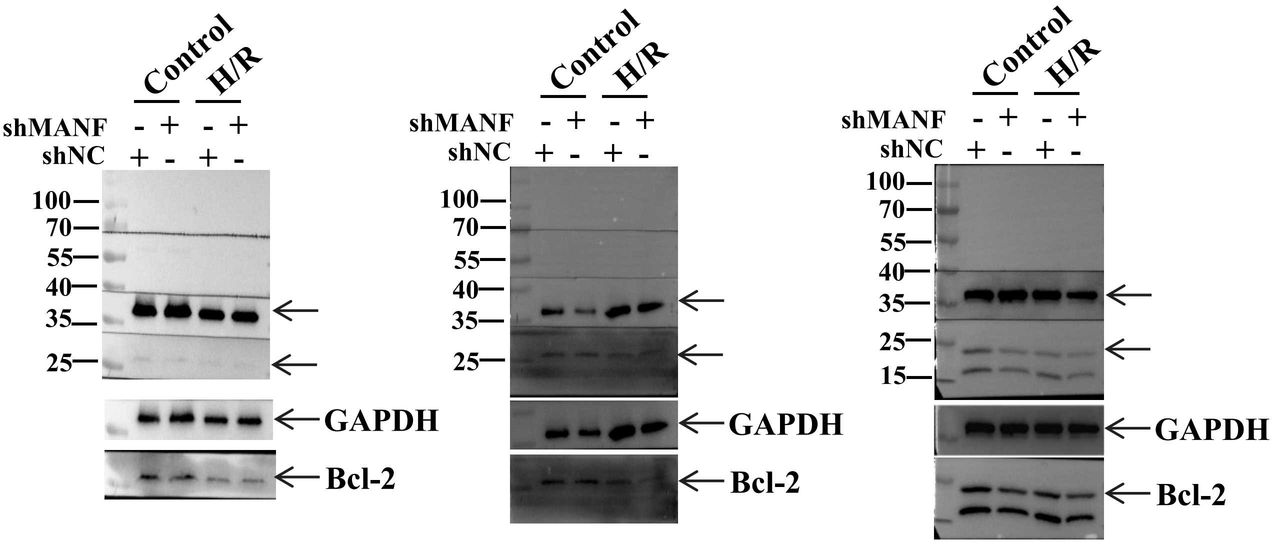

Bax

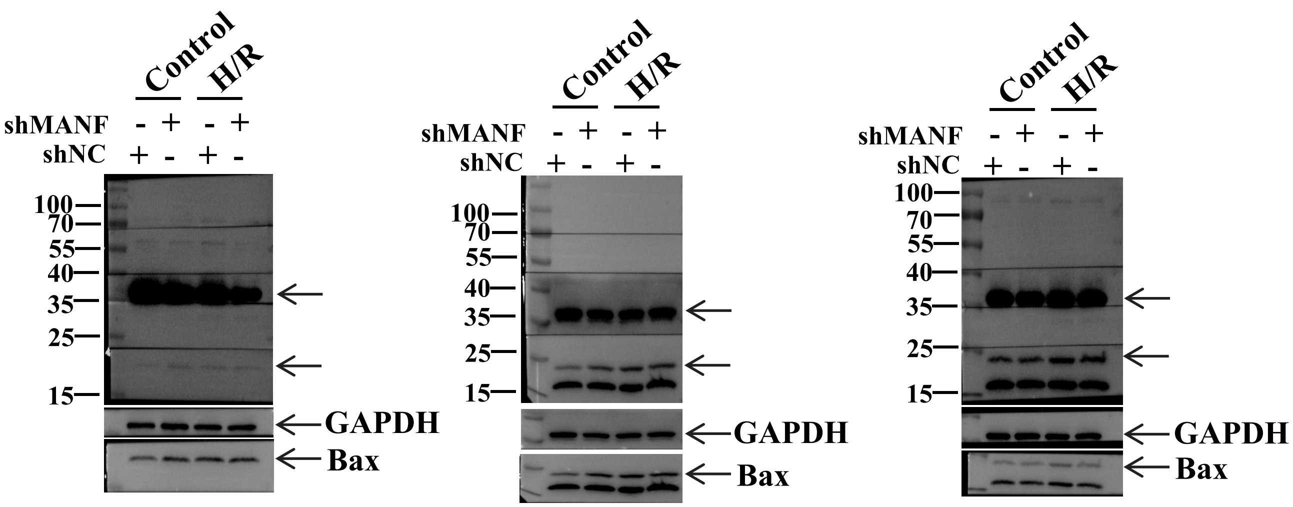

Cleaved-Caspase3

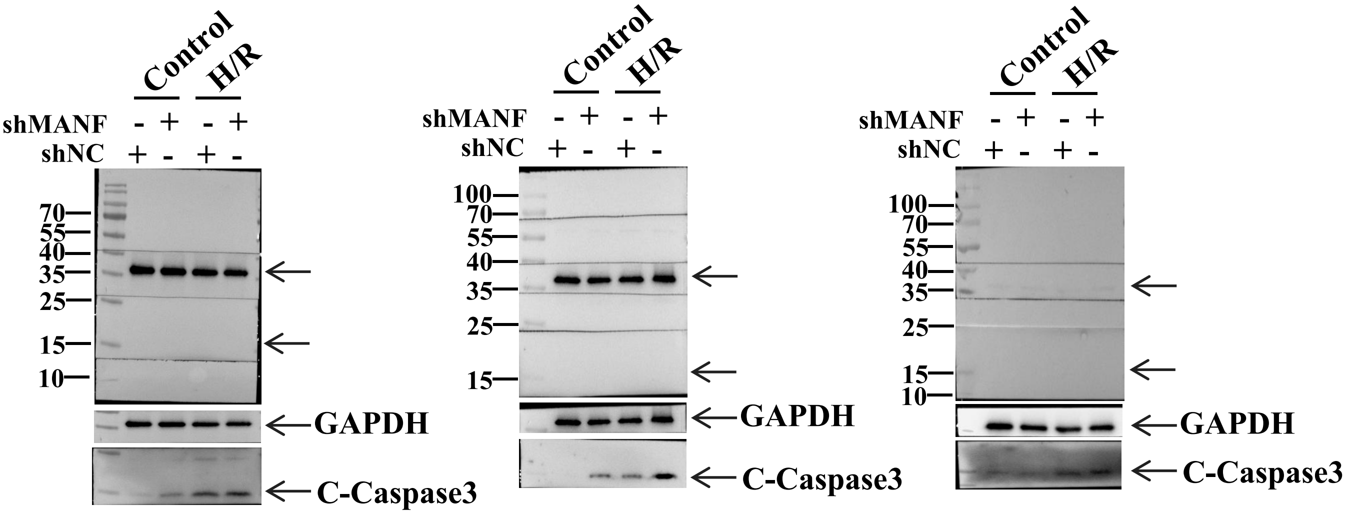

Figure 2 H

ATF6

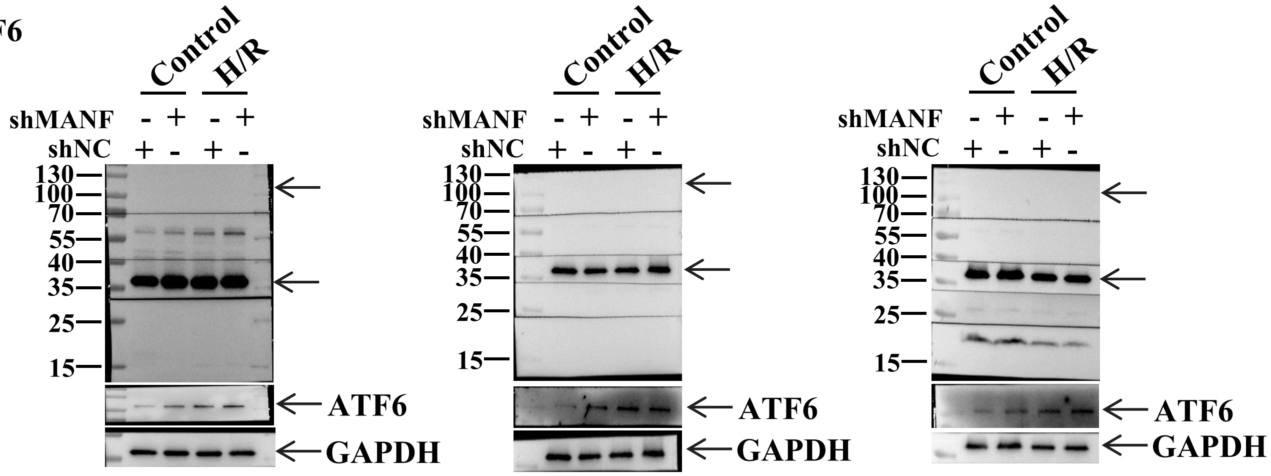

p-IRE1α

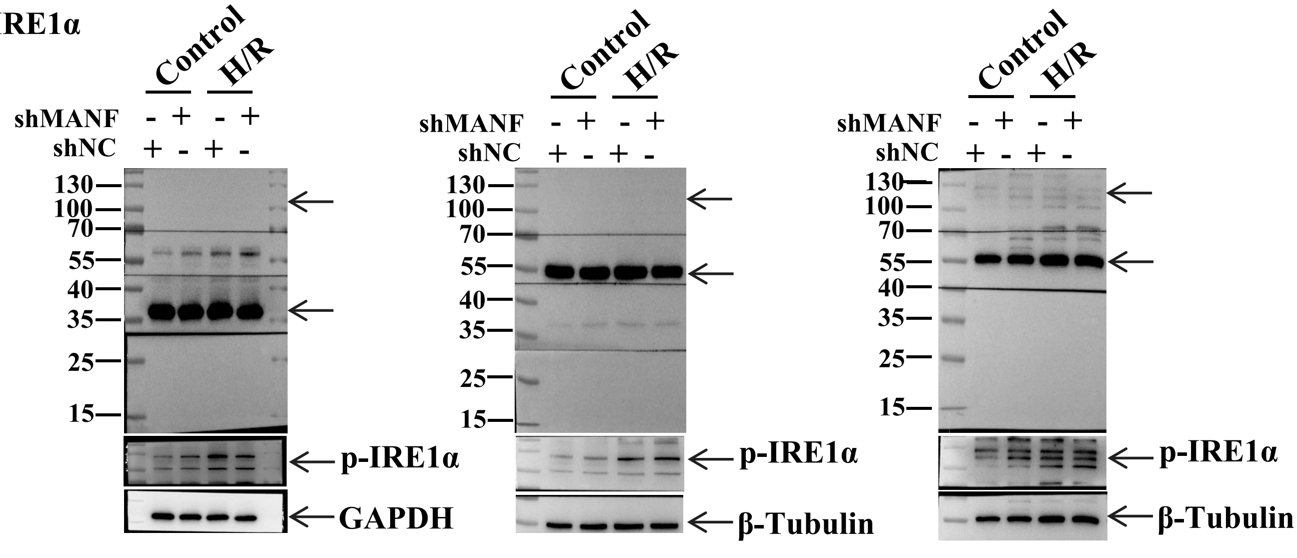

p-PERK

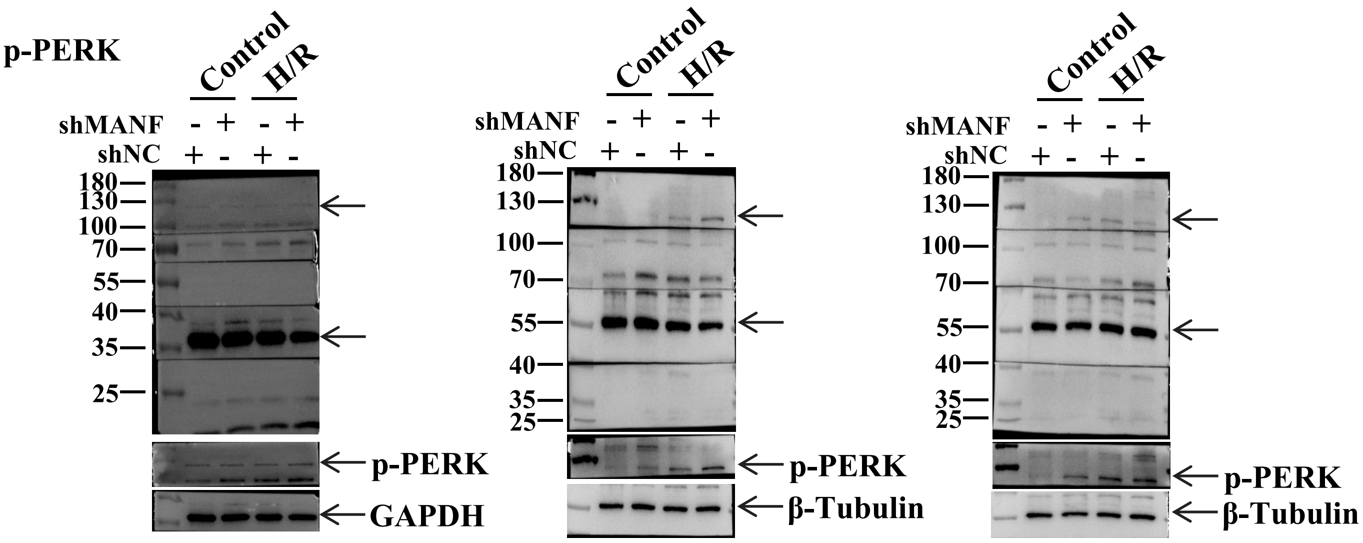

BiP

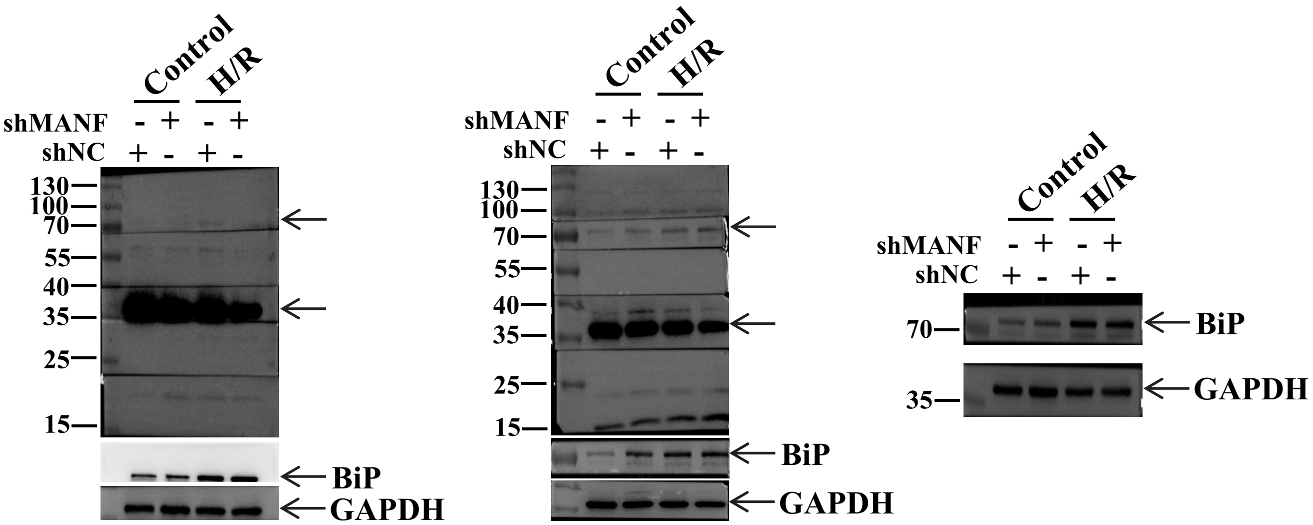

p-eIF2α

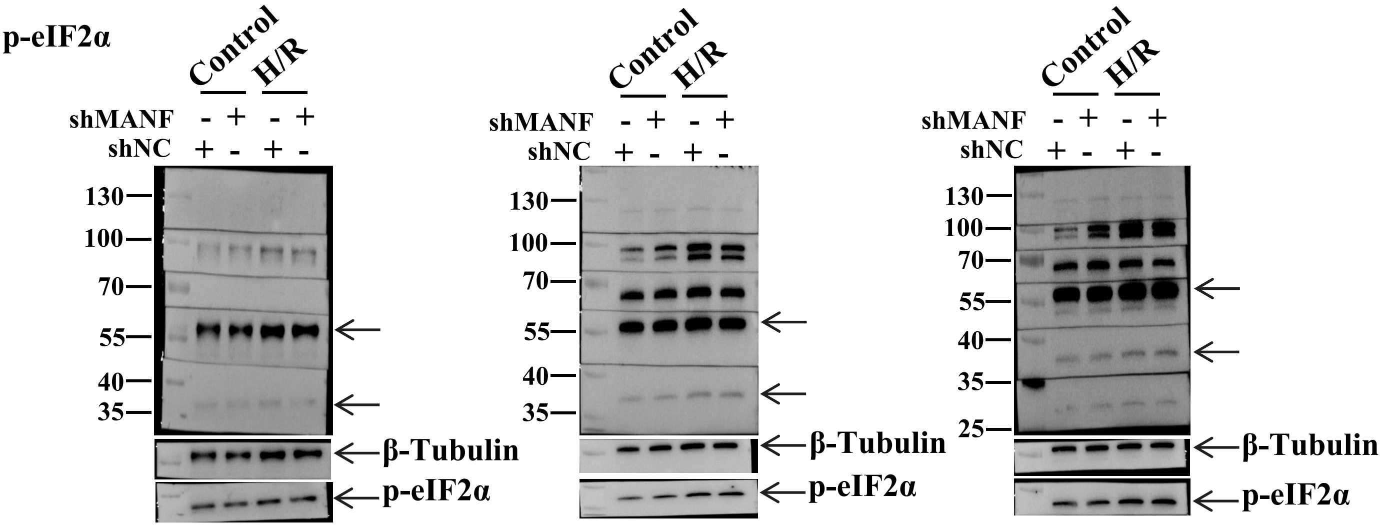

ATF4

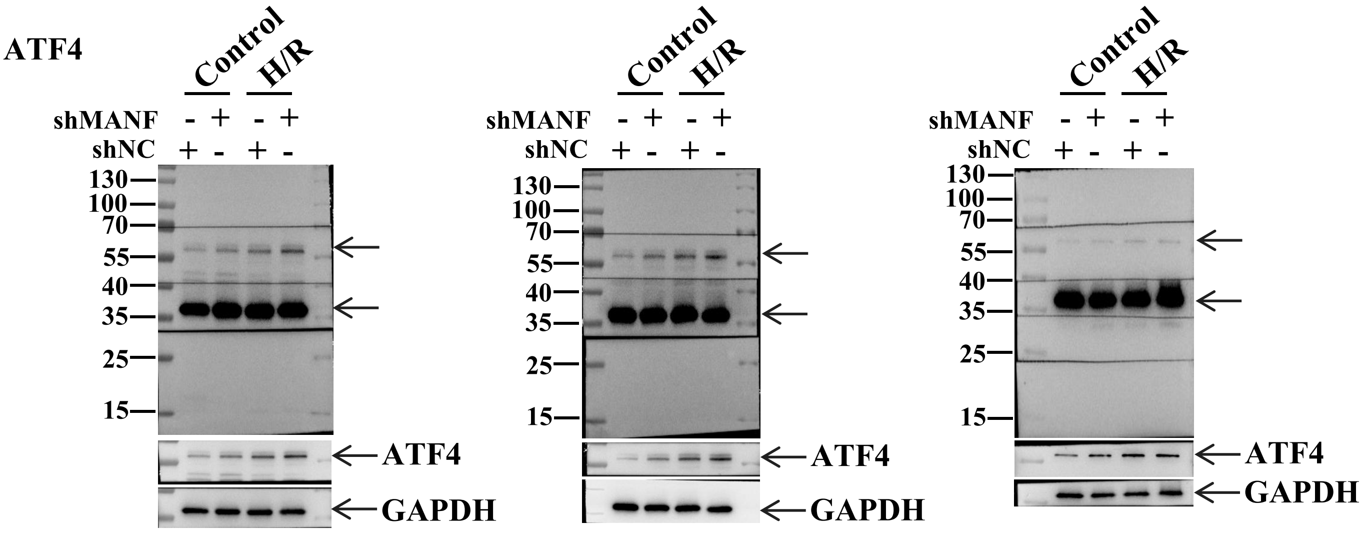
Control
H/R
shMANF - + - +
shNC + - + -
130
100
70
55
40
35
25
15
ATF4
GAPDH

CHOP

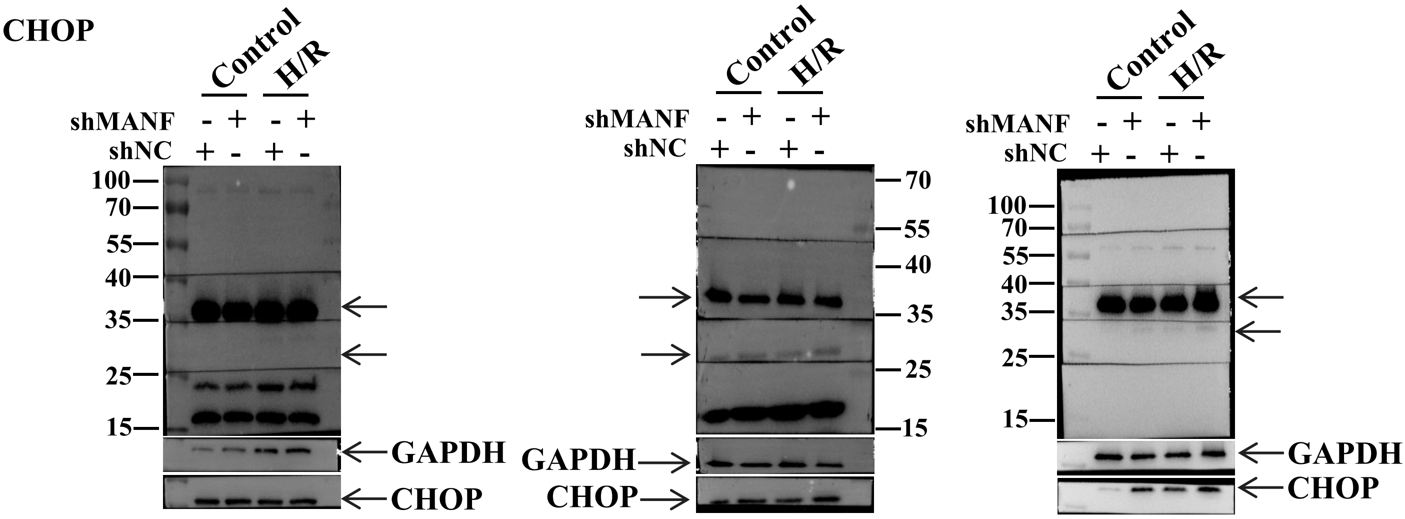
Control
H/R
shMANF - + - +
shNC + - + -
100
70
55
40
35
25
15
GAPDH
CHOP

Figure 3 A

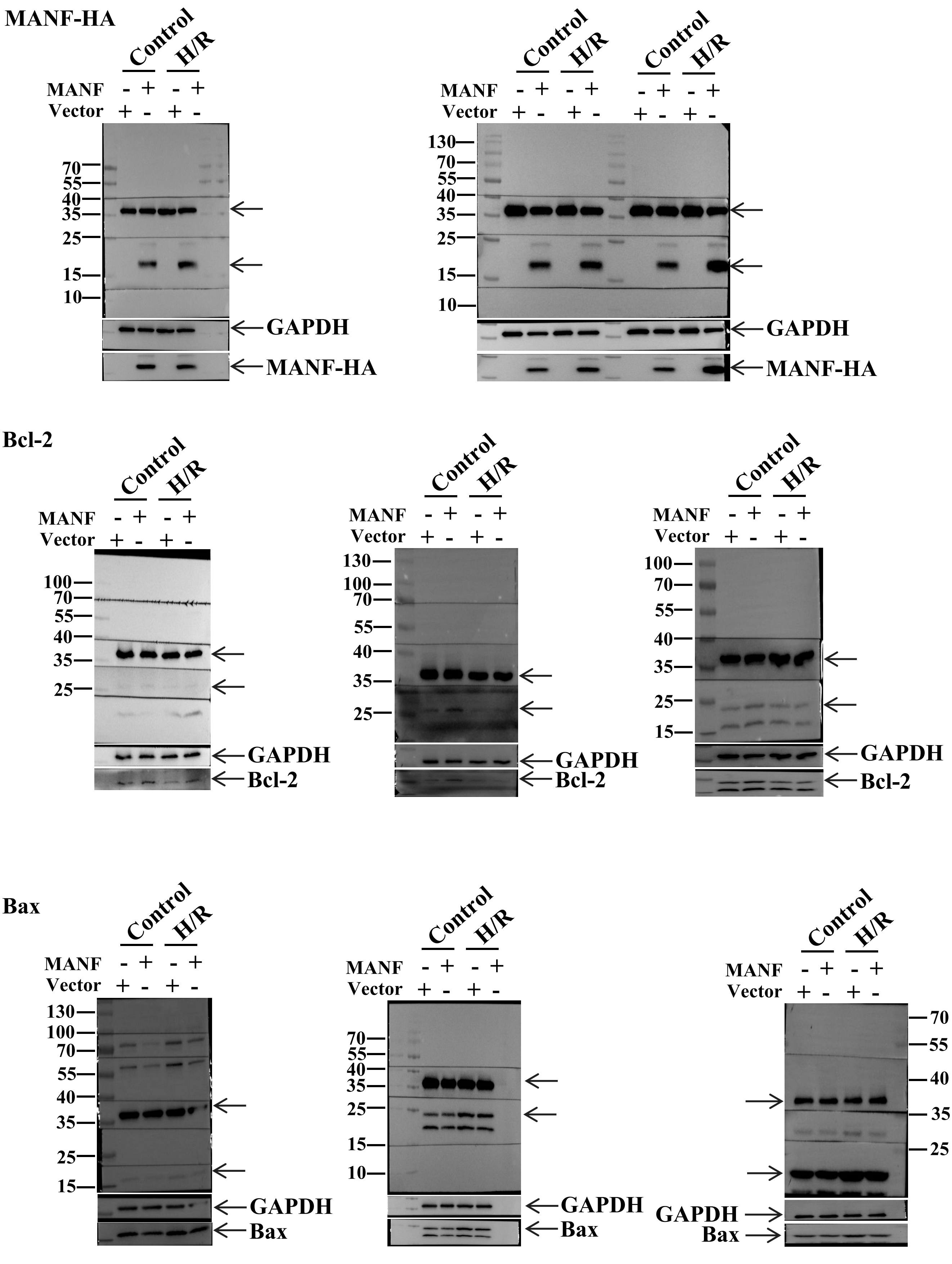
MANF-HA
Control
H/R
MANF
Vector
130
100
70
55
40
35
25
15
10
GAPDH
MANF-HA
Bcl-2
Bax

Cleaved-Caspase3

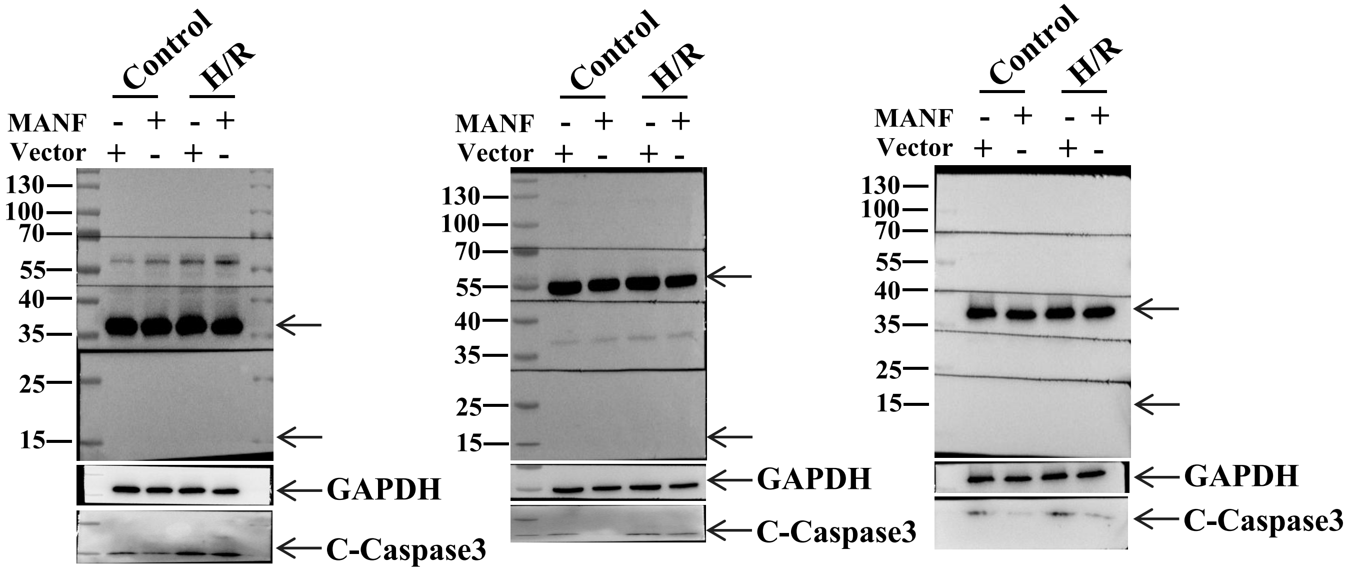

Figure 3 G

ATF6

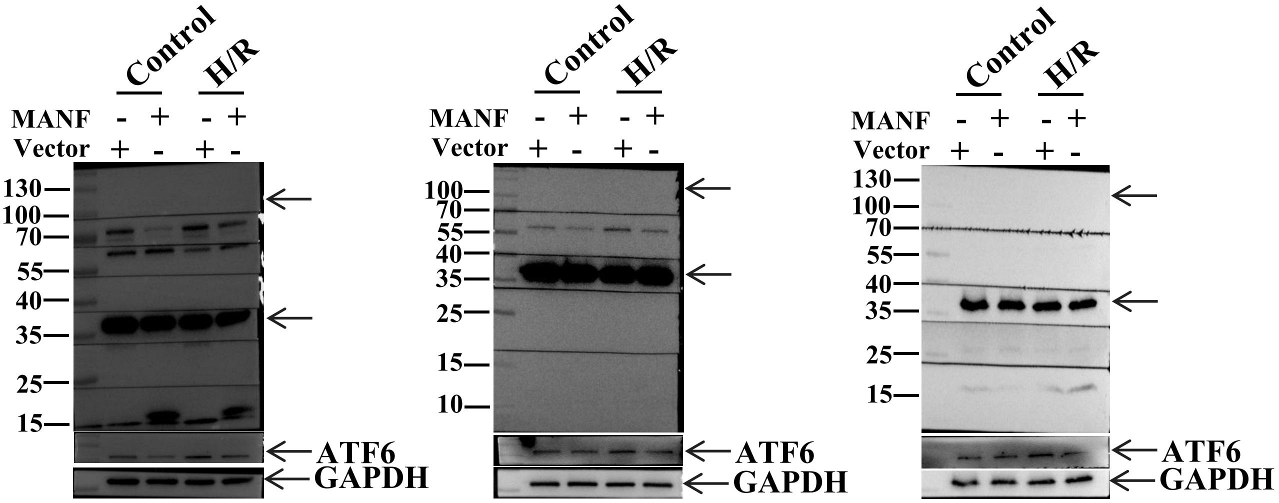

p-IRE1α

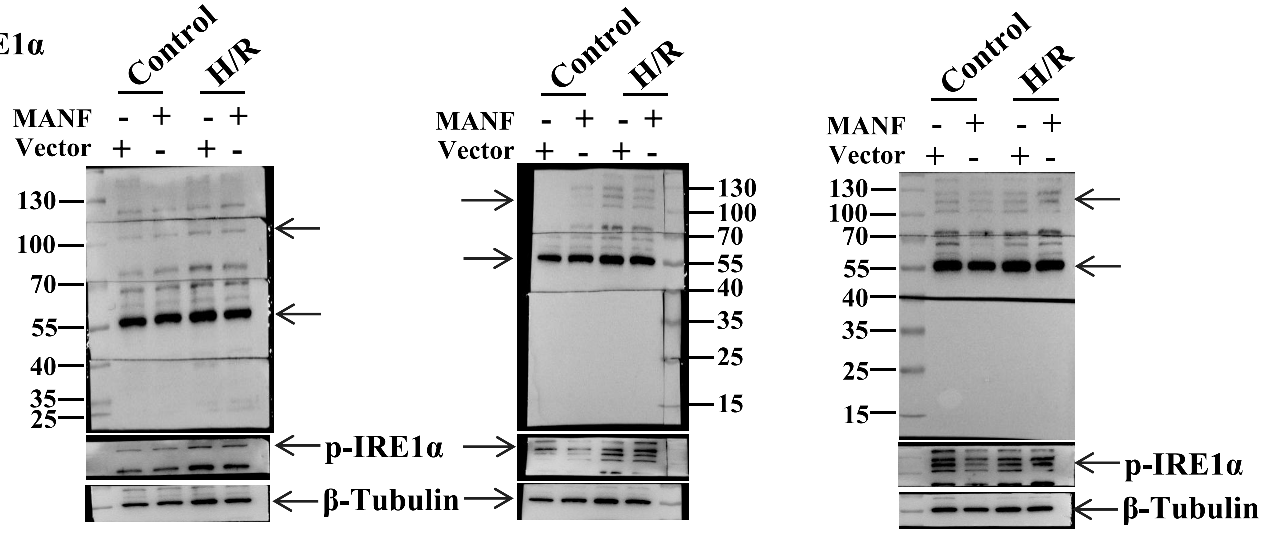

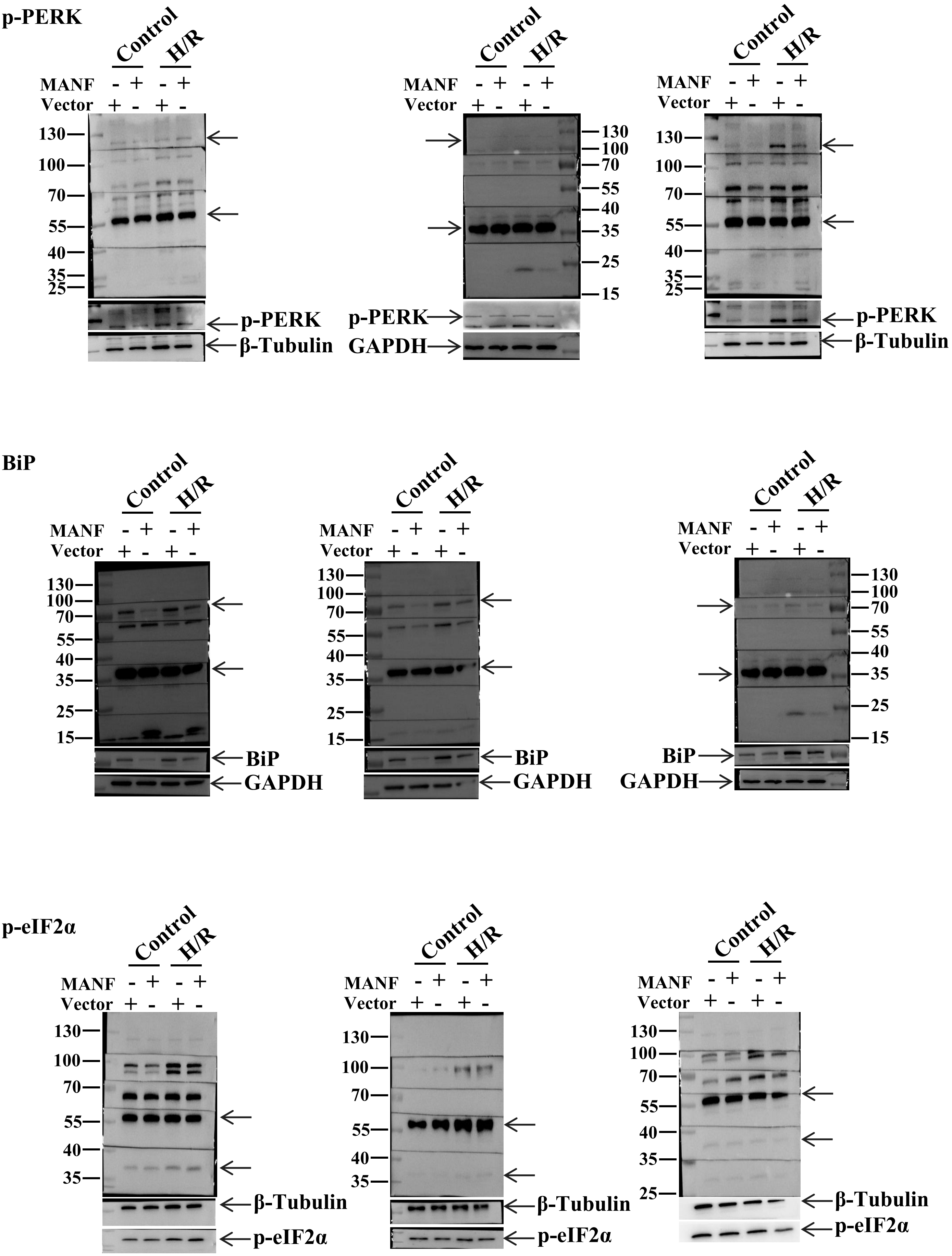
p-PERK
Control
H/R
MANF - + - +
Vector + - + -
130
100
70
55
40
35
25
15
p-PERK
β-Tubulin
GAPDH
BiP
BiP
GAPDH
p-eIF2α
β-Tubulin
p-eIF2α

ATF4

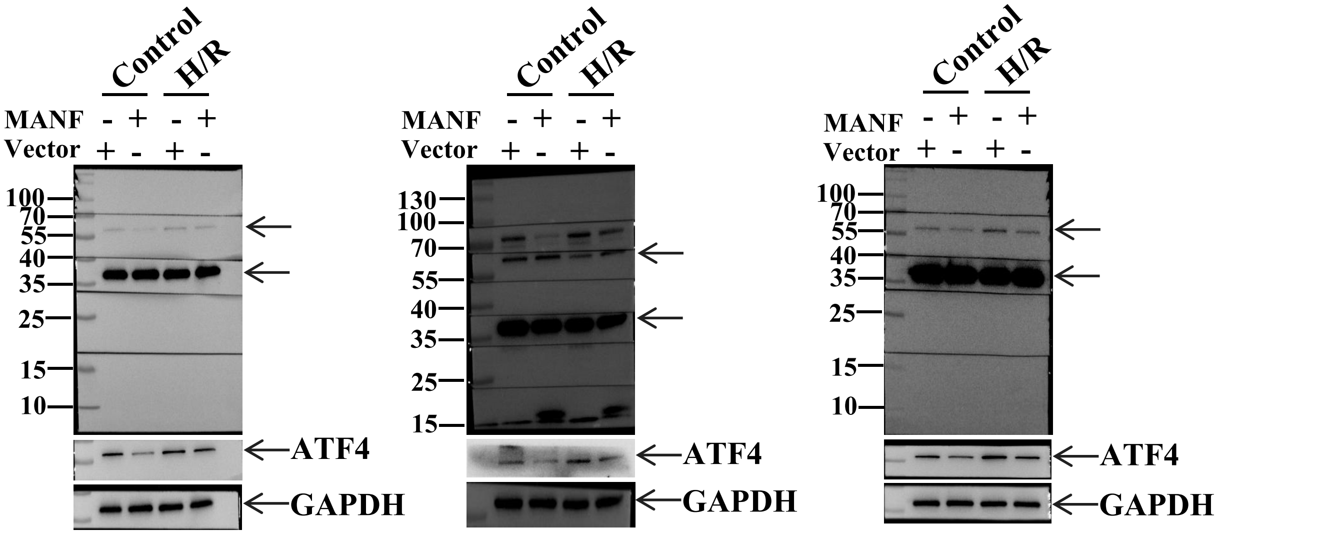
Control
H/R
MANF
Vector
100
70
55
40
35
25
15
10
130
ATF4
GAPDH

CHOP

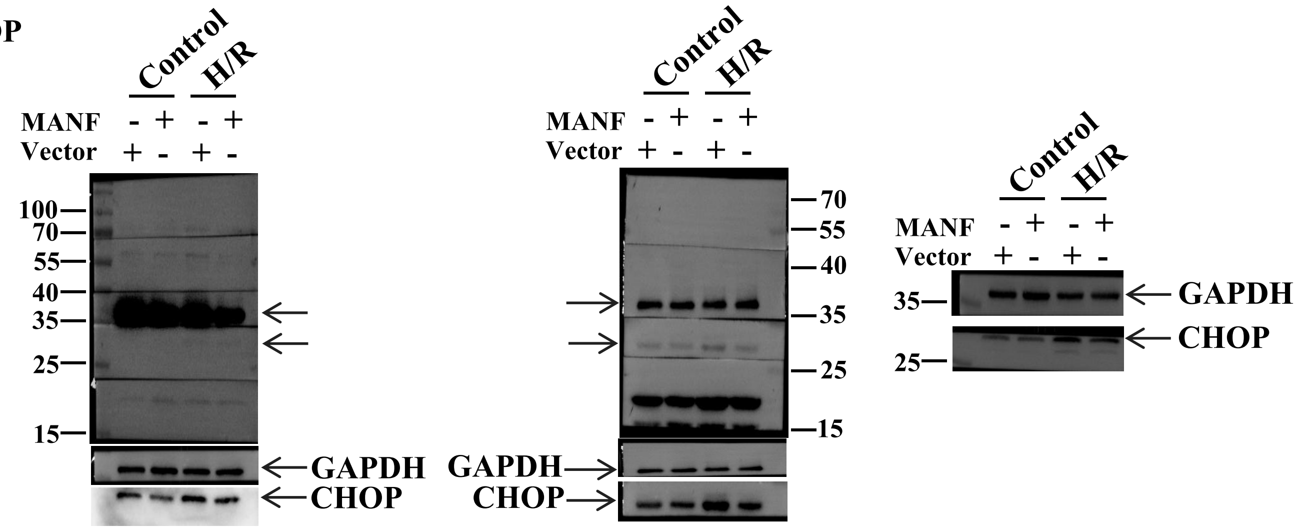
Control
H/R
MANF
Vector
100
70
55
40
35
25
15
GAPDH
CHOP

Figure 4 N

Bcl-2

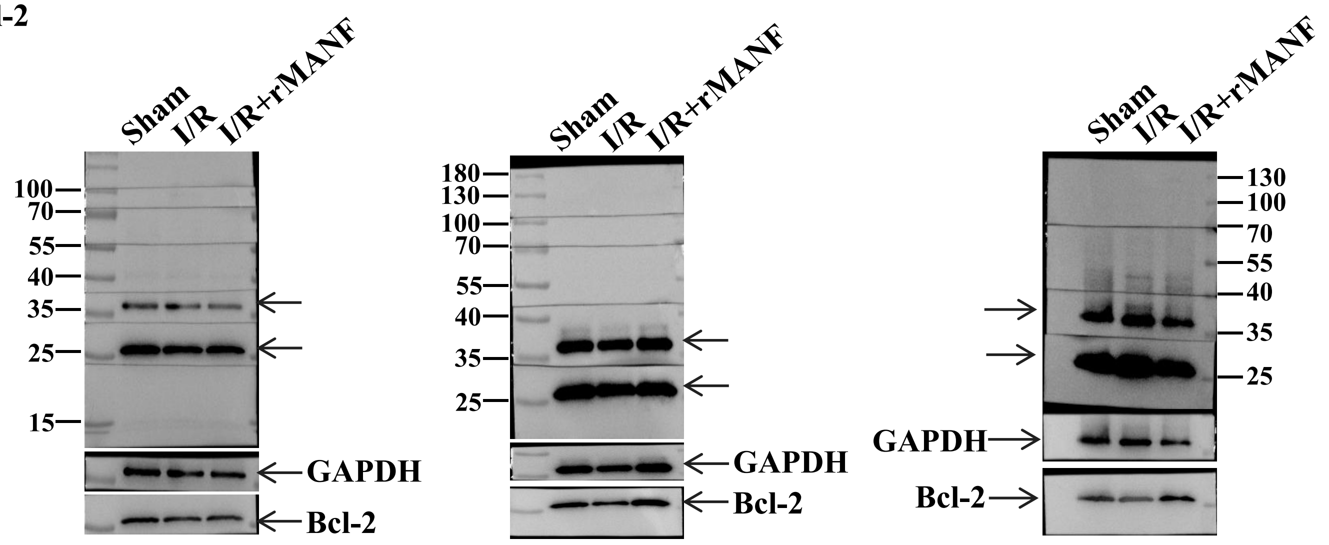

Bax

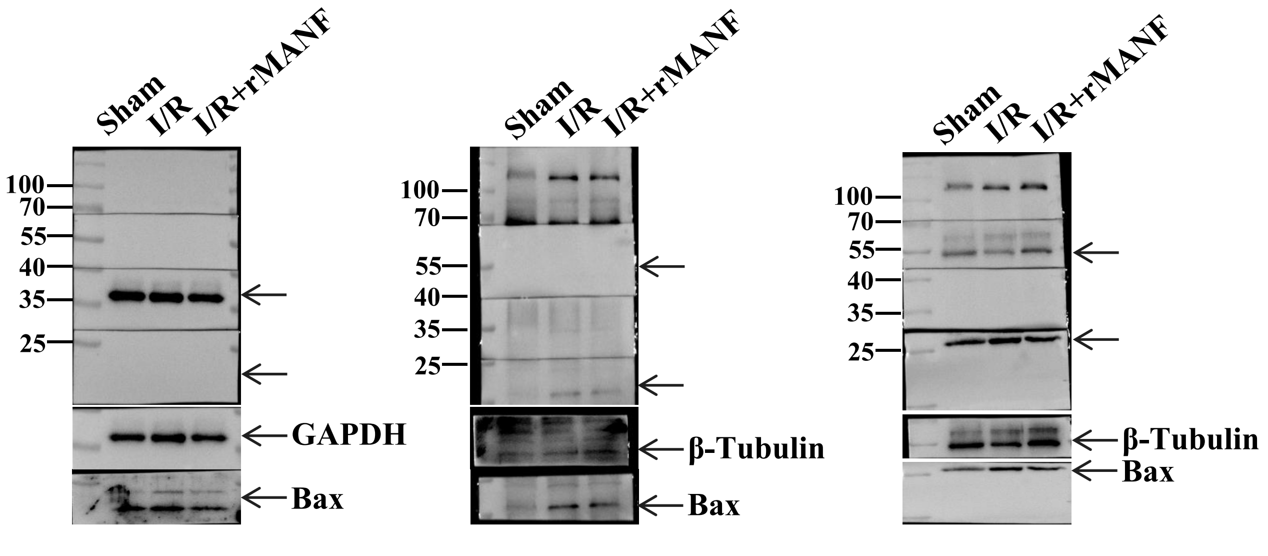

C-Caspase3

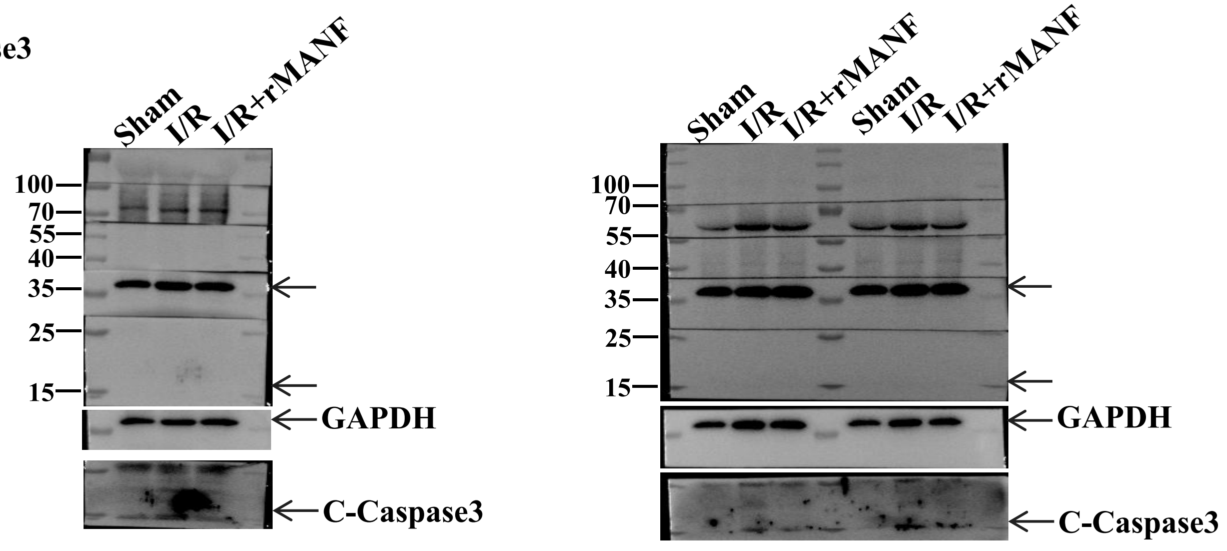

BiP

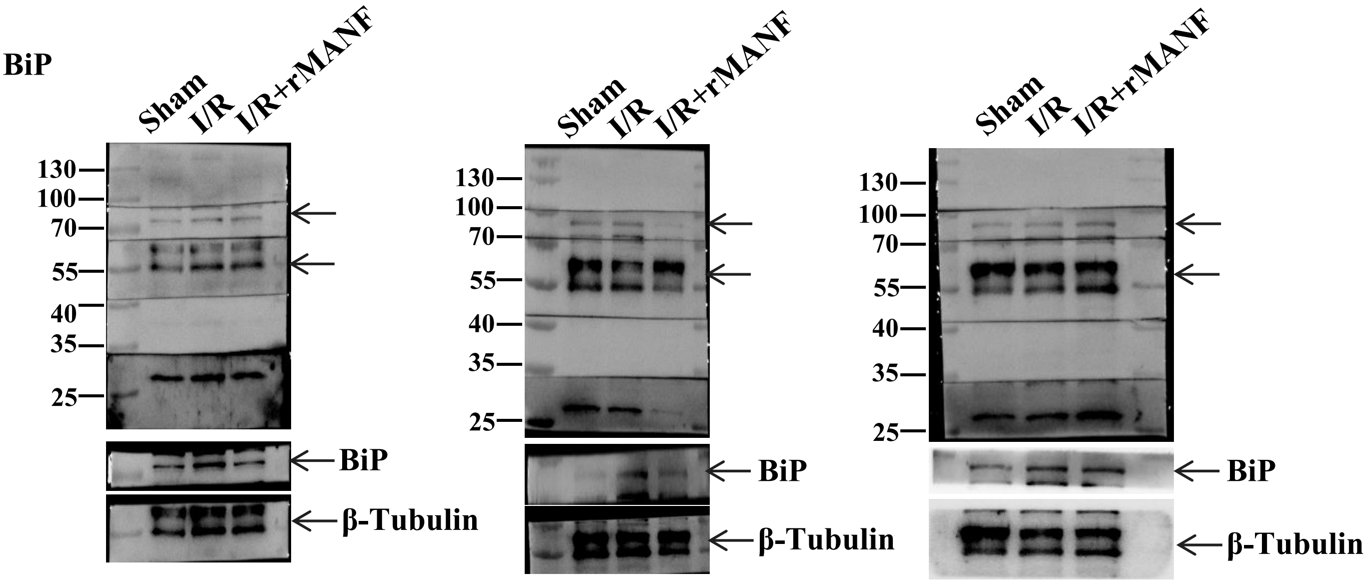

CHOP

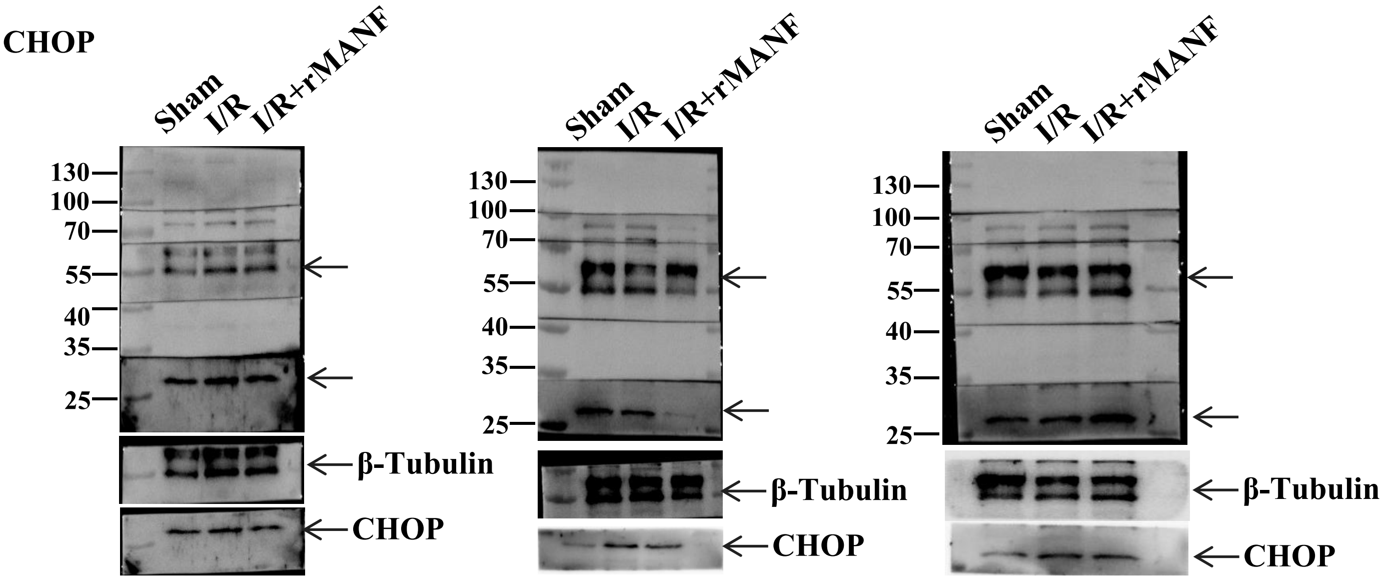

ATF6

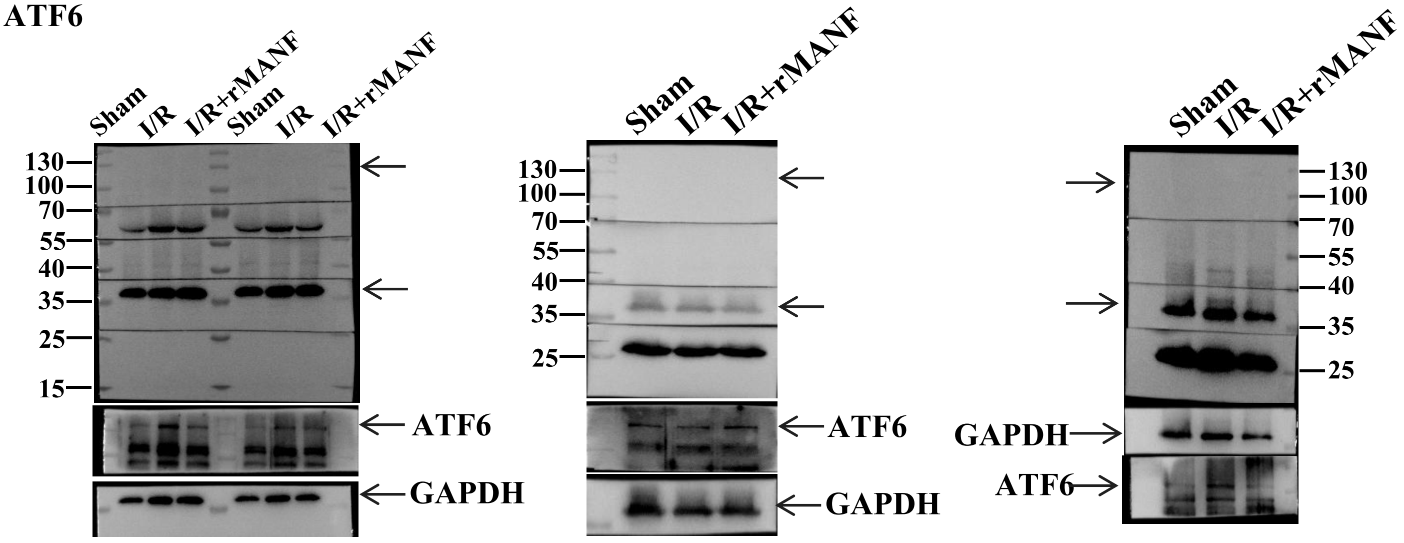

ATF6

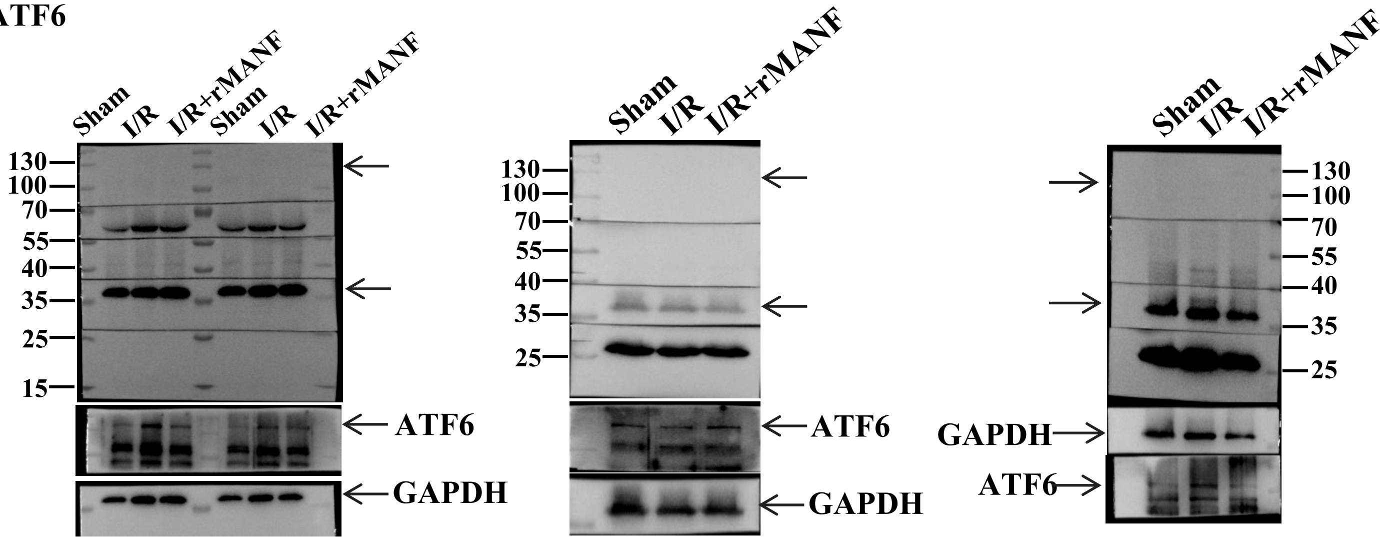

p-PERK

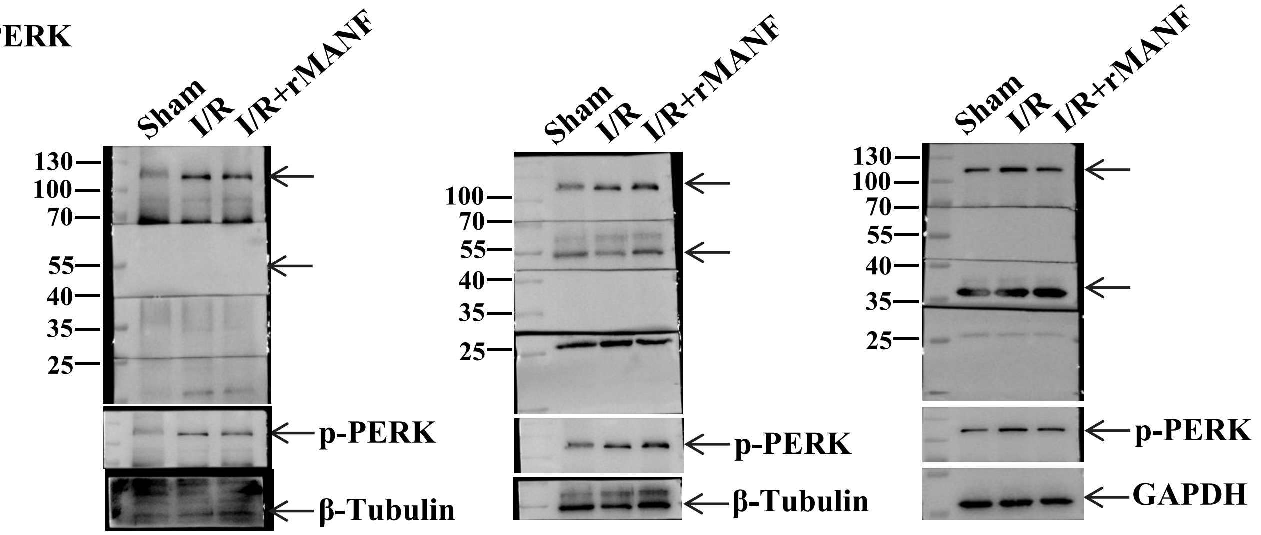

p-eIF2α

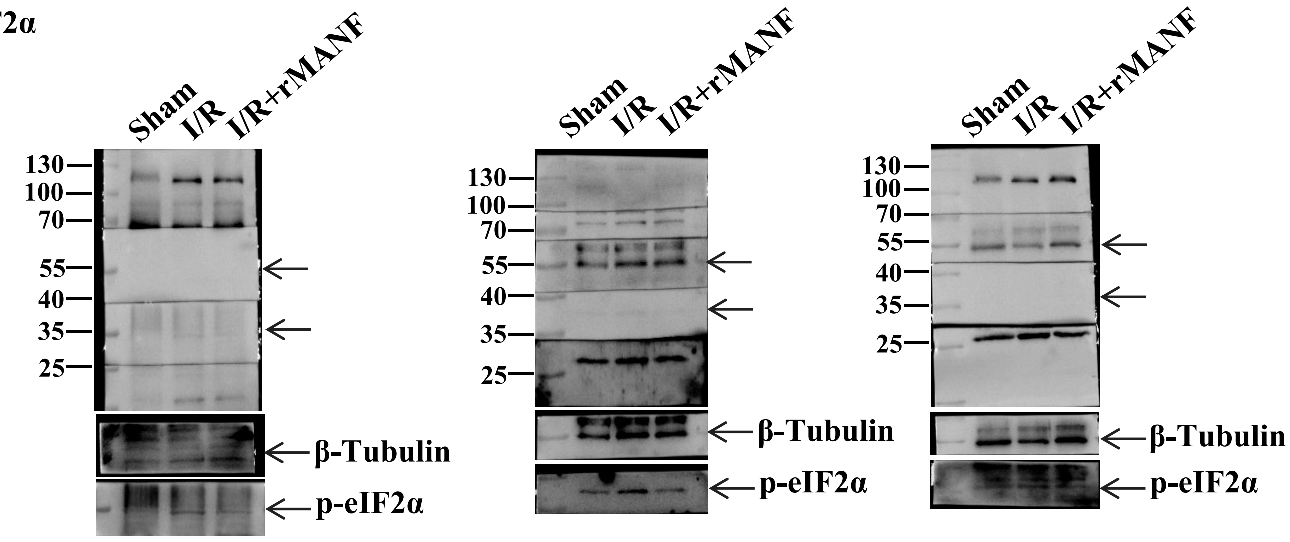

ATF4

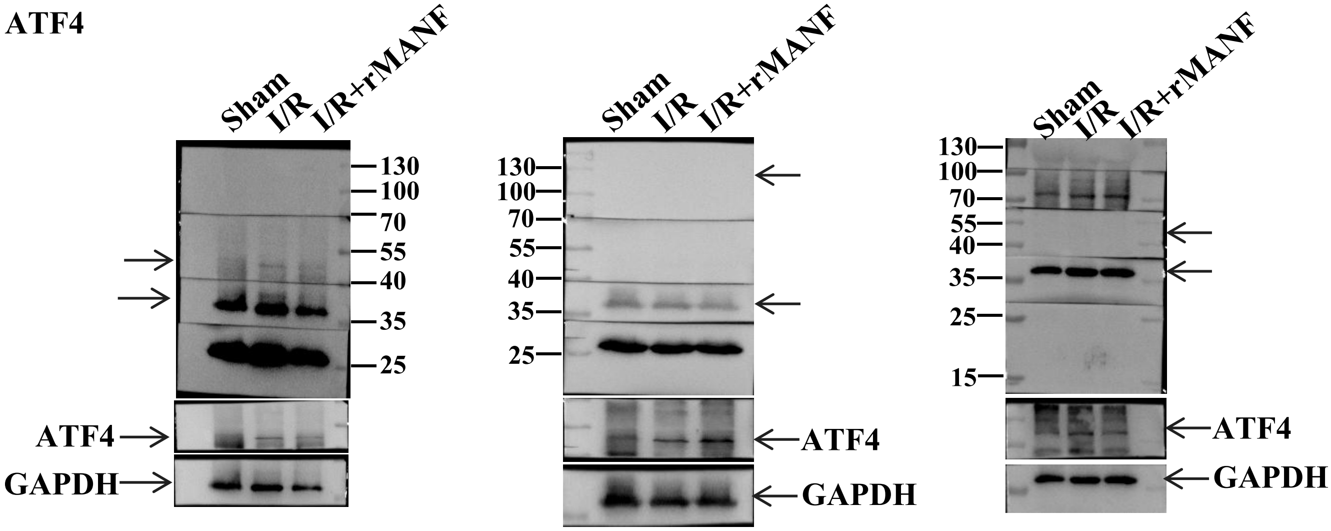

Figure 5 B

Cell lysates

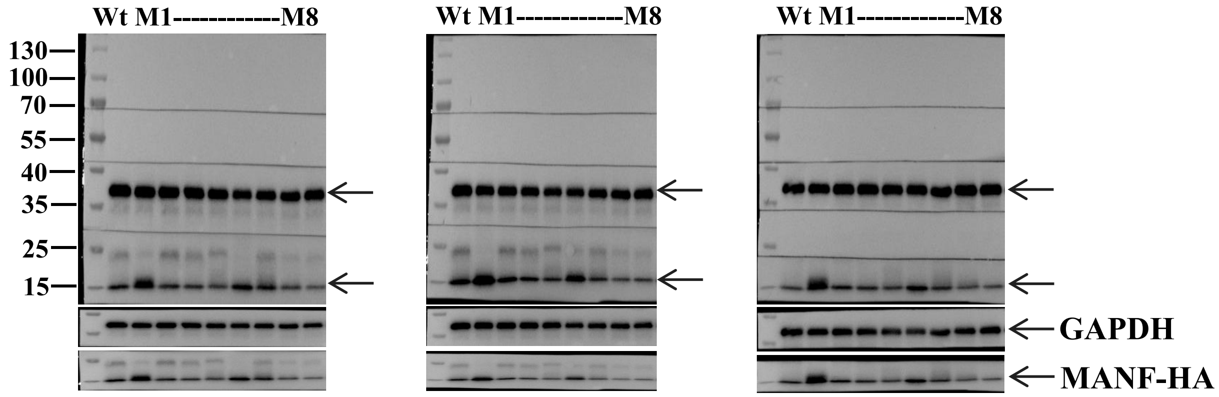

Mediums

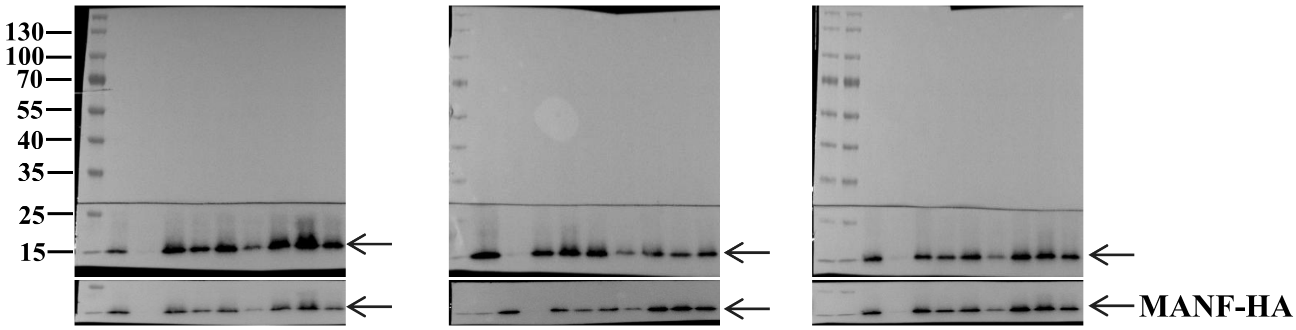

Figure 6 A

MANF-HA

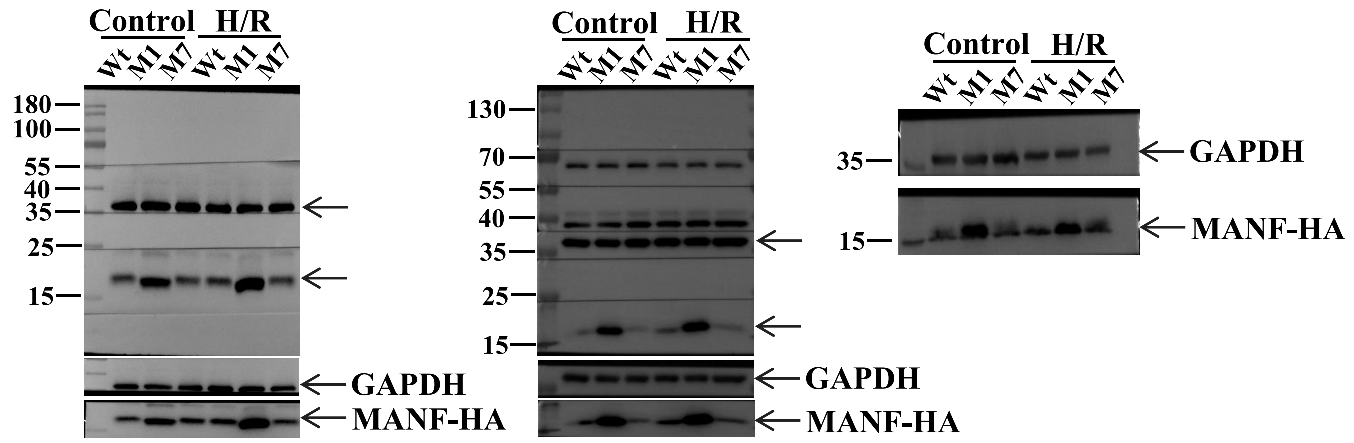

Bcl-2

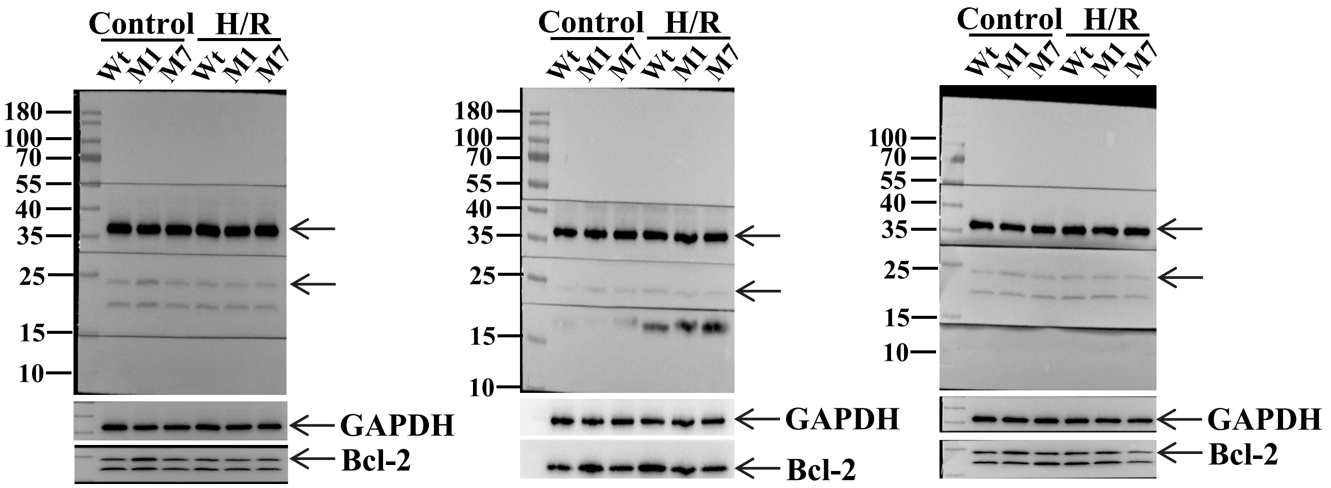

Bax

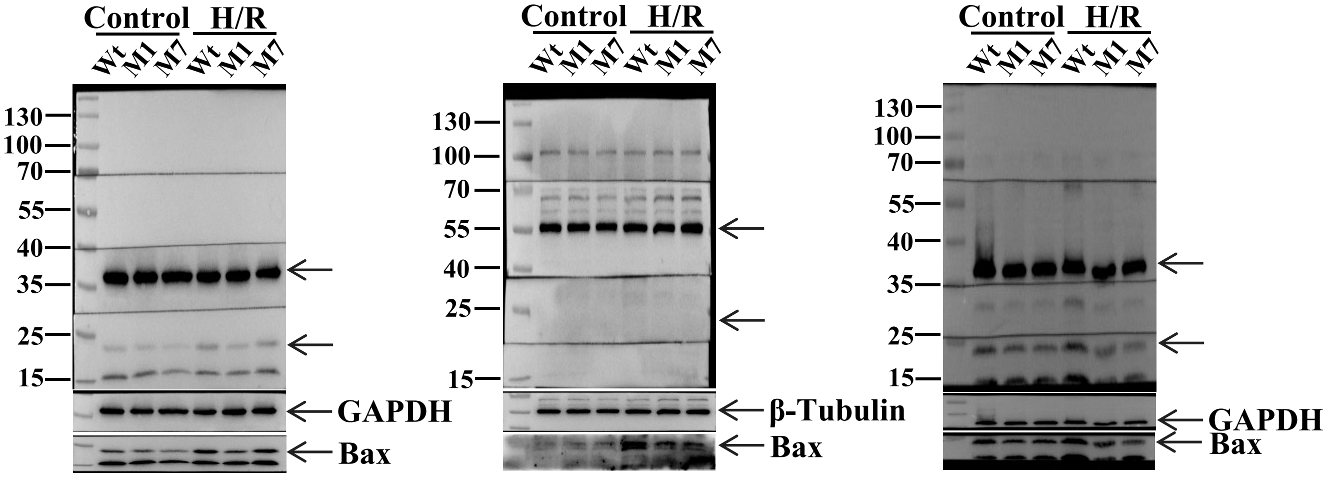

Cleaved-Caspase3

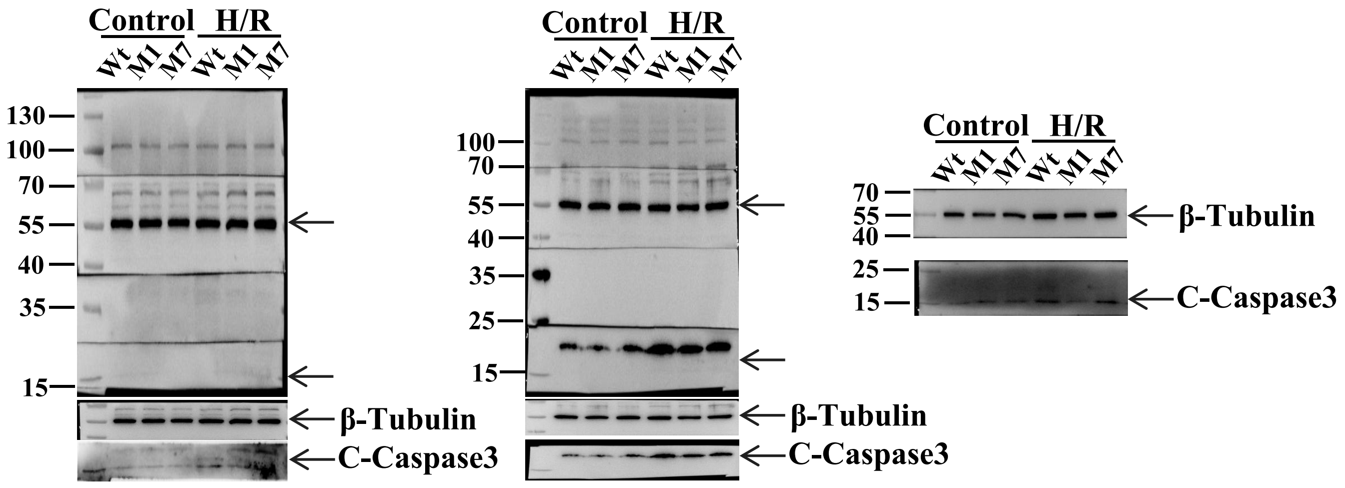

ATF6

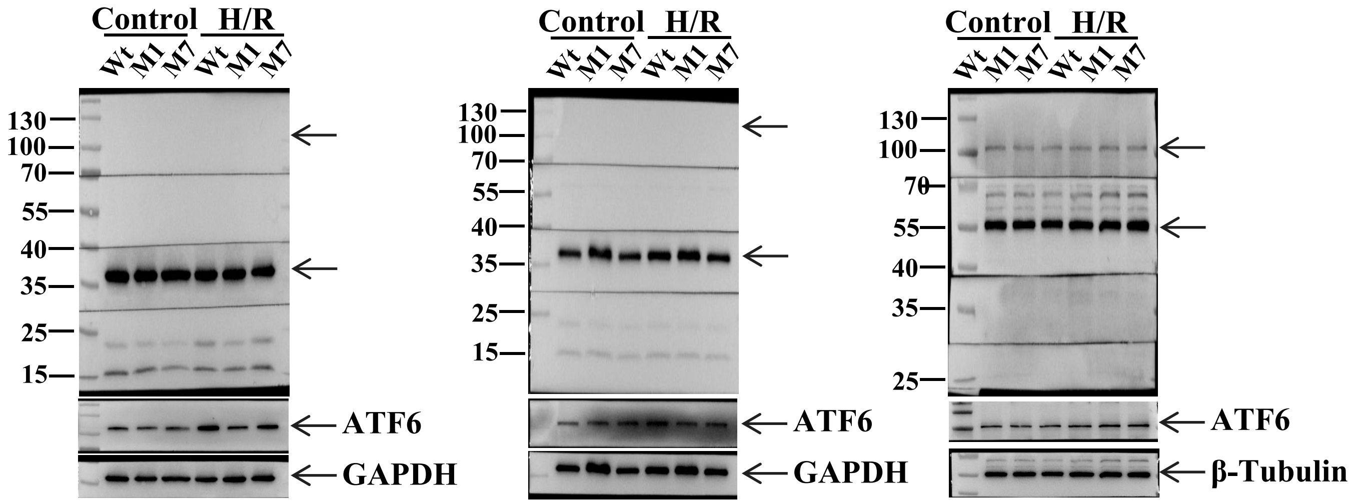

p-IRE1α

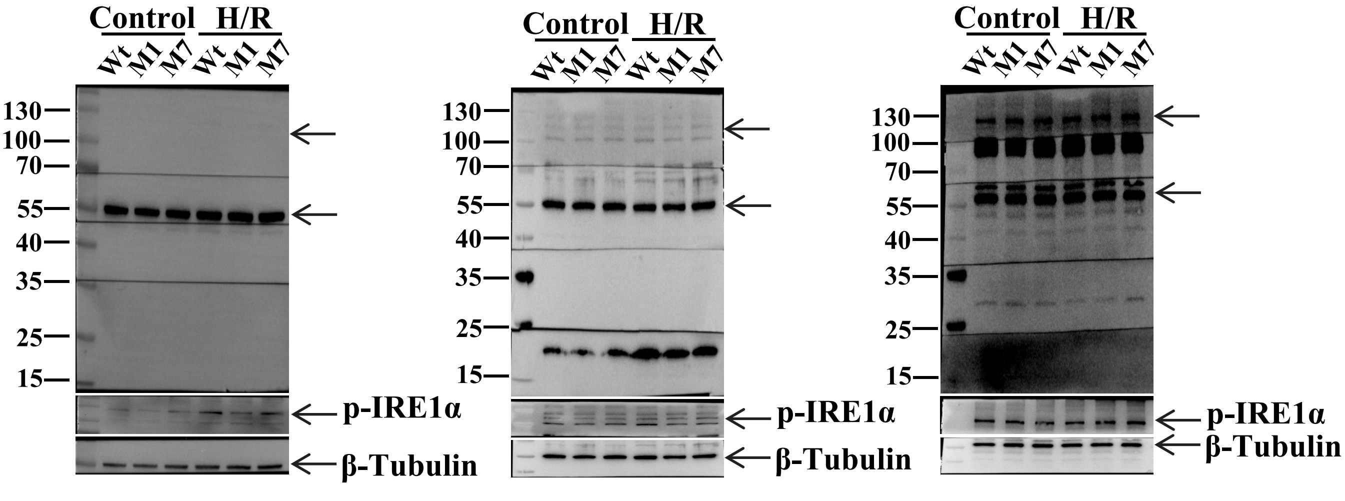

p-PERK

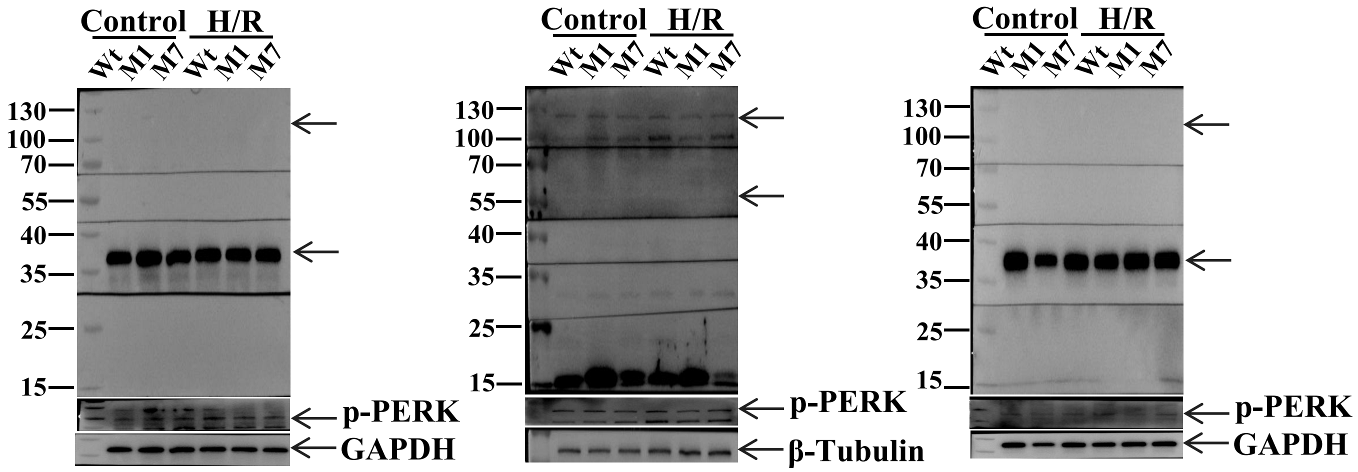

BiP

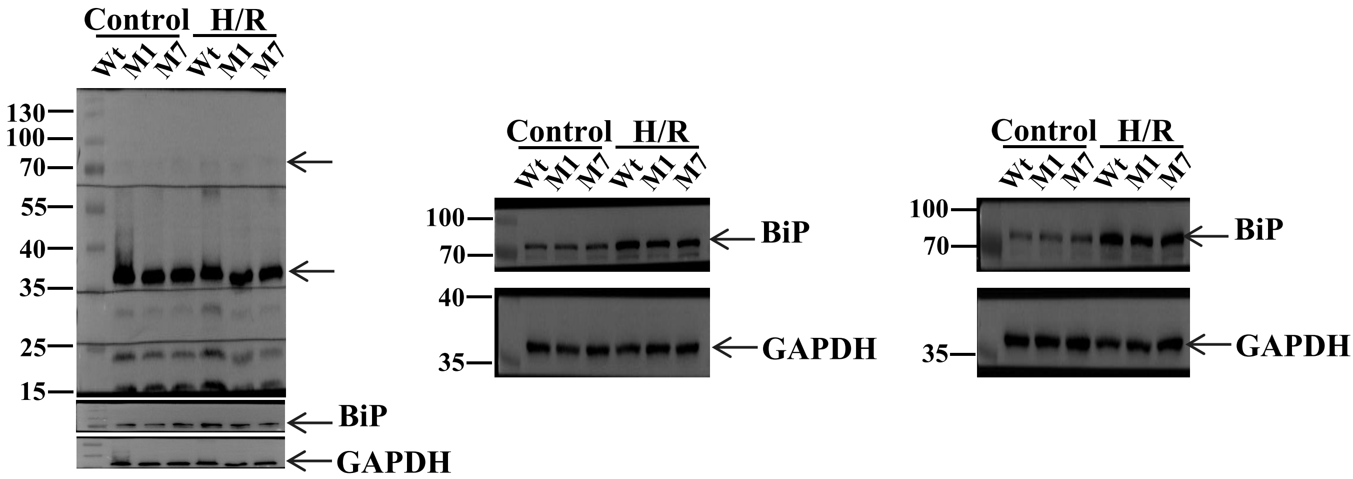

p-eIF2α

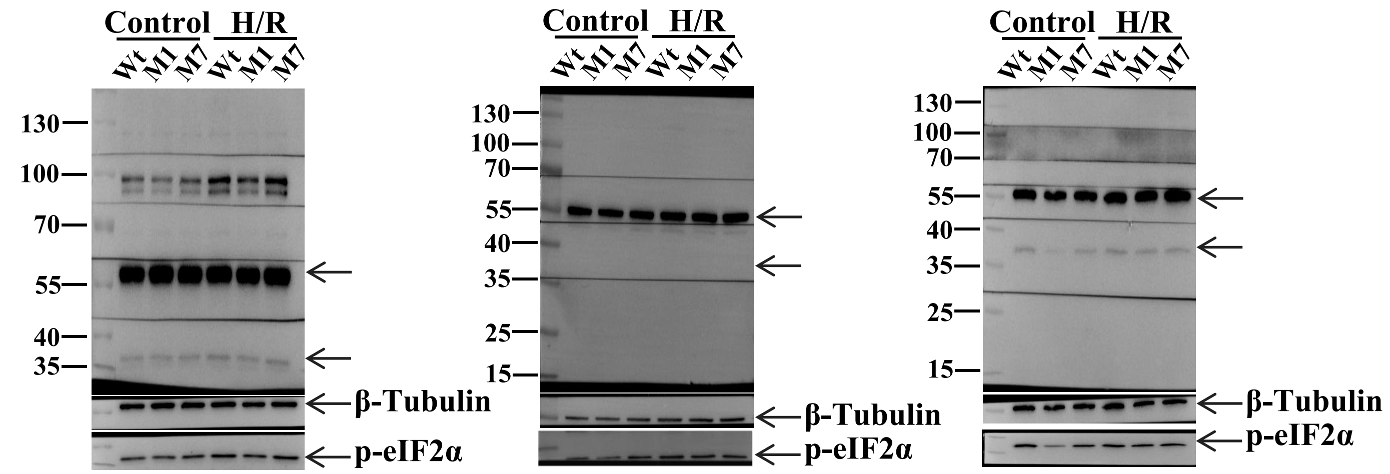

ATF4

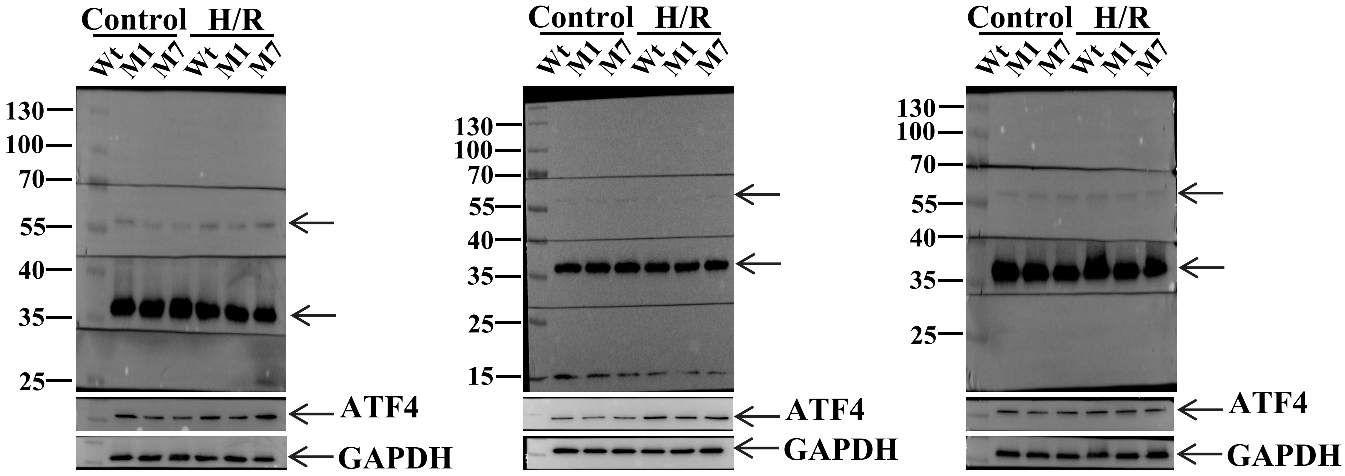

Control
H/R
Wt
M1
M7
Wt
M1
M7
130
100
70
55
40
35
25
15
ATF4
GAPDH

CHOP

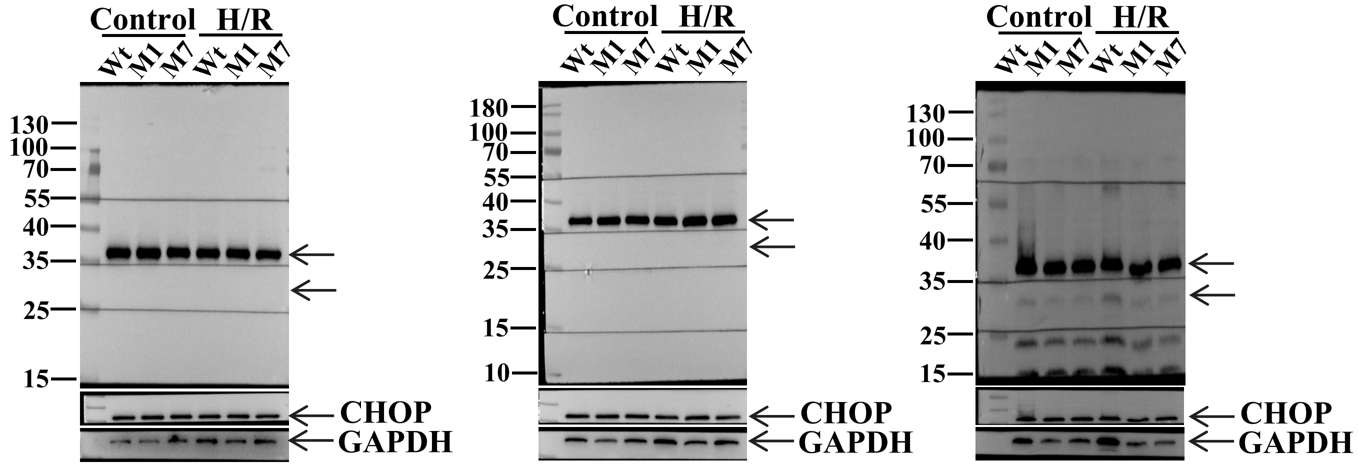

Control
H/R
Wt
M1
M7
Wt
M1
M7
180
130
100
70
55
40
35
25
15
10
CHOP
GAPDH

Figure 7 A

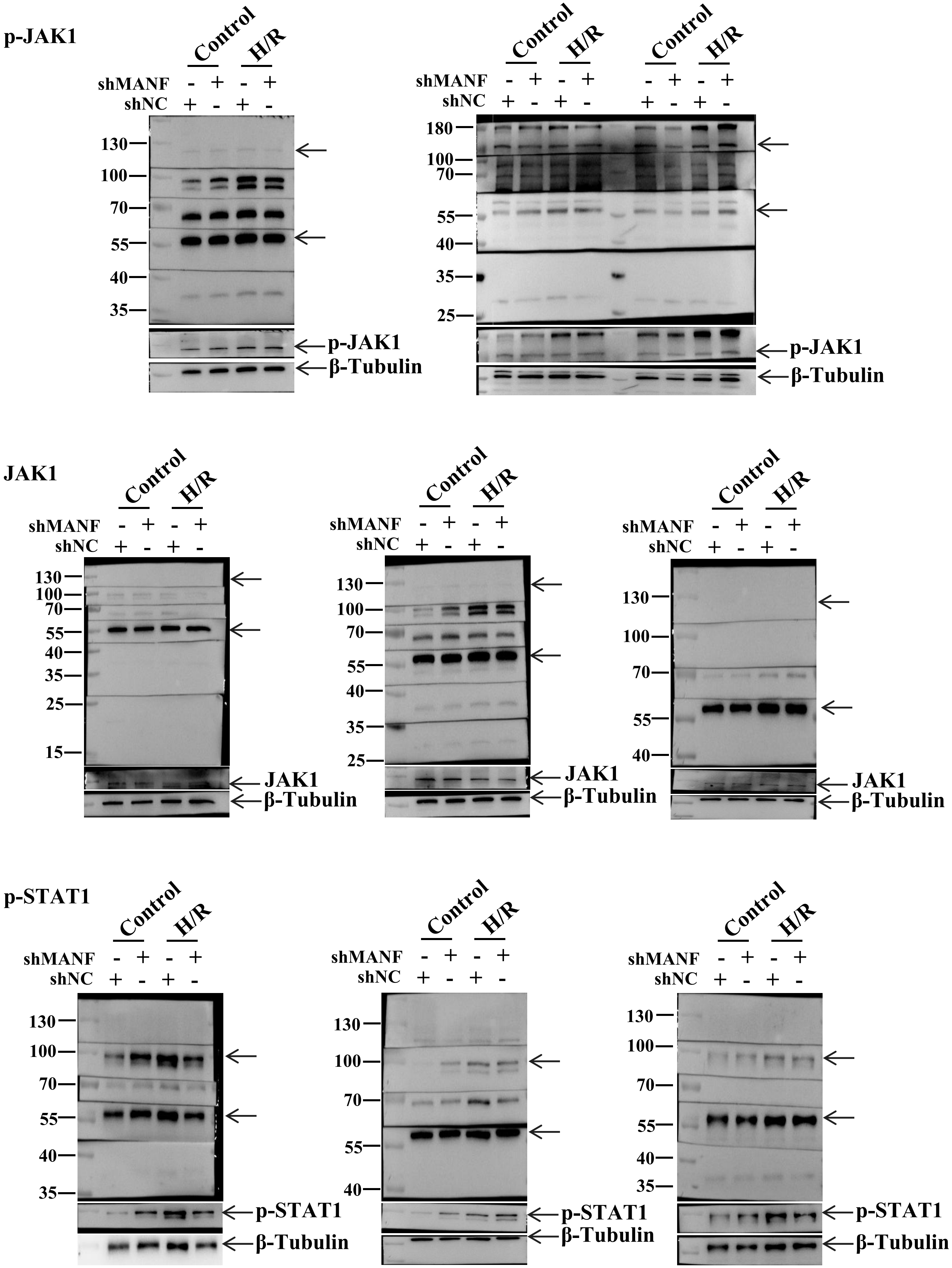
p-JAK1
Control
H/R
shMANF - + - +
shNC + - + -
130
100
70
55
40
35
p-JAK1
β-Tubulin
180
25
JAK1
15
p-STAT1

STAT1

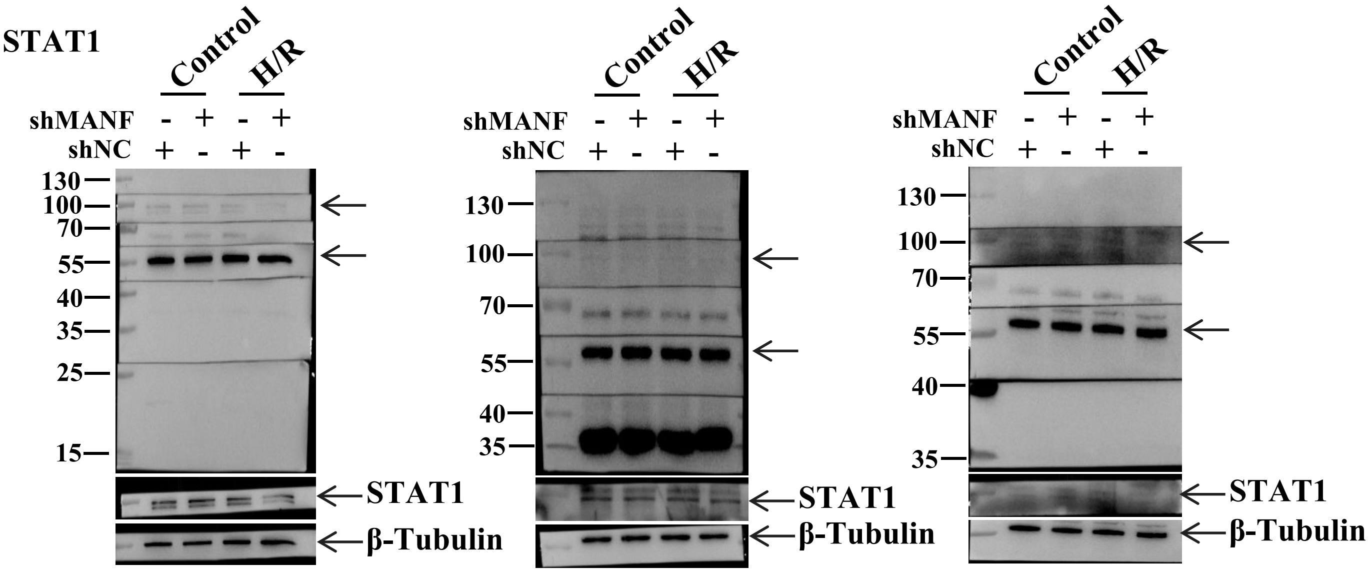

p-NF-κB

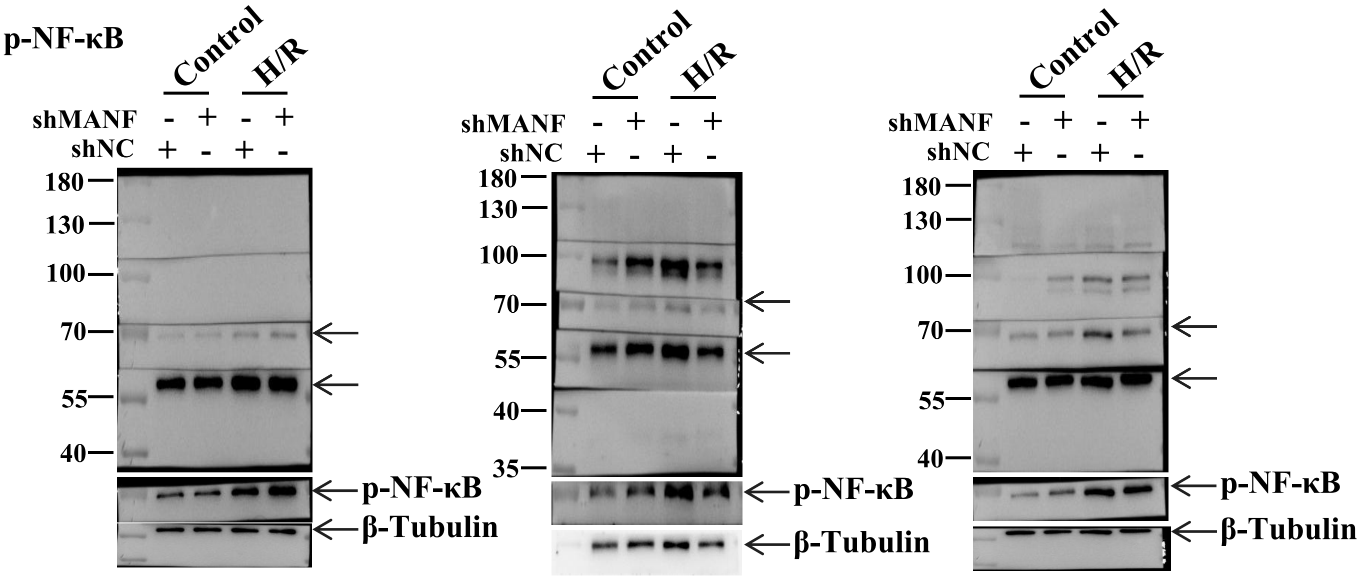

NF-κB

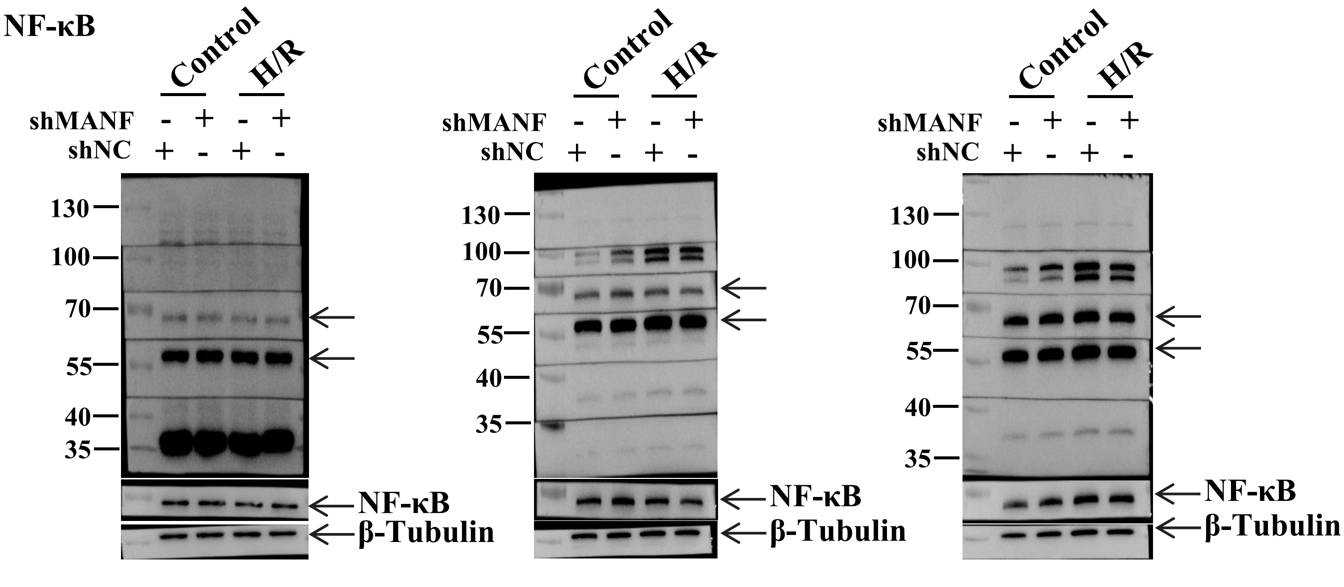

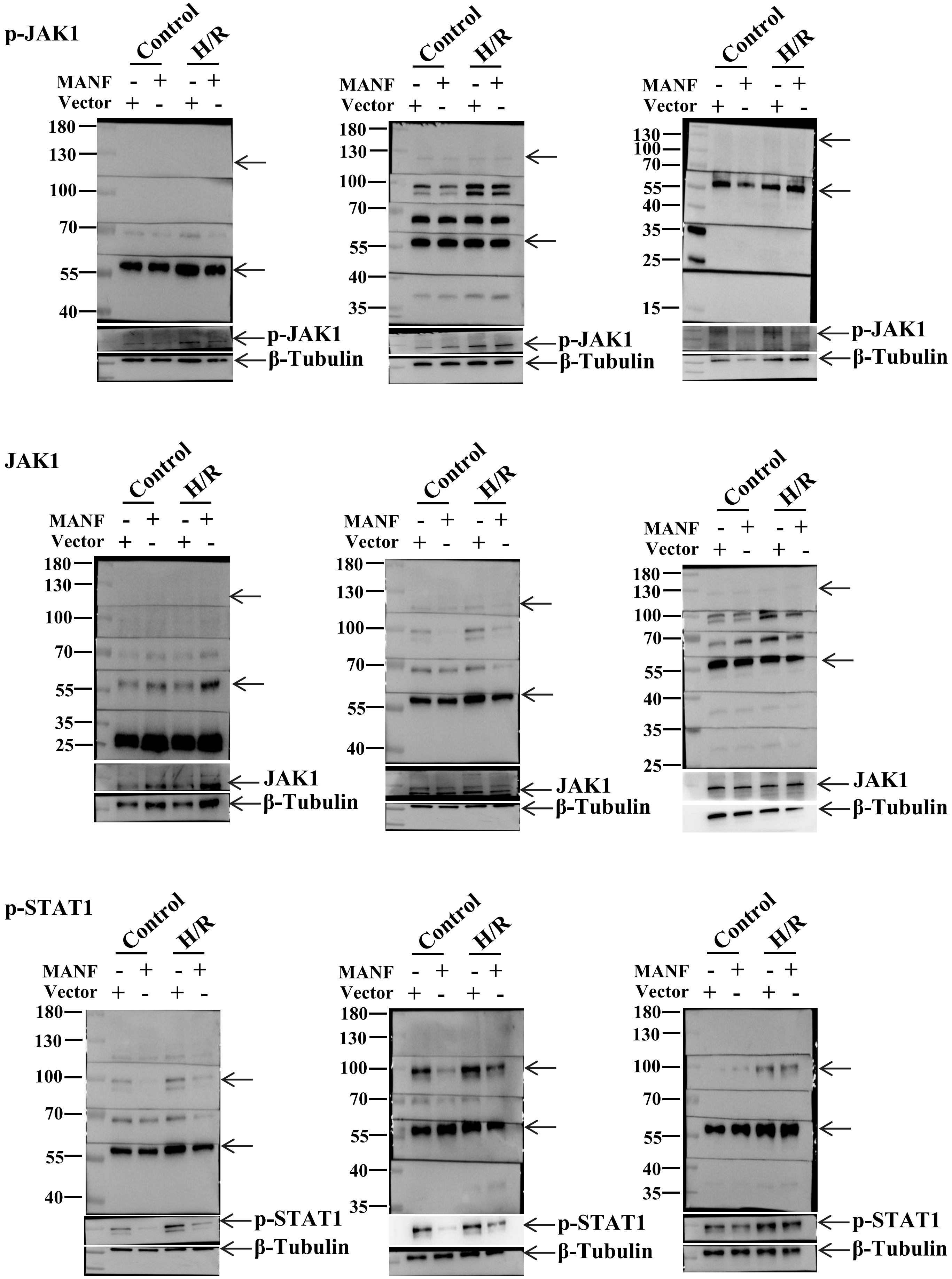

p-JAK1
Control
H/R
MANF - + - +
Vector + - + -
180
130
100
70
55
40
p-JAK1
β-Tubulin
Control
H/R
MANF - + - +
Vector + - + -
180
130
100
70
55
40
35
p-JAK1
β-Tubulin
Control
H/R
MANF - + - +
Vector + - + -
130
100
70
55
40
35
25
15
p-JAK1
β-Tubulin
JAK1
Control
H/R
MANF - + - +
Vector + - + -
180
130
100
70
55
35
25
JAK1
β-Tubulin
Control
H/R
MANF - + - +
Vector + - + -
180
130
100
70
55
40
JAK1
β-Tubulin
Control
H/R
MANF - + - +
Vector + - + -
180
130
100
70
55
40
35
25
JAK1
β-Tubulin
p-STAT1
Control
H/R
MANF - + - +
Vector + - + -
180
130
100
70
55
40
p-STAT1
β-Tubulin
Control
H/R
MANF - + - +
Vector + - + -
180
130
100
70
55
40
35
p-STAT1
β-Tubulin
Control
H/R
MANF - + - +
Vector + - + -
180
130
100
70
55
40
35
p-STAT1
β-Tubulin

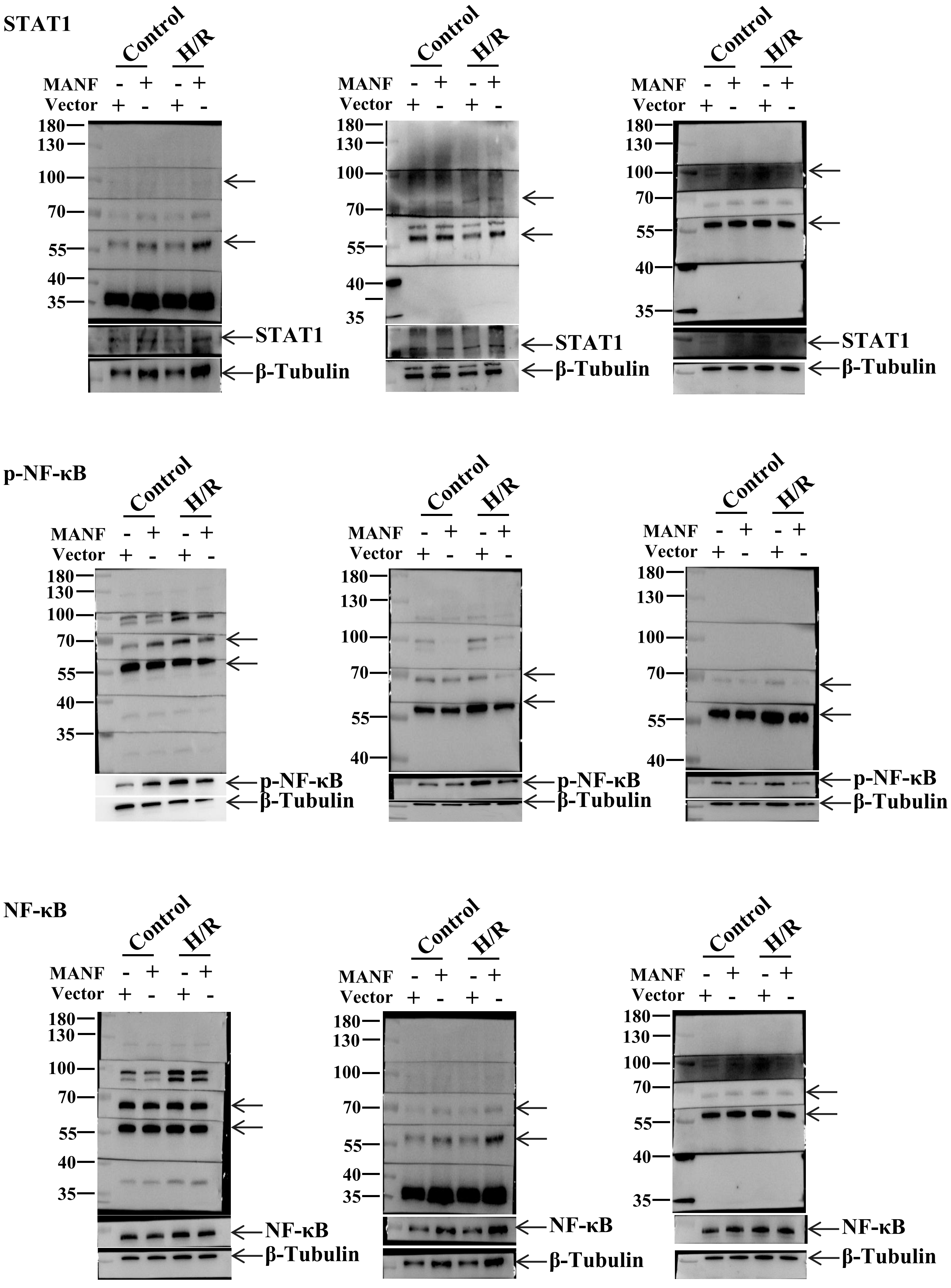
STAT1
Control
H/R
MANF - + - +
Vector + - + -
180
130
100
70
55
40
35
STAT1
β-Tubulin
p-NF-κB
Control
H/R
MANF - + - +
Vector + - + -
180
130
100
70
55
40
35
p-NF-κB
β-Tubulin
NF-κB
Control
H/R
MANF - + - +
Vector + - + -
180
130
100
70
55
40
35
NF-κB
β-Tubulin

p-JAK1

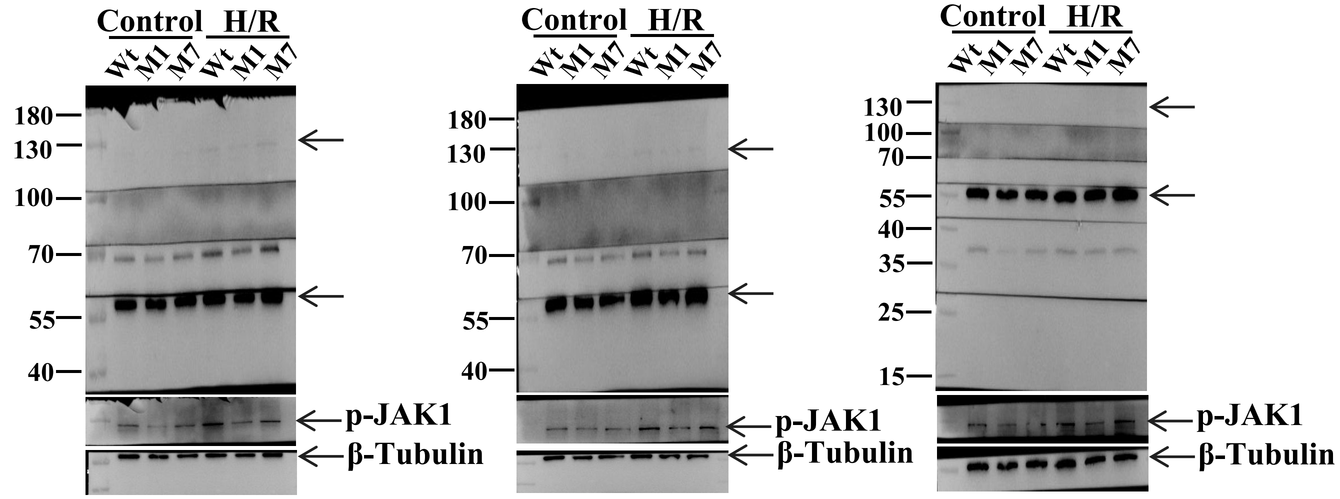

JAK1

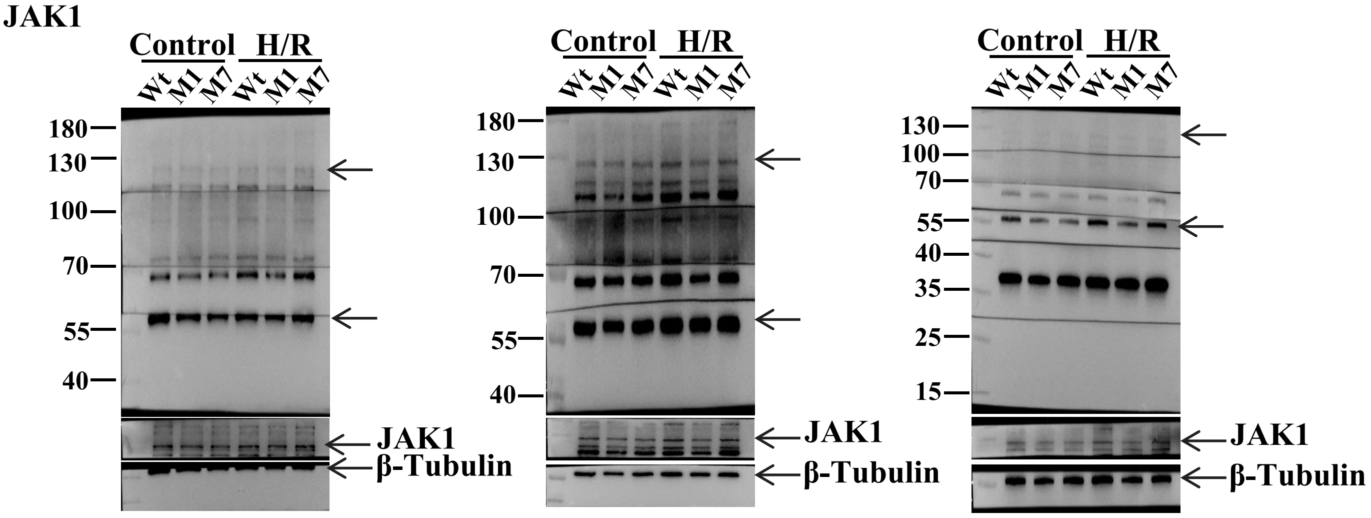

p-STAT1

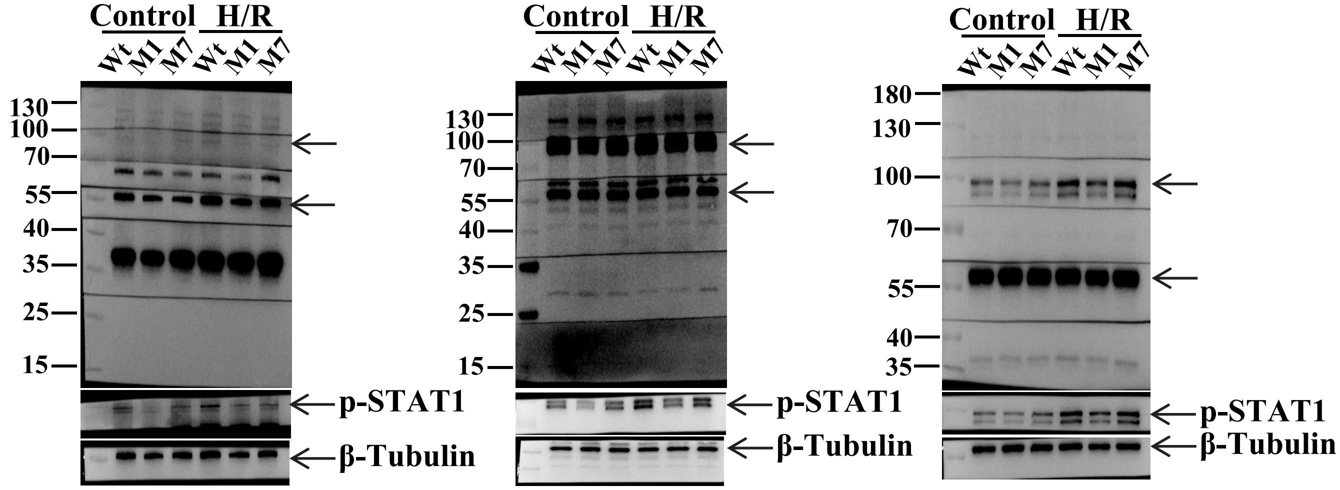

STAT1

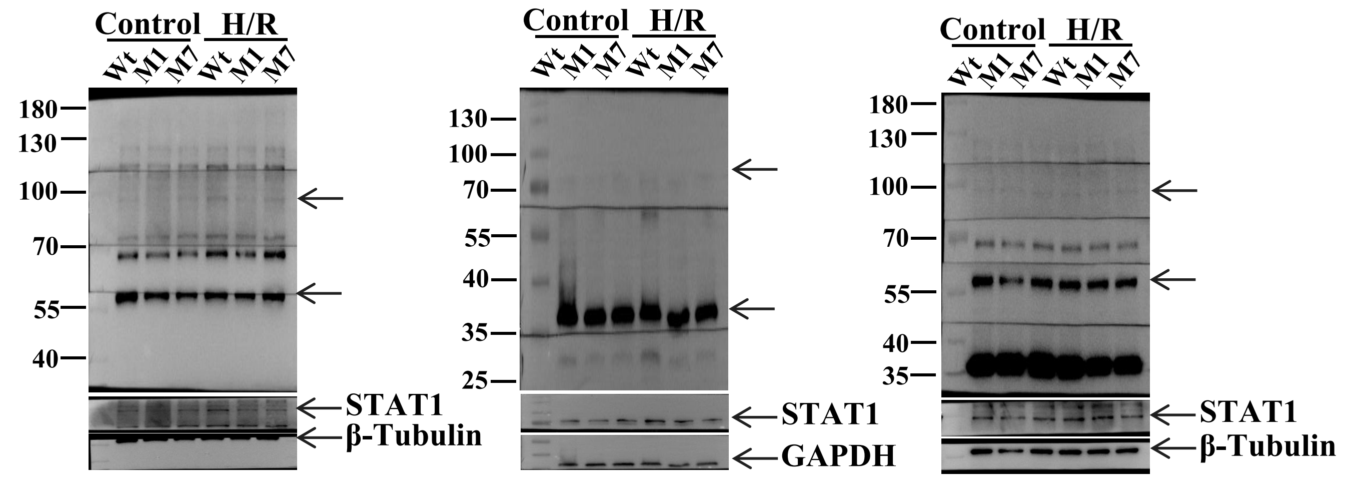

p-NF-κB

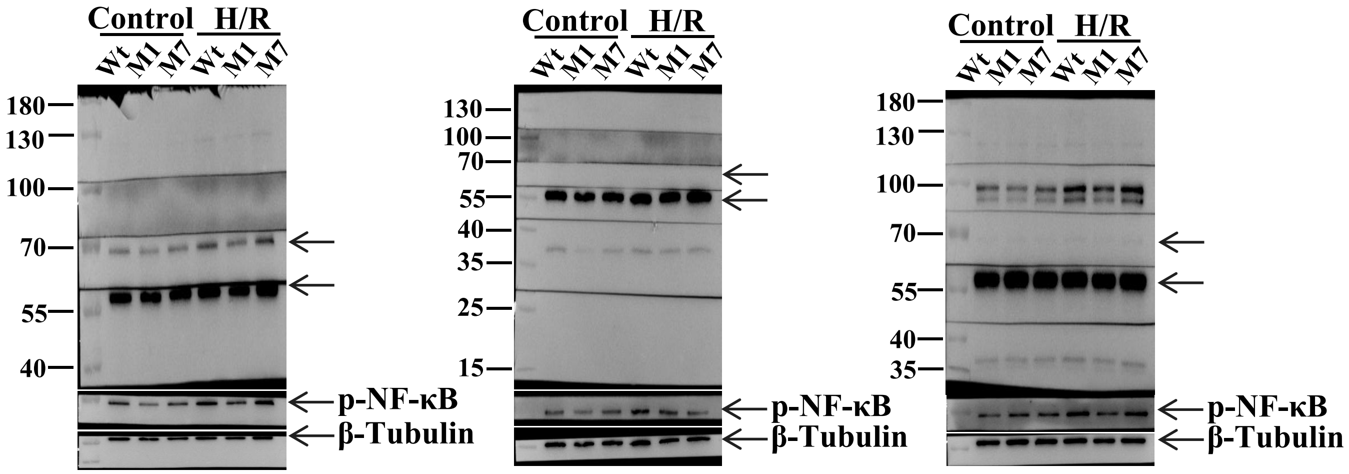

NF-kB

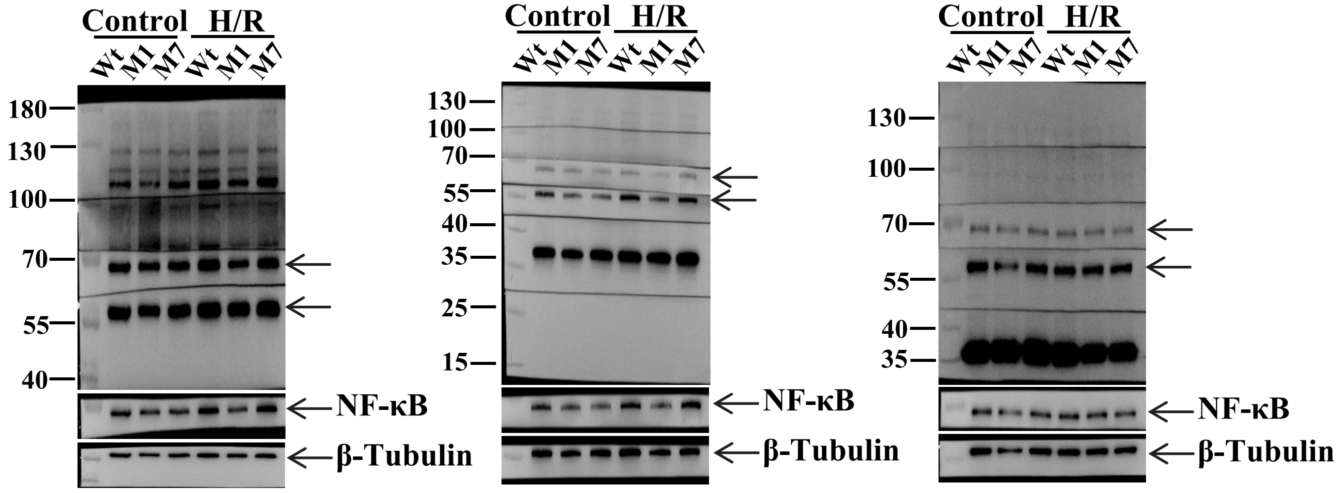

Figure 7 E

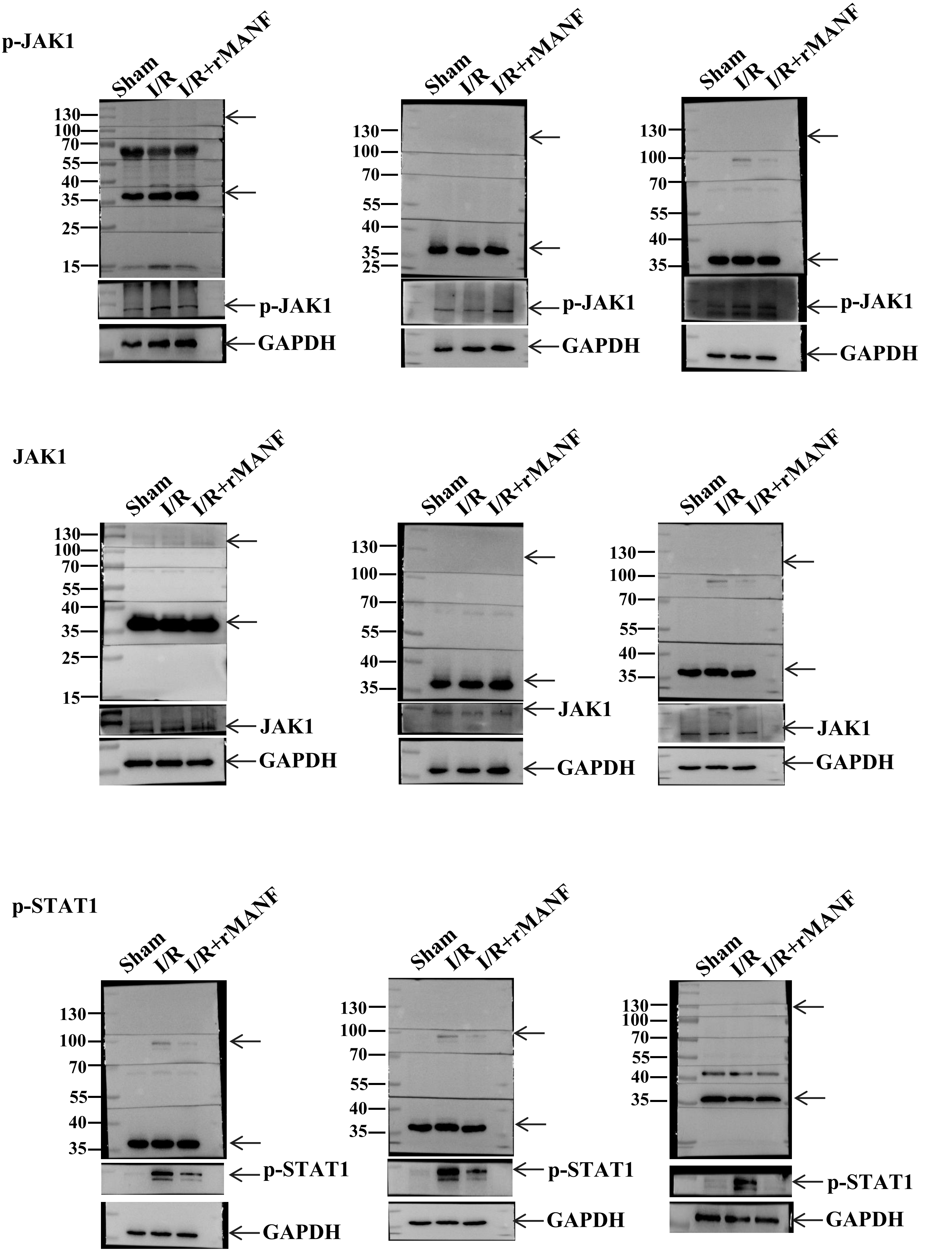
p-JAK1
Sham
I/R
I/R+rMANF
130
100
70
55
40
35
25
15
p-JAK1
GAPDH
JAK1
JAK1
p-STAT1
p-STAT1

STAT1

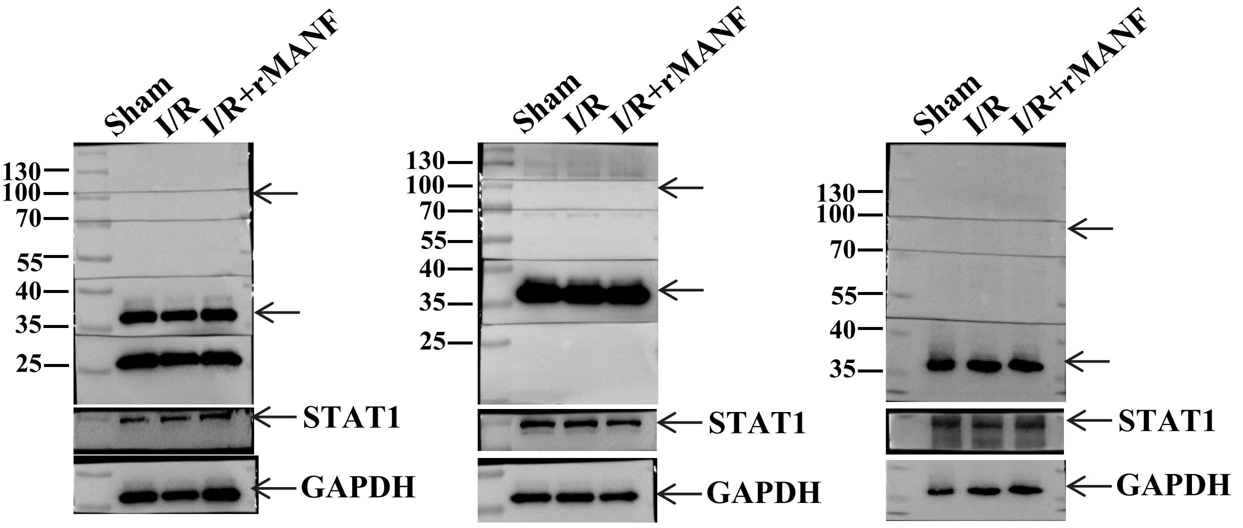

p-NF-κB

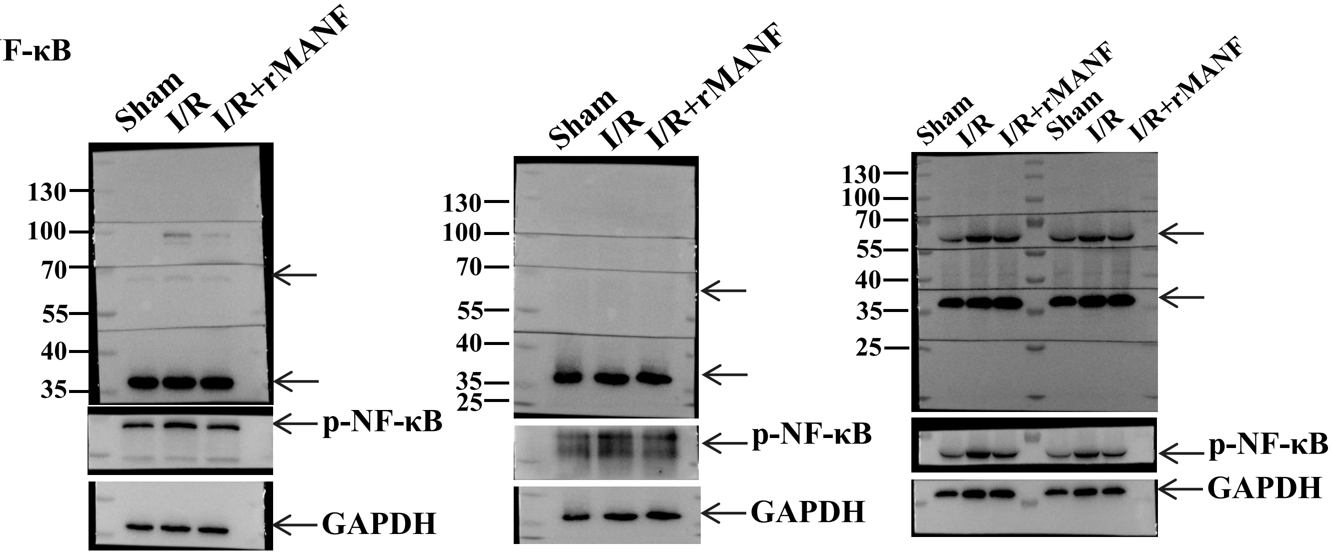

NF-κB

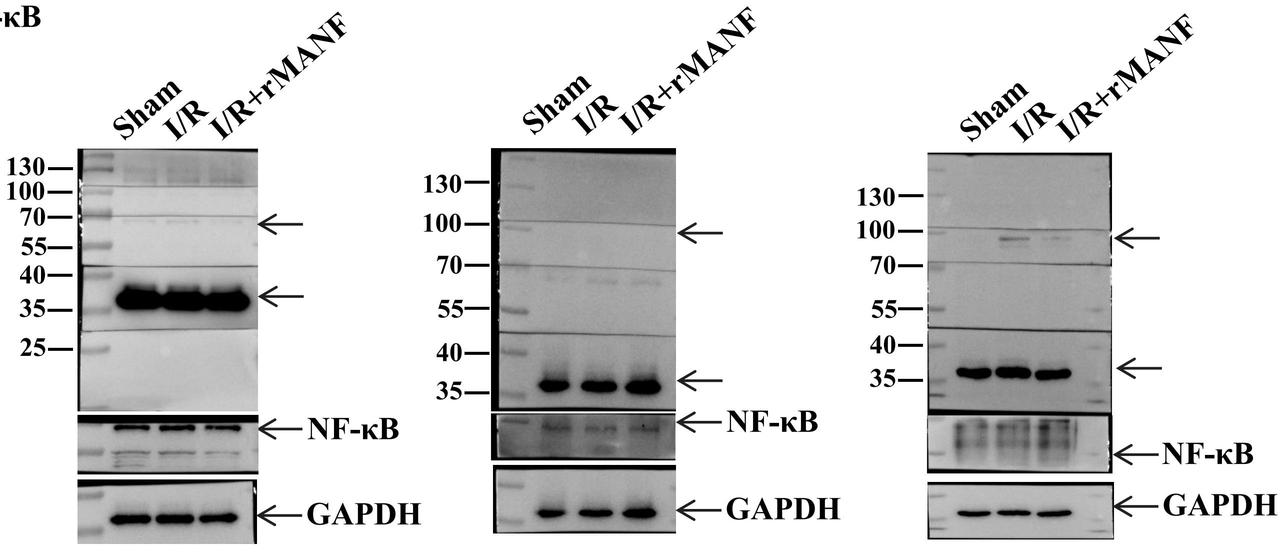

Figure S6

LC3B

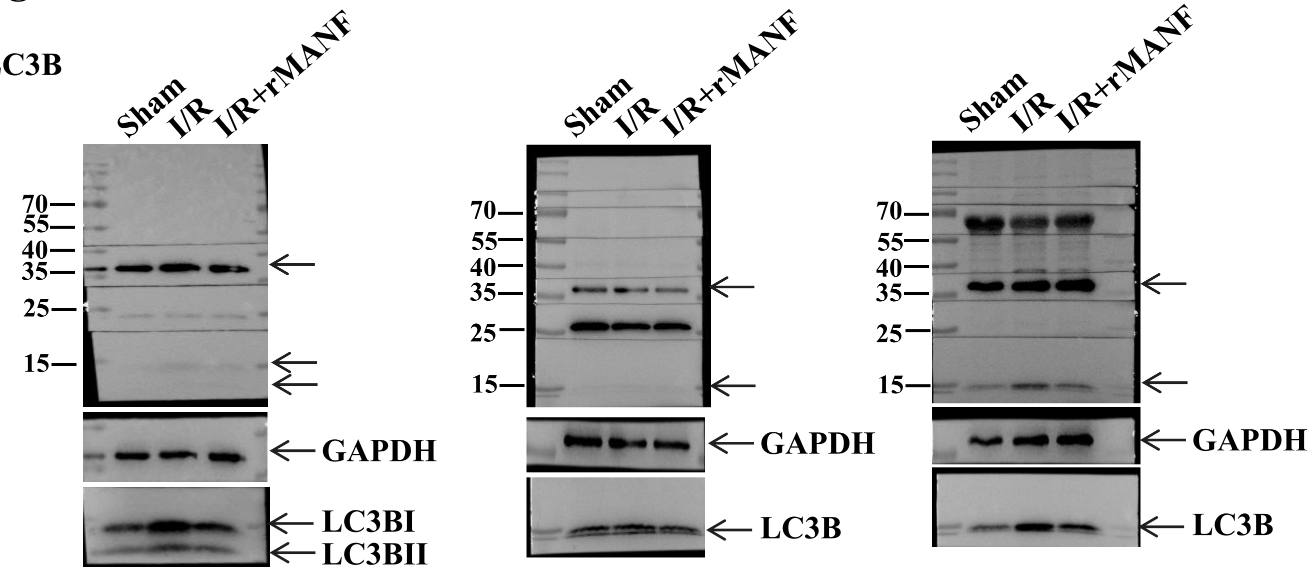

SQSTM1

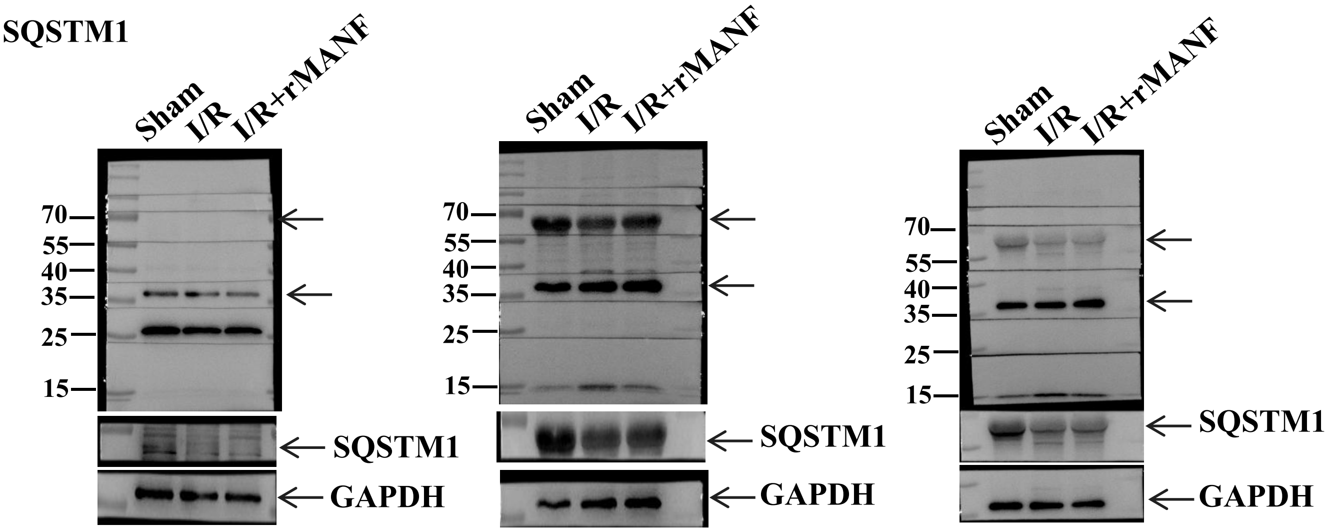

Supplement: Supplementary file 1 — Supplementary Material 1 [file 10020_2024_916_MOESM1_ESM.pdf]
