# Supplementary material for: Key subdomains of mesencephalic astrocyte-derived neurotrophic factor attenuate myocardial ischemia/reperfusion injury by JAK1/STAT1/NF-κB signaling pathway
Source: Mol Med. 2024 Sep 6;30:139. doi: 10.1186/s10020-024-00916-6 (PMC11380330; doi:10.1186/s10020-024-00916-6)
Supplement: Supplementary file 2 — Supplementary Material 2 [file 10020_2024_916_MOESM2_ESM.docx]

**Supplementary Material**

**Key subdomains of mesencephalic astrocyte-derived neurotrophic factor regulate its protective function on myocardial ischemia-reperfusion injury via the JAK1/STAT1/NF-κB pathway**

Haibin Dong^1*^, Wenjuan Jia^1*^, Chunxiao Wang^1^, Da Teng^1^, Bowen Xu^1^, Xiaoning Ding^2^, Jun Yang^1^, Lin Zhong^1^, Lei Gong^1^

^1^Department of cardiology, Yantai Yuhuangding Hospital, Qingdao University, Yantai, Shandong 264000, China

^2^Shanghai Children’s Medical Center, Shanghai Jiao Tong University School of Medicine, Shanghai 200127, China

^*^ These authors contributed equally to this work.


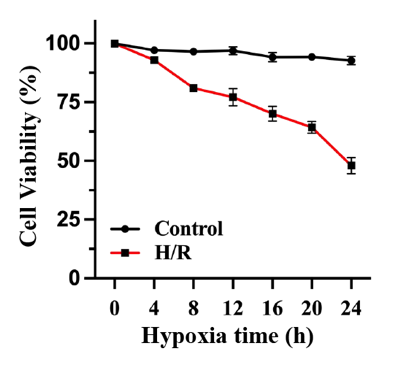


**Supplementary Fig. S1 Cell survival under varying durations of hypoxia.** HL-1 was exposed to hypoxic conditions for 4, 8, 12, 16, 20 and 24 hours followed by 3 hours of normal culture. Data are presented as mean ± SD and represent at least 3 independent experiments.


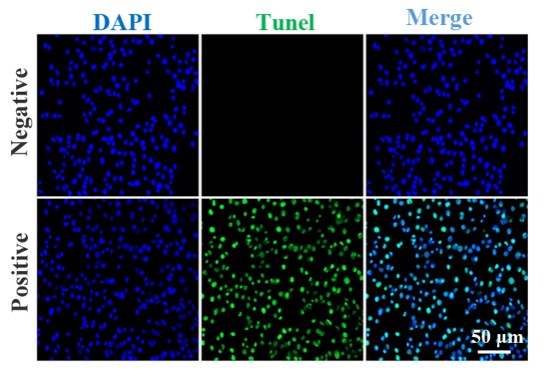


**Supplementary Fig. S2** Representative Tunel negative and positive control fluorescence images. Scale bar, 50 μm.

**
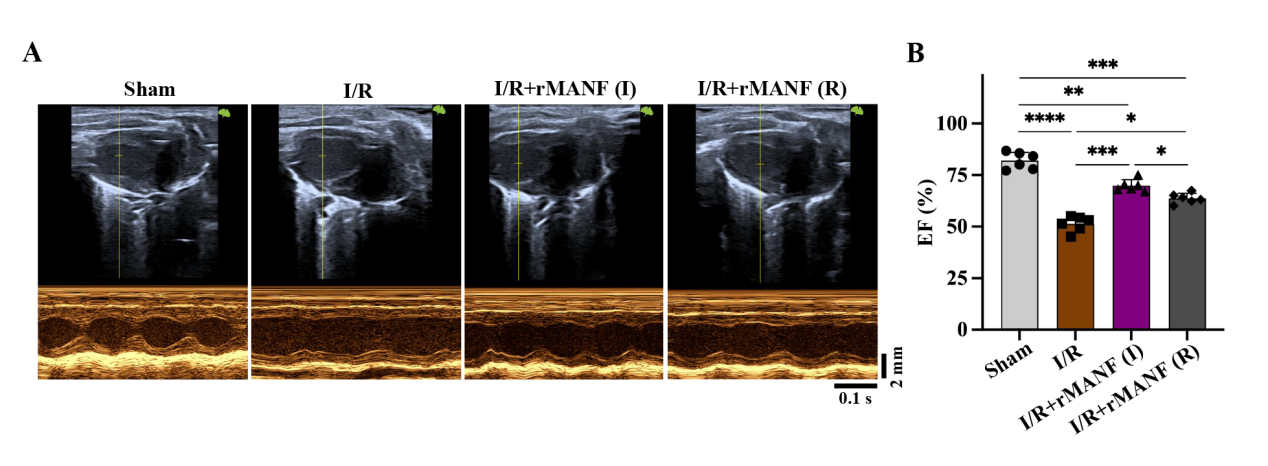
**

**Supplementary Fig. S3** Representative images of M-mode echocardiography and quantitative analysis of EF for rMANF injection during ischemia and reperfusion, respectively. Data are presented as mean ± SD, n = 6 mice per group, 3 independent experiments. Sham vs I/R, Sham vs I/R+rMANF, I/R vs I/R+rMANF, *P＜0.05, **P＜0.01, ***P＜0.001, ****P＜0.0001.


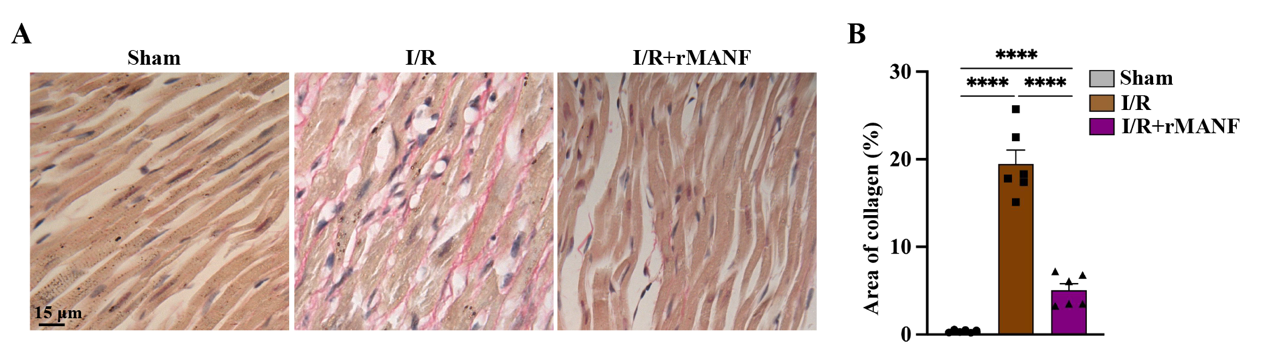


**Supplementary Fig. S4 Recombinant MANF protein reduces collagen fibers post-myocardial injury.** Representative images of Sirius red staining and statistical quantification of collagen fiber area. Recombinant MANF protein was administered during myocardial ischemia at a dose of 1.5 mg/kg. Collagen fibers were stained red. Data are presented as mean ± SD, with 6 mice per group and 3 independent experiments. Scale bar, 15 μm. Sham vs I/R, Sham vs I/R+rMANF, I/R vs I/R+rMANF, ****P＜0.0001.


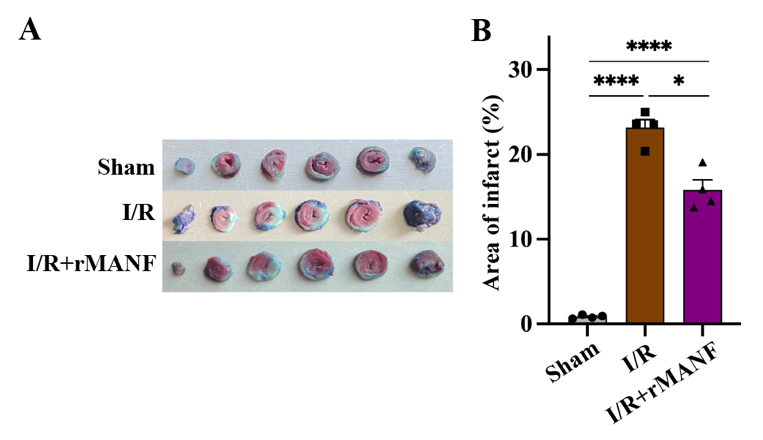


**Supplementary Fig. S5 Recombinant MANF protein can reduce the myocardial infarction area.** Representative images of Evans Blue-TTC double staining and statistical quantification of the infarct area. The infarcted region of the I/R heart were stained white. Data are presented as mean ± SD, with 4 mice per group and 3 independent experiments. Sham vs I/R, Sham vs I/R+rMANF, I/R vs I/R+rMANF, *P＜0.05, ****P＜0.0001.


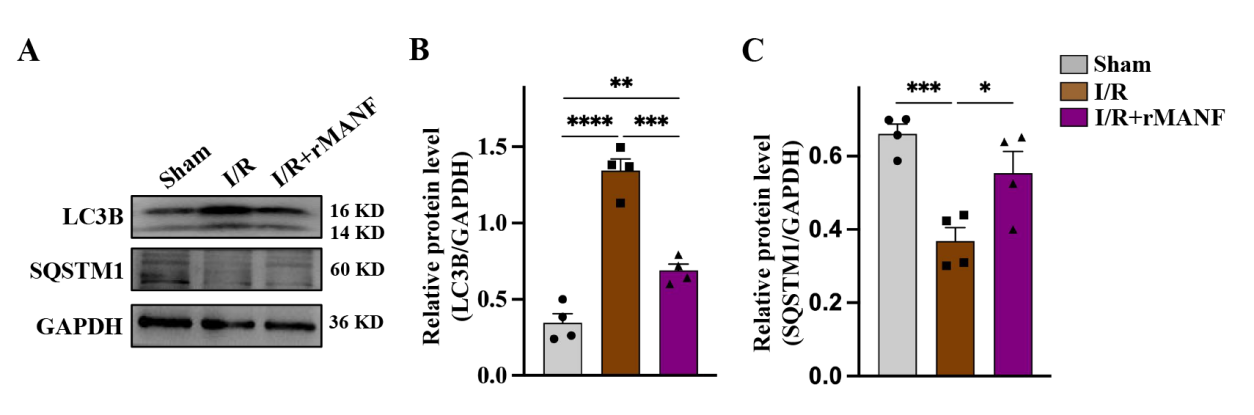


**Supplementary Fig. S6** Western blot analysis and statistical quantification of LC3B and SQSTM1 protein levels in I/R mice added with rMANF. The quantitative bands were normalized to GAPDH. Data are presented as mean ± SD, n = 4 mice per group, 3 independent experiments. Sham vs I/R, Sham vs I/R+rMANF, I/R vs I/R+rMANF, *P＜0.05, **P＜0.01, ***P＜0.001, ****P＜0.0001.

**Supplementary Table S1. Echocardiographic parameters of I/R and I/R+rMANF mice.**

| Paratemer | Sham（n=6） | I/R（n=6） | I/R+rMANF（n=6） |
| --- | --- | --- | --- |
| EF（%） | 77.55±2.98 | 50.35±2.83* | 63.88±1.95* |
| FS（%） | 43.41±2.52 | 27.05±1.14* | 33.32±1.49* |
| LVEDV（μL） | 35.11±2.29 | 47.64±1.65* | 41.12±1.14* |
| LVESV（μL） | 9.83±1.08 | 29.34±1.44* | 18.05±1.35* |
| SV（μL） | 23.57±1.93 | 34.35±1.23* | 27.08±1.14* |
| LVIDd（mm） | 2.94±0.18 | 3.85±0.09 | 3.27±0.05 |
| LVIDs（mm） | 1.82±0.11 | 2.94±0.08 | 2.32±0.07 |
| IVSd（mm） | 0.88±0.05 | 0.56±0.02 | 0.74±0.06 |
| IVSs（mm） | 1.14±0.03 | 0.85±0.03 | 1.07±0.07 |
| LVPWs（mm） | 1.29±0.03 | 0.98±0.06 | 1.19±0.05 |

Ejection fraction (EF), fractional shortening (FS), left ventricular end diastolic volume (LVEDV), left ventricular end systolic volume (LVESV), stroke volume (SV), left ventricular inner diameter in diastole (LVIDd), left ventricular inner diameter in systole (LVIDs), interventricular septal thickness at diastole (IVSd), systolic interventricular septal thickness (IVSs) and systolic left ventricular posterior wall thickness (LVPWs). Data are presented as mean ± SD and represent at least 3 independent experiments. Statistical comparisons include Sham vs I/R, Sham vs I/R+rMANF, I/R vs I/R+rMANF, with significance denoted as *P＜0.05.
